# Exploring the role of CBLB in acute myocardial infarction: transcriptomic, microbiomic, and metabolomic analyses

**DOI:** 10.1186/s12967-024-05425-y

**Published:** 2024-07-14

**Authors:** Hongjun You, Fengjun Chang, Haichao Chen, Yi Wang, Wenqi Han

**Affiliations:** Department of Cardiovascular Medicine, Shaanxi Provincial People’s Hospital, No.256 Youyi West Road, Beilin District, Xi’an City, 710068 Shaanxi Province China

**Keywords:** Acute myocardial infarction, RNA-seq, 16S rDNA sequencing, Non-targeted metabolites sequencing, Biomarkers, CBLB

## Abstract

**Background:**

Specific alterations in gut microbiota and metabolites have been linked to AMI, with CBLB potentially playing an essential role. However, the precise interactions remain understudied, creating a significant gap in our understanding. This study aims to address this by exploring these interactions in CBLB-intervened AMI mice using transcriptome sequencing, 16 S rDNA, and non-targeted metabolite analysis.

**Methods:**

To probe the therapeutic potential and mechanistic underpinnings of CBLB overexpression in AMI, we utilized an integrative multi-omics strategy encompassing transcriptomics, metabolomics, and 16s rDNA sequencing. We selected these particular methods as they facilitate a holistic comprehension of the intricate interplay between the host and its microbiota, and the potential effects on the host’s metabolic and gene expression profiles. The uniqueness of our investigation stems from utilizing a multi-omics approach to illuminate the role of CBLB in AMI, an approach yet unreported to the best of our knowledge. Our experimental protocol encompassed transfection of CBLB lentivirus-packaged vectors into 293T cells, followed by subsequent intervention in AMI mice. Subsequently, we conducted pathological staining, fecal 16s rDNA sequencing, and serum non-targeted metabolome sequencing. We applied differential expression analysis to discern differentially expressed genes (DEGs), differential metabolites, and differential microbiota. We performed protein-protein interaction analysis to identify core genes, and conducted correlation studies to clarify the relationships amongst these core genes, paramount metabolites, and key microbiota.

**Results:**

Following the intervention of CBLB in AMI, we observed a significant decrease in inflammatory cell infiltration and collagen fiber formation in the infarcted region of mice hearts. We identified key changes in microbiota, metabolites, and DEGs that were associated with this intervention. The findings revealed that CBLB has a significant correlation with DEGs, differential metabolites and microbiota, respectively. This suggests it could play a pivotal role in the regulation of AMI.

**Conclusion:**

This study confirmed the potential of differentially expressed genes, metabolites, and microbiota in AMI regulation post-CBLB intervention. Our findings lay groundwork for future exploration of CBLB’s role in AMI, suggesting potential therapeutic applications and novel research directions in AMI treatment strategies.

**Supplementary Information:**

The online version contains supplementary material available at 10.1186/s12967-024-05425-y.

## Introduction

Acute myocardial infarction (AMI) is a severe cardiovascular condition primarily resulting from unstable plaque rupture and erosion, instigated by coronary artery disease. These pathological changes can induce thrombosis, subsequently reducing coronary blood flow, causing prolonged myocardial ischemia, and ultimately leading to myocyte necrosis [1]. The American Heart Association reports that the overall incidence of AMI is approximately 3% [2]. Although there has been progress in AMI therapeutics, significant challenges remain in reversing myocardial injury, inhibiting post-injury myocardial remodeling, and reducing the incidence of postoperative adverse cardiovascular events [3, 4]. Thus, the search for new biomarkers to improve AMI diagnostics and therapeutics is of critical clinical significance.

Casitas B lymphoma-b (Cbl-b), an E3 ubiquitin ligase, is vital in the ubiquitination of proteins downstream of immune receptors, thereby inhibiting the positive signaling cascade [5]. This mechanism plays a central role in promoting peripheral T cell tolerance and suppressing autoimmunity [6], both of which are crucial in immune cell regulation. Cbl-b’s immunomodulatory functions, therefore, bear a direct relevance to AMI pathophysiology.

AMI is a condition characterized by an inflammatory response, in which immune cells play a pivotal role. The activation of T cells, in particular, has been recognized as a critical step in the inflammatory response following myocardial infarction. Given Cbl-b’s role in inhibiting the transcriptional activity of T cells [7] and promoting immune tolerance [8], its dysregulation may contribute to the exacerbation of AMI, thus providing a compelling rationale for focusing on this E3 ubiquitin ligase in our study.

Research has suggested that Cbl-b may serve as a potential biomarker for AMI, which provides evidence that Cbl-b expression is significantly downregulated in AMI patients compared to the control group, as demonstrated by both public database analysis and analysis of 20 peripheral blood samples [9]. These findings further underscore the potential role of Cbl-b in the pathogenesis and progression of AMI. Based on this perspective, we postulate that during the transition from a healthy state to AMI, there is a progressive downregulation of the Cbl-b gene. Consequently, we proposed a hypothesis: if we were to increase Cbl-b expression in AMI patients, could this potentially serve as a countermeasure against the progression of AMI, potentially steering the patient back towards a healthier state? This hypothesis led us to explore, in our study, the effects of enhancing Cbl-b expression as a potential therapeutic strategy in AMI. Furthermore, Cbl-b’s high expression in immune cells, its ability to ubiquitinate and degrade receptor-activated signaling proteins [6], and its immunomodulatory functions, have made it a compelling target for studying AMI pathophysiology. These distinctive characteristics of Cbl-b lend further weight to our hypothesis and the relevance of our study in the context of AMI.

**Fig. 1:**
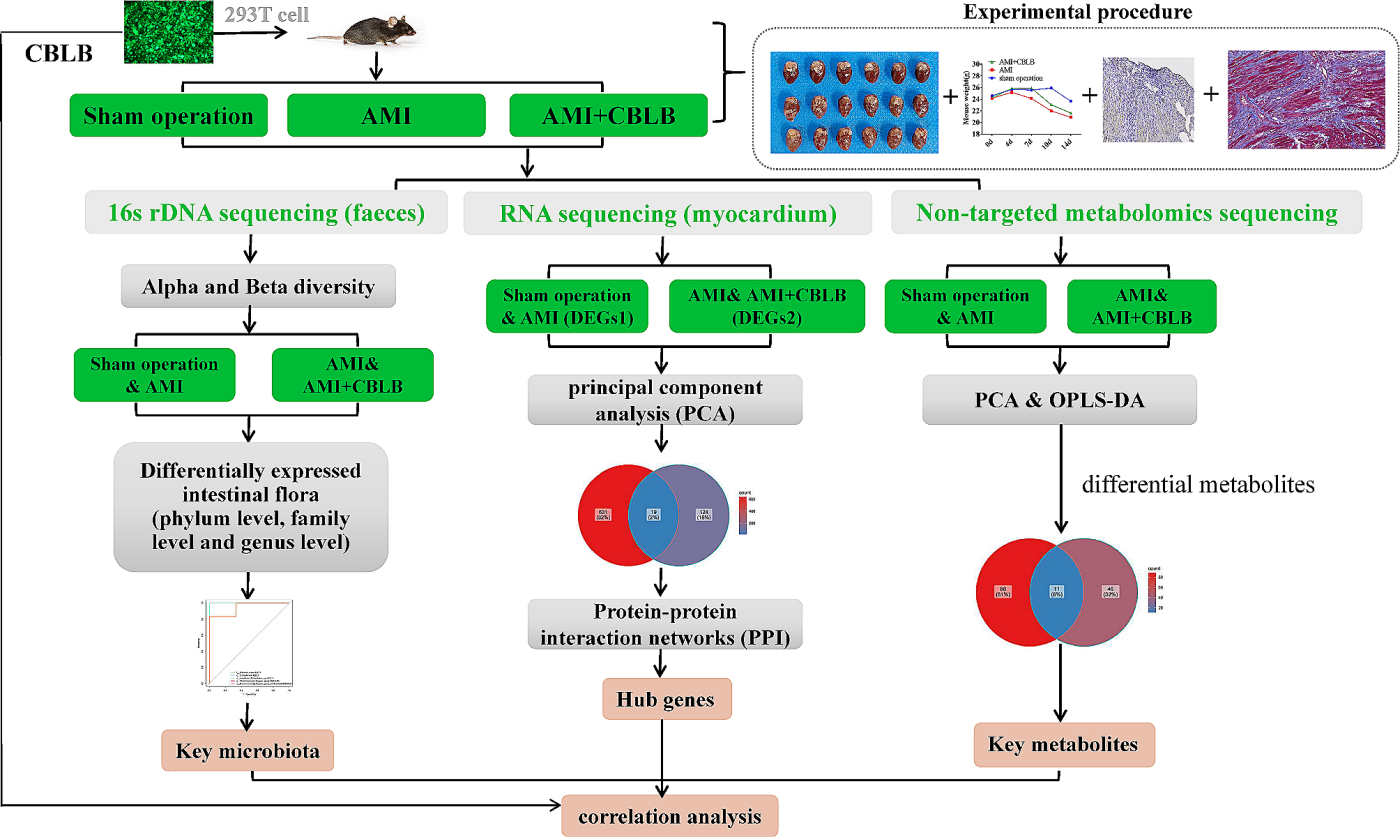
**Research Flowchart.** Integration process of multi-omics data and bioinformatics technology

Recent research has revealed specific changes in the gut microbiota and in vivo metabolites in AMI patients [9–11], providing new perspectives on AMI diagnosis and treatment. However, the role of Cbl-b in these alterations remains largely unexplored. This limitation presents a significant research gap, which this study aims to address. Based on preliminary data and literature reviews, we hypothesize that Cbl-b plays a critical role in modulating these changes. To test this hypothesis and potentially fill this research gap, we are using bioinformatic tools such as differential expression analysis and enrichment analysis to investigate transcriptome, 16 S bacterial flora sequencing, and non-targeted metabolomics sequencing data from three distinct mouse treatment groups. We aim to thoroughly investigate the expression, differential changes, and functional regulation of key genes, bacterial flora, and metabolites in AMI mice following Cbl-b intervention **(**Fig. 1**)**. Through this study, we strive to reveal the potential regulatory mechanisms of Cbl-b in AMI, thereby contributing to the novelty of our study and the broader understanding of AMI. We anticipate that our findings could provide new insights into AMI pathophysiology and lead to potential new therapeutic targets. By linking our methodological approach to these potential clinical and translational impacts, we underscore the relevance and potential significance of our study in the field of AMI research. This study, therefore, not only aims to elucidate the relationship between Cbl-b, gut microbiota, metabolites, and AMI but also to potentially provide novel insights into AMI diagnosis and treatment.

## Materials and methods

### Packaging of CBLB overexpression lentiviral vectors

The frozen Human embryonic kidney (293T) cells were immediately rewarmed at 37 °C, put in complete medium containing 10 ml, and centrifuged at 1000 r/min for 5 min at room temperature. After removing the supernatant, the cells were put to DMEM complete media and transferred to T-25 culture flasks, where they were cultivated at 37 °C with 5% CO_2_.

After putting 293T cells in 4–5 × 10^6^/10 cm dishes, incubate them for 24 h at 37 °C with 5% CO_2_. Plasmid No. 1 and the transfection reagent dilution No. 2 should be made in the following order **Supplementary Table 1**. After thoroughly combining No. 2 and No. 1 reagents, incubate the mixture at room temperature for 15 min. Drop by drop, 1 ml of the transfection mixture was added to the 293T cells when the cell density reached 80-90%. Followed by, replace 10 ml of fresh 293T medium and virus medium 4–6 hours and 24 h after transfection, respectively. Finally, cell culture supernatants were collected at 48 h post-transfection and centrifuged at 500 g for 10 min, after which the supernatants were collected for lentivirus infection. Upon transfection of the 293T cells with CBLB lentivirus, the fluorescence intensity surpassed 95%, demonstrating a high transfection efficacy **(Supplementary Fig. 1)**.

### Construction of acute myocardial infarction (AMI) model mice

Male C57BL/6 mice aged 6–8 weeks were divided into three groups: A: sham operation; B: AMI; C: AMI + CBLB (*n* = 10/group). In brief, the randomization process was performed by an independent researcher who had no involvement in the data collection or analysis. Then, mice in each group were weighed, anesthetized, and fixed in the supine position before dehairing, skin preparation and disinfection, and transtracheal intubation. After tracheal intubation, they were connected to a small-animal ventilator with positive-pressure ventilation at a tidal volume of 20 ml/kg and a frequency of 120 breaths/min. Following that, the skin of the mice’s precordial region was clipped, the muscle was separated bluntly, and the chest wall was clipped at the third-fourth intercostal space. In groups B and C mice, the anterior descending branch of the left coronary artery was ligated with a 7 − 0 strip suture needle 3–4 mm below the inferior border of the left auricle. The whitening of the myocardium in the area below the ligation site suggested successful ligation. Group A mice had their chests opened but the anterior descending branch was not ligated. **Supplementary Fig. 2** illustrated the critical procedures involved in the surgical operation. Concurrently, in group C mice, 50u of CBLB viral solution (1.00E + 12 V.G.) was injected into the infarct site in 2–5 injections with an insulin syringe **(Supplementary Fig. 3)**. The dosage of the CBLB viral solution (50u of 1.00E + 12 V.G.) was determined based on a synthesis of our preliminary studies and previous literature [12]. Specifically, we found this dosage to be optimal for achieving the desired therapeutic effect without inducing significant adverse effects. Followed by, the chest cavity was closed and the chest was squeezed to eliminate chest gas, and the mice were placed in a 40 °C warming pad to await awakening. The tissue samples were collected on the 14th day following the surgery of AMI model construction. Prior to sampling, each group of mice was weighed, and three mice heart tissues were collected from each group for TTC staining. Ultimately, the apical Sect. (2/3) of cardiac tissue was stained, and the remaining 1/3 was used for transcriptome sequencing. Mice feces and serum samples were taken for 16s rDNA sequencing and Non-targeted metabolome sequencing, respectively.

### Myocardial tissue HE staining

The apical portion (2/3) of myocardial tissue was taken and fixed in 4% paraformaldehyde solution, followed by dehydration, embedding and sectioning. For HE staining, myocardial tissues were hydrated using an alcohol gradient after baking and deparaffinization, then stained with hematoxylin and eosin for 5 min and 5 to 10 s, respectively, before being dehydrated, translucent, and sealed.

### Myocardial tissue Masson staining

For Masson staining, the pre-treatment was the same as the HE staining procedure. Sections were then sequentially placed in Masson A solution (incubated at 65 °C oven for 30 min after overnight immersion at room temperature), Masson B and C mixture (1 min), 1% hydrochloric acid alcohol (1 min), Masson D (6 min), Masson E solution (1 min), Masson F solution (2–30 s), rinsed and differentiated in three consecutive vats of 1% glacial acetic acid (8 s per vat), three consecutive vats of anhydrous ethanol for sequential dehydration (approximately 5 s, 10 s, and 30 s), and finally clear and neutral gum sealing using xylene.

### Myocardial tissue immunohistochemical staining

For immunohistochemical staining, the pre-treatment was the same as the HE staining procedure. The sections were first antigenically repaired and blocked, then CBLB antibody was diluted as directed by the antibody manufacturer and incubated with the sections in a refrigerator at 4 °C overnight. The following day, the sections were placed in a 37 °C warmer to rewarm for 30 min, after which goat anti-mouse/rabbit IgG secondary antibody was added and incubated at 37 °C for 20 min. The final steps included DAB color development, hematoxylin restaining, dehydration, transparency, and sealing.

### 16s rDNA sequencing

The OMEGA Stool DNA Kit was used to extract total mouse fecal microbial DNA, which was then used in a one-step PCR (tuned up for 35 amplification cycles) with the Phusion enzymeand primers designed to match conserved areas in the 16 S rDNA sequence. Sequencing universal connectors and sample-specific Barcode sequences were added to the products amplified from the target region by PCR. Following that, PCR amplification products were detected using 1.5% agarose gel electrophoresis, and target fragments were retrieved using the AxyPrep PCR Cleanup Kit recovery kit. The purified PCR products were then measured and combined with the library using the Quant-iT PicoGreen dsDNA Assay Kit on a Promega QuantiFluor Fluorescence Quantification System. Finally, we used the Illumina Sequencing Platform 250PE to perform double-end sequencing using conventional methods.

### 16s rDNA sequencing data pre-processing

Relying on the Barcode information, the bipartite data collected from various samples by sequencing were first eliminated from splices and Barcode sequences. Cutadapt (v1.9) and FLASH (v1.2.8) software were implemented for data splicing and filtering. With the DATA2 method in the QIIME2 software program, the valid sequences of the samples were then aggregated and grouped into Amplicon Sequence Variants (ASVs). In order to comprehend the similarity and specificity in distinct groups of samples, we used the Venn diagram to acquire the ASVs that were common and unique across different groups. When the count value of an ASV was more than zero, it was assumed that the ASV was detected in that group of samples.

### Alpha and Beta diversity analysis

In order to obtain the diversity of microbiota within and between groups of mice in various treatment groups, we conducted Alpha and Beta diversity analysis. The variety and abundance of species in various samples and subgroups were first confirmed with the 16 S rDNA sequencing data by generating rarefaction curves. Then, we estimated the Alpha diversity in different subgroups of data and assessed the intergroup differences using observed features, Shannon, Simpson, chao1 and Pielou’s evenness index. Simultaneously, we used the Euclidean distance matrix based on 16 S rDNA sequencing data to perform principal coordinates analysis (PCoA) and Non-metric multidimensional scaling (NMDS, stress < 0.1) analysis showing the differences among subgroups, and analysis of Similarities (ANOSIM) was also carried out.

### Differential investigation of intestinal microbiota composition in various strata

We looked at differences in microbial community composition among the three groups of samples at the phylum, family, and genus levels, respectively, and plotted TOP10 bar stacks. Subsequently, we discovered which microbiota’ abundances between the B & A and C & B comparison groups differed considerably (LDA score > 2, *P* < 0.05) from each other by linear discriminant analysis (LDA) effect size (LEfSe). Employing the key microbiota from the differential microbiota screened in the two comparison groups, we next performed ROC curve analysis to evaluate the key microbiota’s capacity to discriminate between the two comparison groups.

### Non-targeted metabolomics sequencing

The 100 mg of fecal samples from each group of mice were weighed, ground in liquid nitrogen, and then mixed with 120 L of 50% methanol before being left to stand at room temperature for 10 min. With centrifuging the extracts at 4,000 g for 20 min after being chilled to -20 °C overnight, the top layer of metabolite extracts was transferred to a 96-well plate (10 L of each sample’s diluent was aspirated in an equal volume and mixed to create a quality control (QC) sample).

Using Proteowizard’s MSConvert software, the raw data were transformed into readable data mzXML, and the peaks were extracted using XCMS software. The extracted substances were annotated with summed ions using CAMERA, and then the extracted peaks were post-processed using metaX software, which mainly included metabolite-level identification and quantitative analysis. Metabolite ion secondary annotation was carried out with mass spectrometry primary information matched with the in-house standard database. Candidate identifiers were annotated with metabolite annotations using the human metabolome database or HMDB (https://hmdb.ca), Kyoto Encyclopedia of Genes and Genomes (KEGG) and other databases to explain the physicochemical properties and biological functions of metabolites, respectively. Quantification of differential metabolites and differential metabolite screening were performed using metaX software.

### Screening for differential metabolites

First, with SIMCA-P software, we conducted PCA and orthogonal partial least squares-discriminant analysis (OPLS-DA) on the metabolite data of the B & A and C & B comparator groups, respectively. A permutation test (*n* = 200) was subsequently utilized to validate the OPLS-DA model. According to this model, an S-plot was also made to look for difference metabolites across comparison groups (VIP > 2 and *P* < 0.05). In the end, by crossing the differential metabolites of the two comparison groups, we were able to determine the key metabolites.

### RNA sequencing (RNA-seq)

RNA from all samples was extracted and purified using TRIzol (Invitrogen, CA, USA) in line with the manufacturer’s suggested operating protocol. After that, a NanoDrop ND-1000 (NanoDrop, Wilmington, DE, USA) was utilized to assess the RNA’s quality. Then, via a Bioanalyzer 2100 (Agilent, CA, USA) and an agarose electrophoresis technique, the RNA’s integrity was evaluated. Downstream experiments were fulfilled by concentrations > 50 ng/L, RIN values > 7.0, OD260/280 > 1.8, and total RNA > 1 g. Specifically, PolyA (polyadenylate)-containing mRNA was isolated using oligo (dT) magnetic beads, reverse transcribed to DNA, and then sequenced on the Illumina Novaseq 6000 platform utilizing the bipartite PE150 sequencing mode.

### RNA-seq data pre-processing

Trimmomatic (v 0.39) was employed to filter low-quality data, and FastQC (v 0.11.9) was deployed to assess data quality [13]. Then, applying hisat2 (v 2.2.1) with all parameters set to default, clean Data was compared to the reference genome (GRCh37) [14]. Gene counts are extracted by FeatureCount (v 2.0.0) [15]. Ultimately, we utilized DESeq2 software to normalize the gene expression matrix obtained for each sample and presented expression box plots and PCA analysis.

### Acquisition and functional evaluation of differentially expressed genes (DEGs) in different comparison groups

DEGs1 and DEGs2 between B & A and C & B groups could be found in the RNA-seq via DESeq2 (v 1.26.0), respectively [16]. Significance thresholds were set as |log_2_FC| > 0.5 and *adj.P* < 0.05. Heatmaps and volcano plots depicting the expression of DEGs were displayed using ggplot2 (v 3.3.2) and pheatmap (v 4.1.0), respectively [17, 18]. Gene Ontology (GO) and KEGG were performed to further investigate the functions of DEGs1 and DEGs2 in RNA-seq data via ClusterProfiler (*P* < 0.05) (v 4.0.2) [19]. Besides, the Protein-protein interaction (PPI) network of intersecting gene, which gained by overlapping DEGs1 and DEGs2, was constructed using a tool to search for recurring instances of neighbouring genes (STRING, https://string-db.org) (medium confidence = 0.4) [20]. The interaction information was imported into Cytoscape software for visualization. The Hub genes were then screened for the PPI network utilizing the MCODE tool in Cytoscape (degree threshold = 2, maximum depth = 100, k-core = 2, node score cutoff = 0.2).

### Multi-omics association analysis of key microbiota-hub genes-key metabolites

In each of the three sets of sequencing data, we first assessed the expression of key microbiota, metabolites, and genes. After CBLB therapy of AMI, we undertook Spearman correlation analysis on the key microbiota, metabolites, and genes to investigate the relationship between gut microbiota impacting metabolite and even gene regulation in the three groups of mice (*P* < 0.05, *R* > 0.3). Correlations of CBLB with DEGs, differential microbiota and metabolites in the B & A and C & B comparison groups were also calculated separately.

### Statistical analysis

The research team who conducted the data analysis was not aware of which group the data belonged to until the statistical analysis was complete. Partial bioinformatic projects were conducted in R software. The Wilcoxon test was utilized to compare data from different groups. The Spearman method was utilized for correlation analysis. At *P* < 0.05, differences were considered significant.

## Results

### Myocardial infarction in AMI mice is dramatically reduced by CBLB

**Fig. 2:**
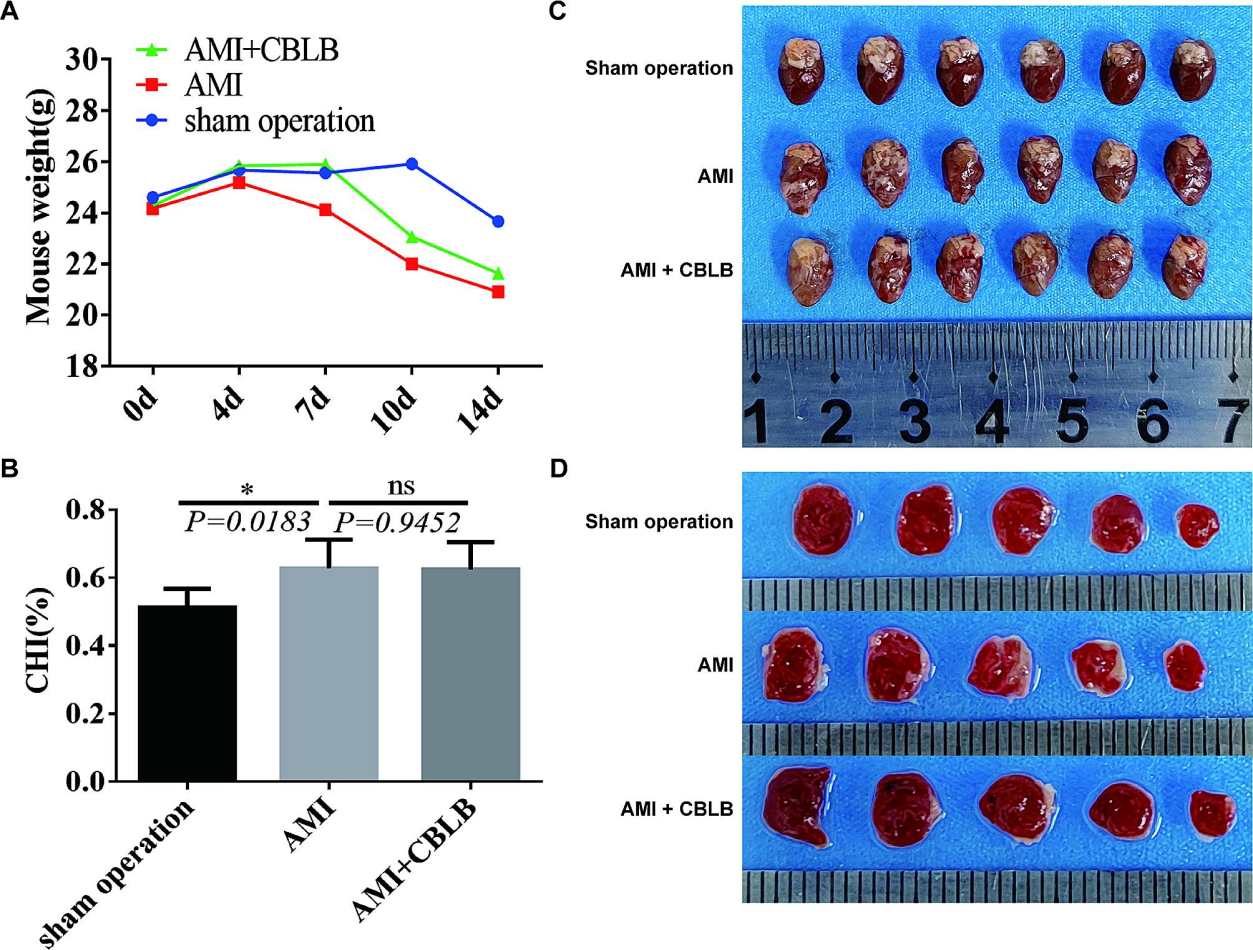
**Impact of CBLB overexpression on body weight and cardiac morphology in mice with AMI. A** Post-surgical body weight fluctuations across 0–14 days in the Sham, AMI, and AMI + CBLB overexpression mice groups, demonstrating a trend towards weight loss, particularly significant in the AMI group. **B** Comparative analysis of the cardiac hypertrophy index level among the three mice groups, revealing that AMI group has a significantly larger index compared to the Sham group, suggesting the impact of acute myocardial infarction on myocardial hypertrophy, with no significant difference observed between AMI and AMI + CBLB groups. **C** Photographic documentation of the overall heart morphology in the three mice groups, showing visible infarcted tissue in the left ventricular wall of AMI and AMI + CBLB groups. **D** Representative images of TTC staining of infarcted areas of the myocardium (infarcted area is in white), illustrating a deeper penetration of the infarcted area into the inner layer of the left ventricular wall in AMI and AMI + CBLB groups, with some reduction in the infarcted area observed in the AMI + CBLB group. These observations signify the potential protective role of CBLB in AMI. All the data are expressed as mean ± SEM; *n* = 6 per group. * *P* < 0.05, ns, no statistical significance

Groups A, B, and C all exhibited a trend towards weight loss, with the most significant reduction observed in group B **(**Fig. 2A**)**. This outcome implied that the infarction somewhat reduced the rate of body weight gain. Myocardial hypertrophy index results indicated that the B group had a considerably larger index than the A group, demonstrating that acute myocardial infarction in mice would affect the index of their myocardium’s hypertrophy (Fig. 2B). Subsequently, visual observation of myocardial tissue **(**Fig. 2C**)** and TTC staining **(**Fig. 2D**)** maps of mice in each group showed that compared with the A group, there was obvious infarcted tissue in the left ventricular wall of the B group and the C group, and the TTC showed that the infarcted area penetrated deeper into the inner layer of the left ventricular wall, whereas some of the mice in the C group observed a reduction in the infarcted area.

**Fig. 3:**
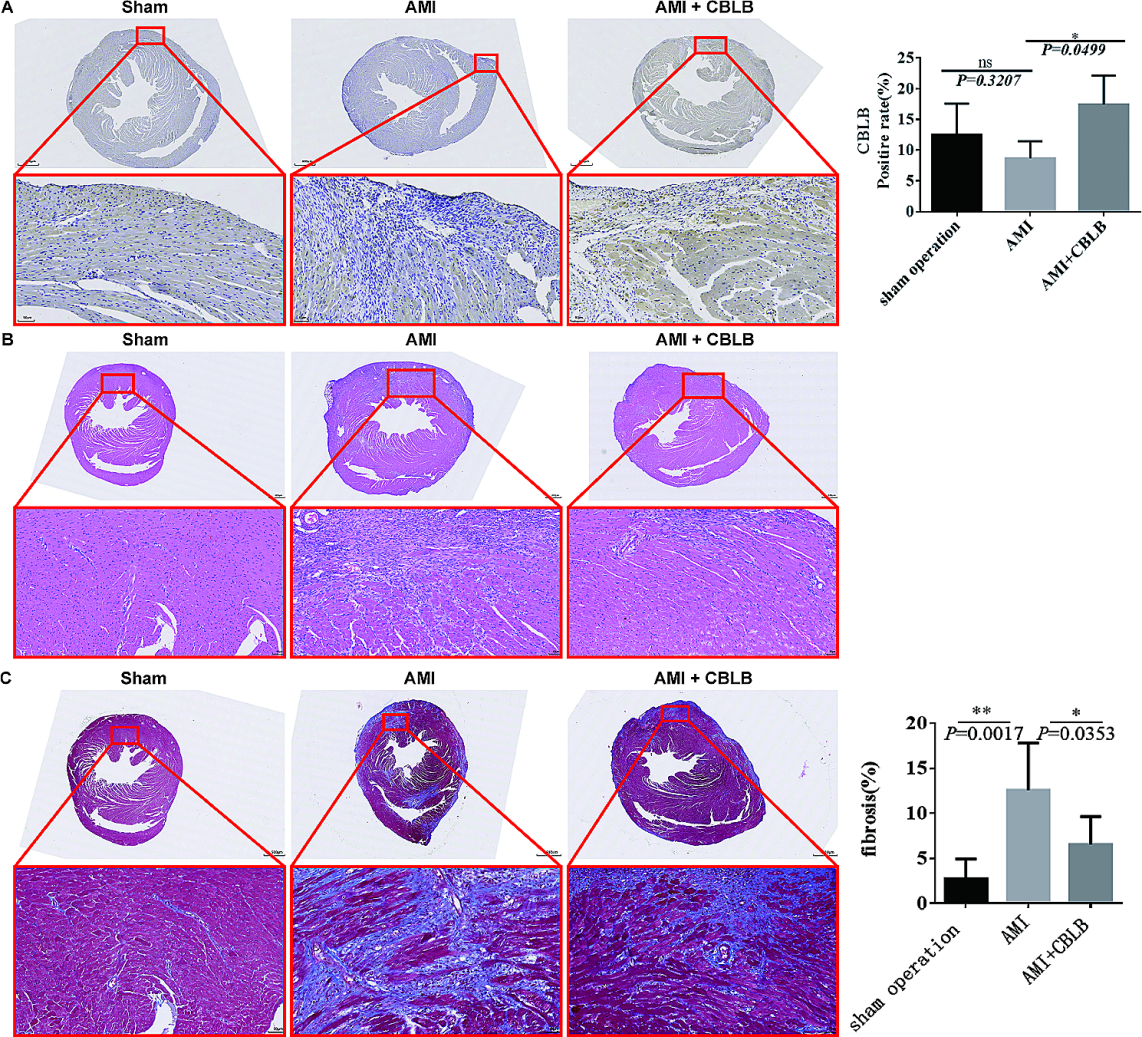
**Overexpression of CBLB ameliorates myocardial infarction in mice with AMI. A** Representative images of immunohistochemical staining of CBLB in myocardial tissues drawn from three distinct groups of mice, accompanied by a quantification of CBLB expression levels in each group. The increased expression in the C group suggests the protective role of CBLB in myocardial tissues. **B** Representative HE staining of heart sections derived from three distinct groups of mice. The observed nucleolysis and consolidation of cardiomyocytes in the B group compared to the A group highlights the damaging effects of AMI, which are mitigated by CBLB overexpression. **C** Representative images of Masson’s Trichrome staining of heart sections obtained from three distinct groups of mice, alongside the percentage of the fibrotic area in each respective group. The decrease in collagen fibers after CBLB intervention indicates its potential anti-fibrotic effect in the context of AMI. Scale bars, 500 μm and 50 μm (enlarged views of boxed areas). All the data are expressed as mean ± SEM; *n* = 6 per group. * *P* < 0.05, ** *P* < 0.01 ns, no statistical significance

According to immunohistochemical analysis, mice in the C group had the highest levels of CBLB expression in their cardiac tissue (Fig. 3A). Compared to the A group, the B group exhibited obvious nucleolysis and consolidation of cardiomyocytes, as well as disorganized and broken myofibers, while the necrotic area of tissues and inflammatory cell infiltration were significantly reduced after CBLB intervention (Fig. 3B). MASSON staining revealed that collagen fibers were increased in the B group of mice, nevertheless collagen fibers were dramatically reduced after CBLB intervention (Fig. 3C).

### CBLB intervention alters the richness of the microbial communities after AMI

**Fig. 4:**
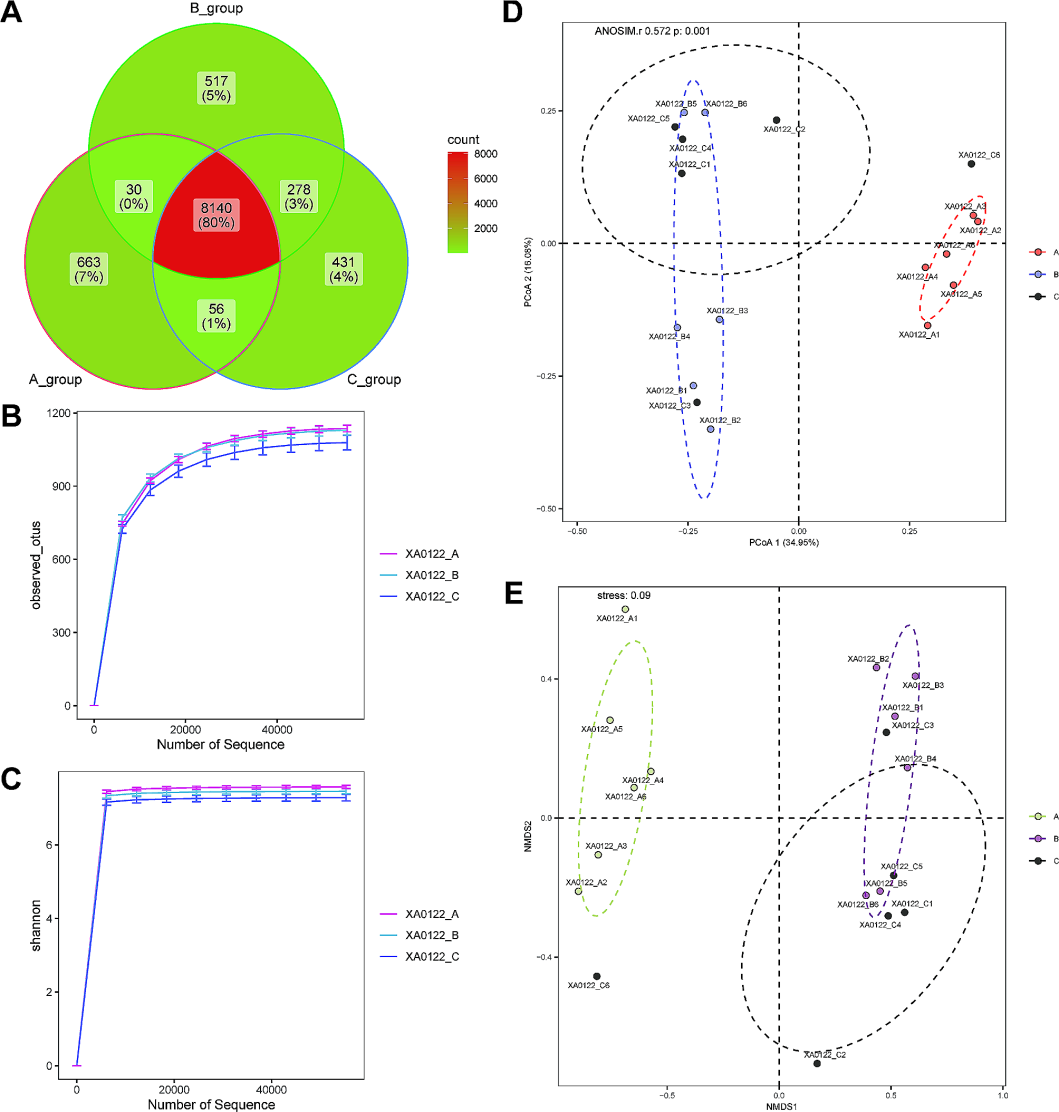
**Alpha and beta diversity in Sham, AMI, and AMI + CBLB overexpression mice groups A** Venn diagram of ASVs in the Sham, AMI, and AMI + CBLB overexpression mice groups. **B** and **C** The rarefaction curves for the Sham, AMI, and AMI + CBLB overexpression mouse groups were analyzed using the Alpha Diversity Index indicators: observed features **(B)** and Shannon **(C)**. **D** and **E** Beta diversity was calculated using brau_curtis by PCoA **(D)** and NMDS **(E)** analysis

To explore the changes in the intestinal microbiota of mice after CBLB intervention for AMI, we conducted 16 S rDNA sequencing of feces from each group. By analyzing ASVs in the three groups, we noticed that specifical ASVs were reduced after CBLB intervention compared to the A and B groups (Fig. 4A). The Alpha diversity rarefaction curves indicated a propensity for the microbiota diversity indices in group C mice to diminish relative to those in groups A and B **(**Fig. 4B and C**)**. We also estimated a number of alpha diversity metrics, although none of them reached levels of significance (**Supplementary Fig. 4**). Observation of the PCoA and NMDSS cluster plots (stress < 0.1) revealed that the three groups of samples were well differentiated from each other, while ANOSIM analysis showed *P* = 0.001, further supporting the results of Beta diversity (Fig. 4D and E, **Supplementary Table 2**). As a result, the findings demonstrate that AMI can alter the composition of intestinal microbiota in mice, and that CBLB intervention may affect the gut microbiota of AMI mice.

### Intergroup analysis of variance identifies five key microbiota

**Fig. 5:**
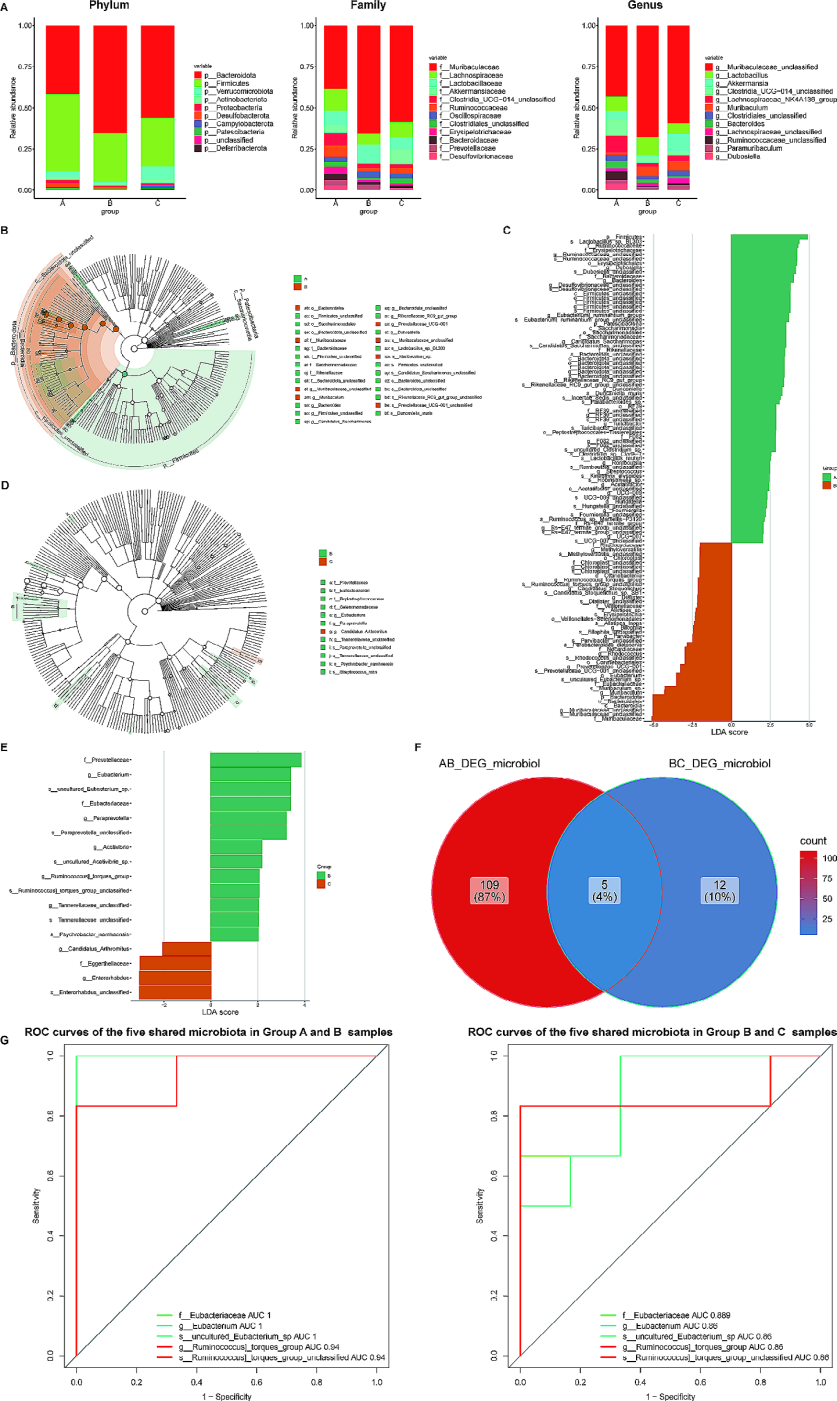
**Effects of CBLB intervention on the gut microbial composition and structure at different taxonomical levels. A** The flora composition of mice in the three groups at the phylum (left), family (middle), and genus (right) level. **B-E** Cladogram by LEfSe analysis (**B** and **D**) and LDA score bar graph (based on LEfSe analysis; **C** and **E**) in group A and B (**B** and **C**) and group B and C (**D** and **E**), which showed the microbial features most likely to explain the differences between classes. **F** Overlapping and shared microbiotas between group A-B and group B-C suggested by the Venn plot. **G** ROC curves evaluating the discriminative power of the five shared microbiota between Group A and B (left), and Group B and C (right)

To evaluate the changes in the composition of the intestinal microbiota after CBLB intervention in AMI mice, we investigated the composition and abundance of intestinal microbiota from different groups of mice at the phylum, family, and genus levels. At different hierarchical levels, we acquired three groups of mice with dominant phylum (Firmicutes and Bacteroidota), dominant families (Bacteroides, Lachnospiraceae, Lactobacillaceae, and Akkermansiaceae), and dominant genus (Muribaculaceae_unclassified, Lactobacillus, Akkermansia, Clostridia_UCG-014_unclassified and Muribaculum) (Fig. 5A**)**. Interestingly, Bacteroidota, Firmicutes, and Desulfobacterota had significant differences at the phylum level, with Bacteroidota rising and then falling in the three groups of A, B, and C, and Firmicutes and Desulfobacterota was opposite (**Supplementary Fig. 5A)**. Similarly, Muribaculaceae, Erysipelotrichaceae, Bacteroidaceae, and Desulfovibrionaceae differed considerably in the three sample groups at the family level (**Supplementary Fig. 5B)**. Muribaculaceae unclassified, Muribaculum, Bacteroides, Ruminococcaceae unclassified, and Dubosiella belonged to distinct genus across the three groups of samples (**Supplementary Fig. 5C)**.

The LEfSe evaluation revealed that in the B & A comparison group, the B and A groups obtained 43 differential microbiota (e.g., s Lactobacillus sp. BL303, g Romboutsia) and 71 (e.g., g Bilophila, s Alistipes_inops), respectively. In the C & B comparison groups, the C and B groups gained 4 and 13 differential microbiota, respectively, including g Eubacterium and g Paraprevotell, separately (Fig. 5B and E**)**. We then intersected the differential microbiota screened jointly by the two comparison groups to obtain five key microbiota (f Eubacteriaceae, g Eubacterium, s uncultured Eubacterium sp, g Ruminococcus] torques group, s Ruminococcus] torques group unclassified), and the AUC values under the ROC curves were greater than 0.86 in both comparison groups indicating that the key microbiota had a more accurate discriminatory ability (Fig. 5F and G**)**.

We examined the expression patterns of the aforementioned five key microbiota across the three mice groups **(Supplementary Fig. 6)**. The expression of f_Eubacteriaceae, g_Eubacterium, and s_uncultured_Eubacterium_sp exhibited significant differences among the groups (all *P* < 0.05). In the aftermath of AMI, these microbiotas registered a pronounced up-regulation (all *P* < 0.01) relative to group A. Post-CBLB intervention, their relative abundance was considerably reduced compared to group B mice (all *P* < 0.05), although it remained significantly elevated relative to group A mice (all *P* < 0.01). Both g_Ruminococcus_torques_group and s_Ruminococcus_torques_group_unclassified exhibited significant up-regulation post-AMI (both *P* < 0.05 relative to group A mice). Their relative abundance, following CBLB intervention, displayed an additional significant up-regulation (all *P* < 0.05 relative to group B mice). However, this additional up-regulation did not present a statistically significant difference when compared to Group A mice.

### Eleven key metabolites were extracted following the comparison of the three groups

**Fig. 6:**
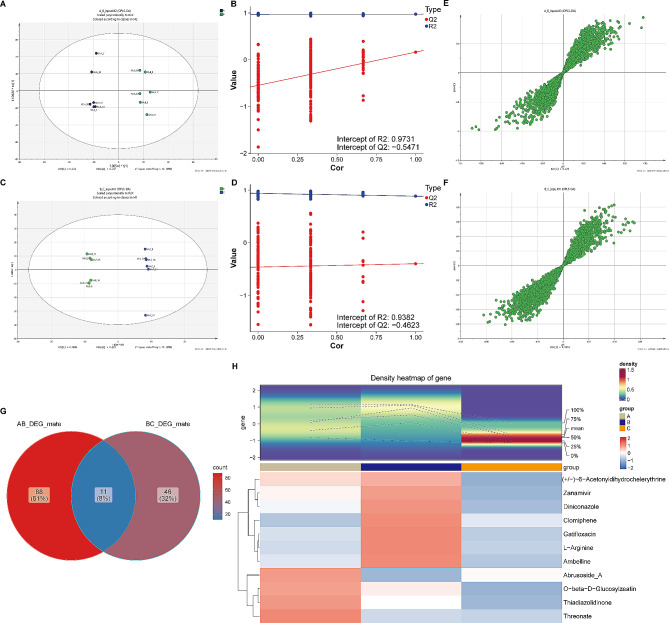
**Chemometric analysis of metabolites and identification of key metabolites A** and **C** OPLS-DA score plots for Group B vs. A (**A**) and Group C vs. B (**C**). **B** and **D** Cross-validation results of OPLS-DA model for Group B vs. A (**B**) and Group C vs. B (**D**). **E** and **F** OPLS-DA S-plot of LC-MS data for Group A&B samples (**E**) and Group B&C samples (**F**), respectively. **G** Overlapping and shared metabolites between Group B vs. A and Group C vs. B suggested by the Venn plot. **H** Heatmap displaying expression patterns of 11 key metabolites across three groups

To explore the altered metabolite regulation in mice after AMI and CBLB interventions, we conducted non-targeted metabolite sequencing of mice serum. The OPLS-DA plots revealed a distinct difference between the samples in each group (R2 was higher than Q2 in both comparison groups, with Q2 absolute values greater than 0.5 in the B & A and greater than 0.4 in the B and C groups) (Fig. 6A and D**)**. Following the establishment of S plots in accordance with OPLS-DA predictions, 99 differential metabolites (58 raised and 41 reduced) were found in the comparison groups for B and A, and 57 differential metabolites (10 raised and 47 reduced) in the comparison groups for B and C (Fig. 6E and F, **Supplementary Tables 3 and 4)**. By intersecting the differential metabolites identified in the two comparison groups, eleven key metabolites (e.g. Threonate and Thiadiazolidinone) were located (Fig. 6G and H, **Supplementary Table 5)**.

**Supplementary Fig. 7** illustrated the expression trends of the 11 key metabolites across the three groups. Threonate was the only metabolite that demonstrated a consistent upward trend in expression (*P* < 0.05 for both B vs. A and C vs. B), despite the lack of a statistically significant sustained increase in group C relative to group A. In comparison to group A mice, Thiadiazolidinone, Zanamivir, Acetonyldihydrochelerythrine, and Abrusoside_A exhibited a significant trend of down-regulation after AMI (all *P* < 0.05), while Diniconazole, Ambelline Gatifloxacin, O_beta_D_Glucosylzeatin, Clomiphene, and L_Arginine showed significant up-regulation (all *P* < 0.05). Conversely, the aforementioned four metabolites that were downregulated post-AMI experienced an upregulation due to CBLB intervention (all *P* < 0.05), while CBLC intervention resulted in a significant downregulation of the six metabolites that were initially upregulated post-AMI (all *P* < 0.05). Similar to Threonate, the expression levels of these 10 key metabolites did not show significant differences post-CBLB intervention when compared to group A mice.

### Selection and functional exploration of differentially expressed genes in distinct comparative groups

**Fig. 7:**
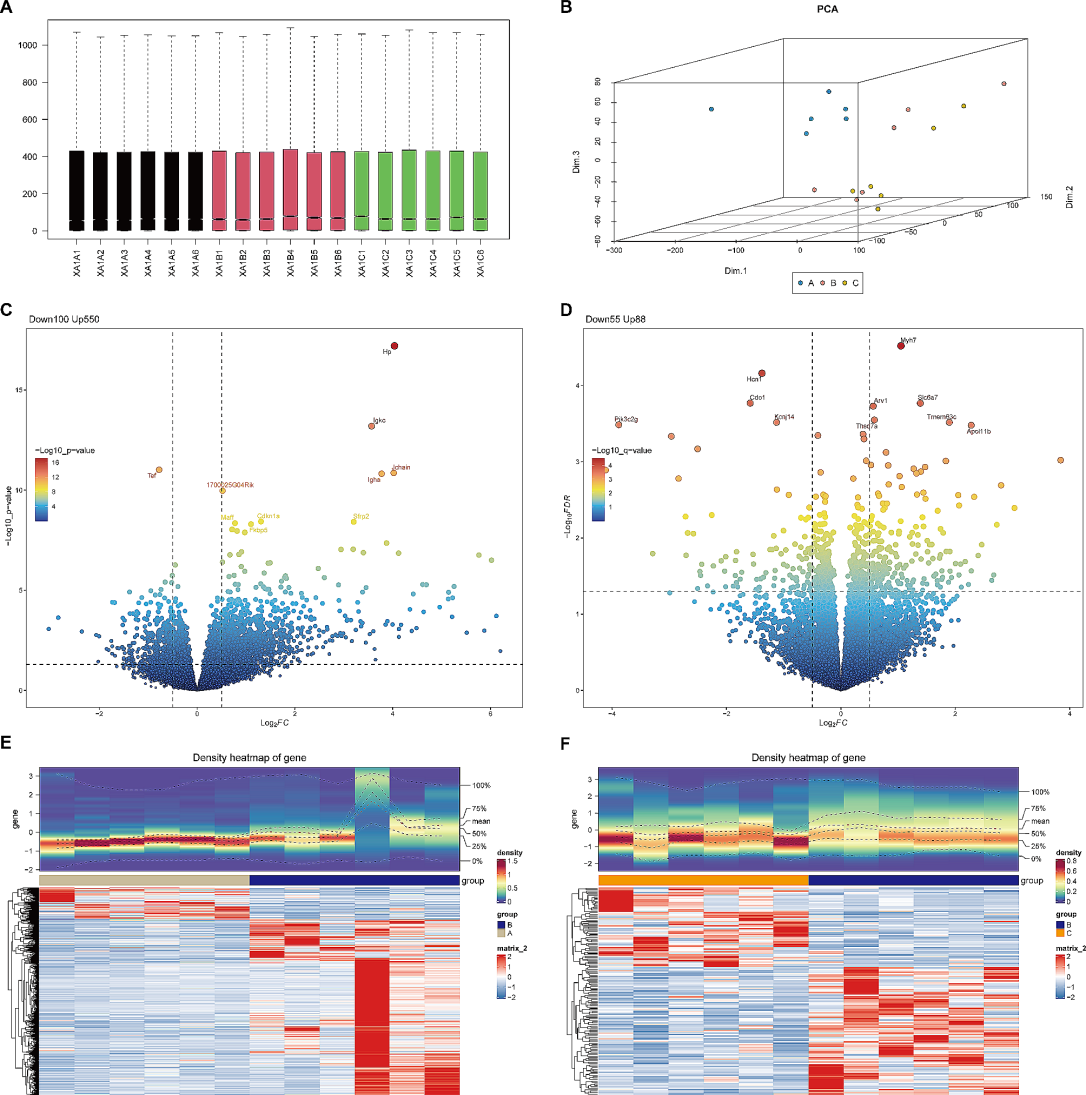
**Identification of DEGs. A** Boxplot of gene expression in 3 groups of mouse samples normalized by DESeq2 software. **B** 3D PCA was performed based on transcriptome data from 3 groups of mice. **C** and **D** The volcano plot of the DEGs was screened in the Group B vs. A (**C**) and Group C vs. B (**D**). **E** and **F** Heatmap displaying expression patterns of DEGs1 (Group B vs. A; **E**) and DEGs2 (Group B vs. C; **F**)

We employed RNA-seq to investigate the differences in cardiac tissue regulation between the three groups of mice at the gene level. Then, we discovered that the samples were all at the same level of expression after pre-processing the data, and the PCA analysis also revealed that there were significant differences between the three sets of samples for subsequent analyses (Fig. 7A and B**)**. In the B and A and B and C comparator groups, we detected 650 differently expressed genes (DEGs1, 550 enhanced and 100 reduced) and 143 differentially expressed genes (DEGs2, 88 enhanced and 55 reduced) (Fig. 7C and F**)**.

**Fig. 8:**
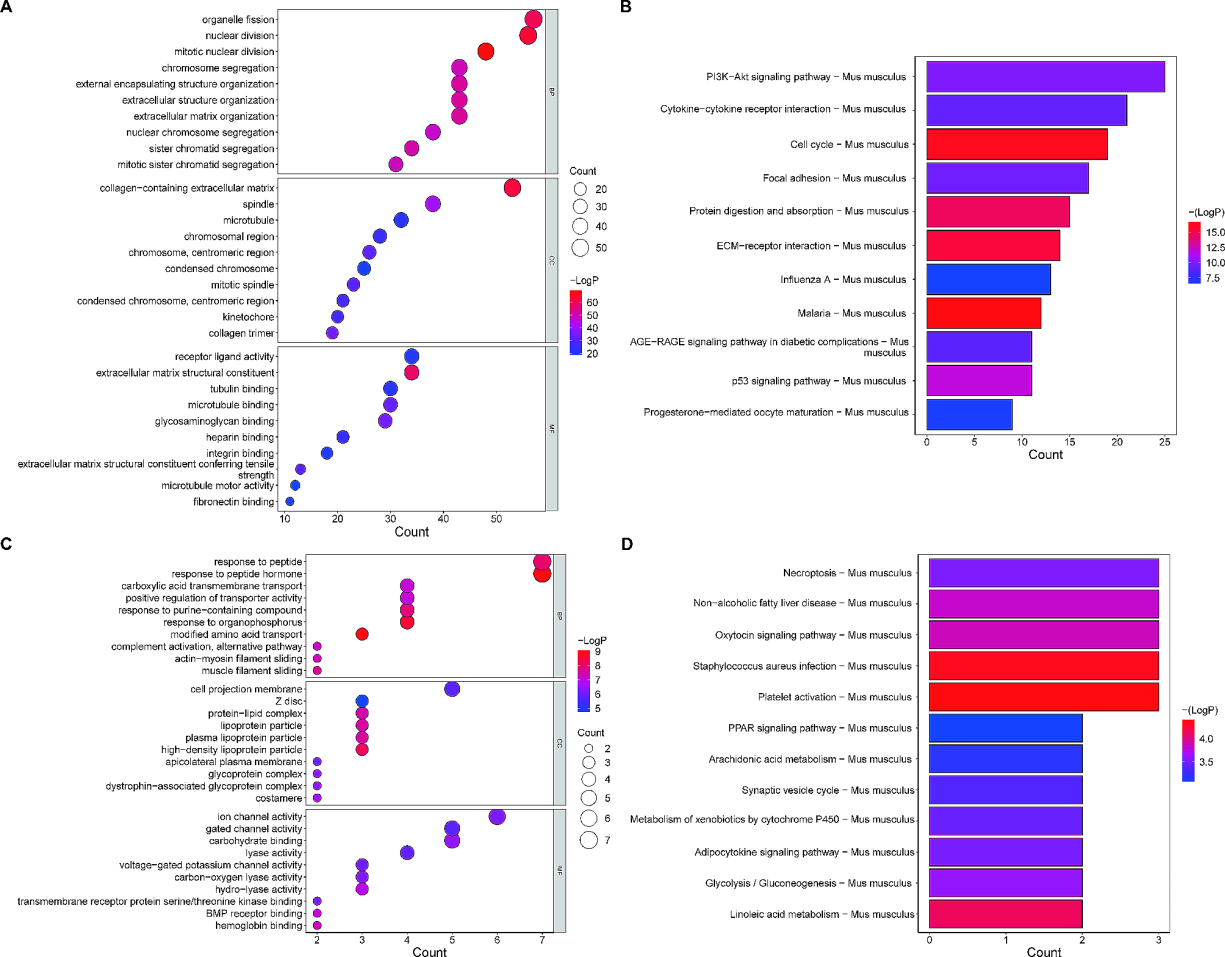
**GO and KEGG pathway enrichment analyses of the DEGs. A** and **C** GO terms involved by DEGs1 (Group B vs. A; **A**) and DEGs2 (Group C vs. B; **C**) in BP, CC, and MF categories. Dot sizes represent counts of enriched genes, and dot colors represent the -LogP value. **B** and **D** KEGG pathway enrichment analysis of DEGs1 (**B**) and DEGs2 (**D**)

DEGs1 functional enrichment revealed that it is mostly involved in organelle fission, collagen-containing extracellular matrix, and the PI3K-Akt signaling pathway, as well as cytokine-cytokine receptor interactions (Fig. 8A and B**)**. Functional enrichment on the basis of DEGs2 has revealed that it is primarily associated with response to peptide, response to peptide hormone, and Staphylococcus aureus infection, Platelet activation (Fig. 8C and D**)**.

### Selection of hub genes

**Fig. 9:**
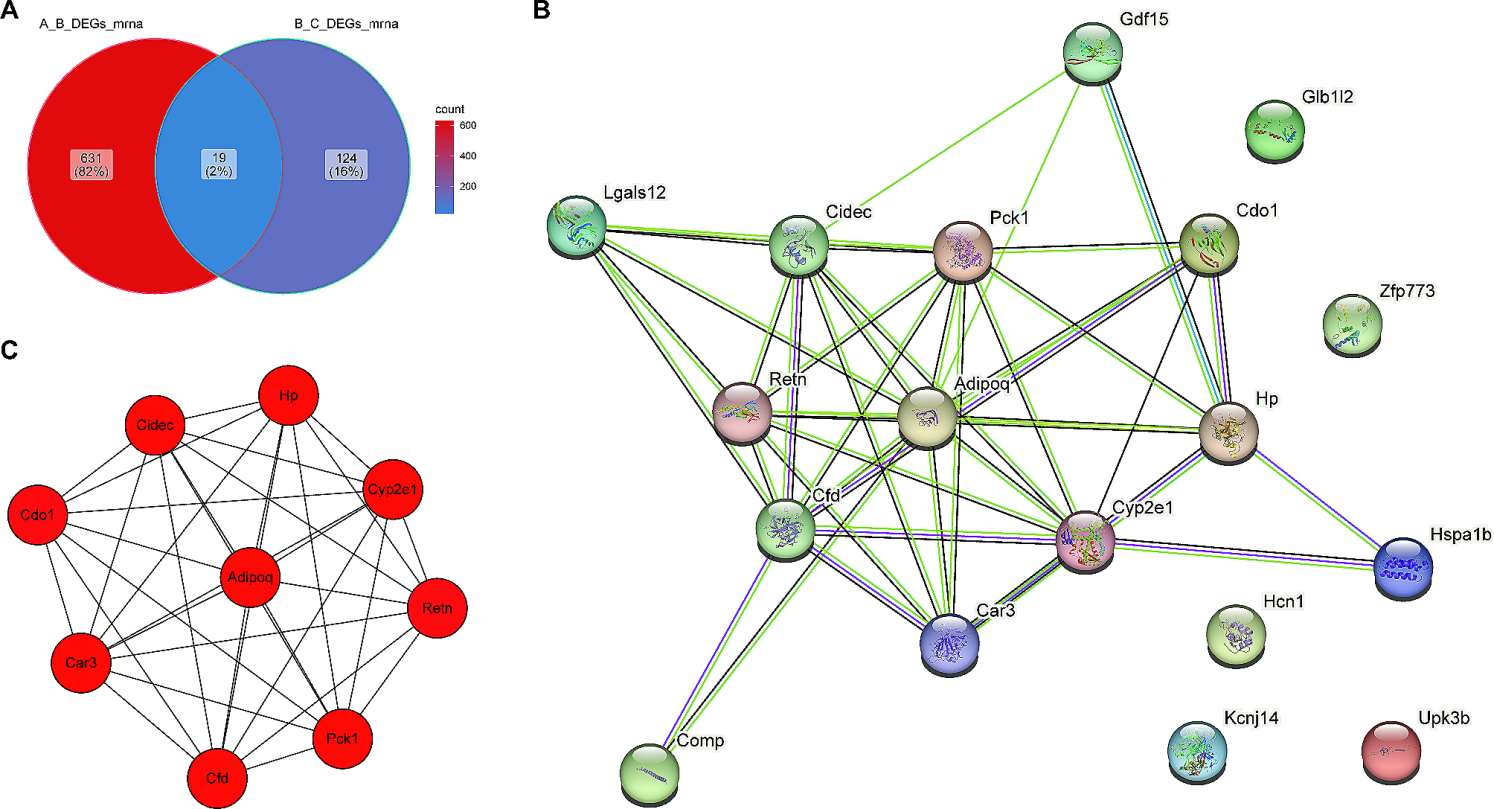
**The PPI network analyzed by STRING and MCODE. A** Overlapping and shared genes between DEGs1 (Group B vs. A) and DEGs2 (Group C vs. B) suggested by the Venn plot. **B** PPI network for the 19 key genes. **C** A key cluster with 9 genes was further chosen as hub genes by MCODE.

By intersecting DEGs1 and DEGs2, we were able to further identify the 19 key genes of CBLB-regulated AMI (Fig. 9A, **Supplementary Table 6)**. PPI investigation into key genes revealed that they have interactions between them, including Cfd-Comp and HP and Hspa1b (Fig. 9B**)**. Besides, nine hub genes (*Cidec*, *Hp*, *Car3*, *Retn*, *Pck1*, *Cfd*, *Adipoq*, *Cdo1* and *Cyp2e1*) were tested employing the MCODE method based on the results mentioned above (Fig. 9C**)**.

In comparison to group A mice, all eight hub genes exhibited a substantial increment in their expression post-AMI (all *P* < 0.05), aside from Car3 whose expression demonstrated an inclining trend but lacked statistical significance. Conversely, post-CBLB intervention, the expression levels of all hub genes were significantly suppressed relative to group B (all *P* < 0.05), and these levels were indistinguishable from those in group A. The comparative expression profiles of the nine hub genes across the three groups can be viewed in **Supplementary Fig. 8**.

### Multi-omics correlation analysis

**Fig. 10:**
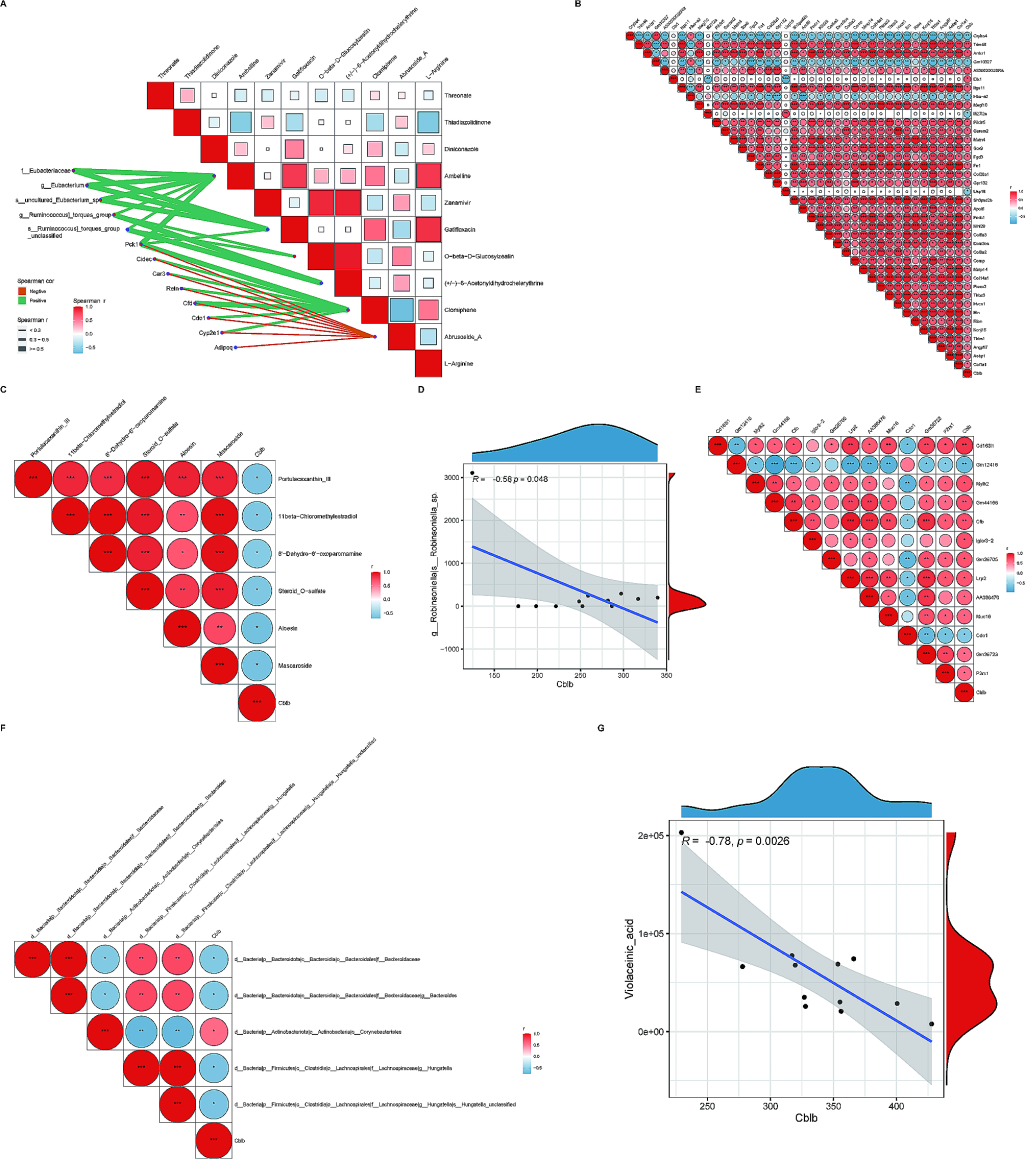
**Results of multi-omics correlation analysis A** Correlation graph of key microbiota-key genes-key metabolites. The triangle graph represents the correlation graph of 11 key metabolites. The connecting line presented in orange representing negative correlation; a green line representing positive correlation. The thickness of the line represents Spearman’s correlation coefficient “r”. **B** Heatmap of the correlation between CBLB and 39 DEGs (from B vs. A). **C** Heatmap of the correlation between CBLB and 6 differential metabolites (from B vs. A). **D** The correlation analysis of CBLB and g_Robinsoniella|s_Robinsoniella_sp (from B vs. A). **E** Heatmap of the correlation between CBLB and 13 DEGs (from C vs. B). **F** Heatmap of the correlation between CBLB and 5 differential metabolites (from C vs. B). **G** The correlation analysis of CBLB and Violaceinic_acid (from C vs. B). The correlation heatmaps exclusively visualize pairs of relationships that meet the criteria of |*r* > 0.3| and *P* < 0.05

To further elucidate the regulatory relationships between gut microbiota, metabolites and genes in mice after AMI and CBLB intervention, we examined the correlation analysis of 11 key metabolites, 9 hub genes, and 5 key microbiota (Fig. 10A**)**. Correlation research revealed that Ambelline, Gatifloxacin, and O-beta-D-Glucosylzeatin were positively correlated with five key microbiota, Clomiphene had a positive association with nine hub genes, and Abrusoside A was significantly adversely correlated with nine hub genes. Ultimately, in order to focus on CBLB’s role, we assessed the association of CBLB with DEGs, microbiota, and metabolites independently. The findings revealed that CBLB was strongly linked with 39 (B and A) and 13 (B and C) DEGs, 6 (B and A) and 1 (B and C) differential metabolites, and 1 (B and A) and 5 (B and C) differential microbiota, respectively (Fig. 10B and G**)**.

## Discussion

In this study, our focus was to investigate the functional role and potential regulatory mechanisms of CBLB in the AMI mouse model. To achieve this, we employed a distinctive combination of transcriptome, 16 S microbial sequencing, and untargeted metabolomics sequencing data. To the best of our knowledge, no previous studies have explored the role of CBLB in AMI using this unique multi-omics approach. Our findings suggest that overexpression of CBLB significantly lessens myocardial infarction in AMI mice, modifies gut microbial abundance, and influences the expression of certain metabolites and genes.

Our initial observations reveal that overexpression of CBLB significantly curtailed the degree of myocardial infarction in AMI mice, which is consistent with our hypothesis. This observation aligns with the research by Hongjun You et al. [9], which identifies CBLB as an essential immune-inflammation-related biomarker of AMI, reporting a significant decrease in CBLB levels in AMI patients. Both findings mutually reinforce the role of CBLB in AMI, underscoring its significance. Secondly, we discovered that myocardial infarction could influence body weight gain in mice, an innovative observation that offers fresh insights into the systemic effects of myocardial infarction. The precise causes of weight loss, as well as the correlation between weight fluctuations and myocardial infarction severity, warrant further investigation. Moreover, our data indicated that CBLB intervention markedly reduced the proliferation of collagen fibers, suggesting potential preventive and therapeutic roles of CBLB in myocardial fibrosis, a prevalent complication post-myocardial infarction impacting heart’s systolic and diastolic functions [21–23]. This novel insight broadens our comprehension of myocardial infarction treatment. Nonetheless, a more in-depth investigation into the specific mechanisms driving these outcomes is necessary.

Furthermore, our research further illustrates that CBLB intervention significantly alters the composition of gut microbiota in AMI mice. This discovery underscores the critical role of microbiota in AMI development and insinuates that CBLB might exert a protective effect against AMI through gut microbiota modulation. Among the five key microbiota identified, f_Eubacteriaceae, g_Eubacterium, s_uncultured_Eubacterium_sp, g_[Ruminococcus]_torques_group, and s_[Ruminococcus]_torques_group_unclassified may play a significant role in cardiovascular disease. Eubacteriaceae, a gastrointestinal flora, encompasses several genera, one of which is Eubacterium. Evidence suggests that alterations in gut microbiota composition, such as Eubacterium, might influence fecal and plasma metabolite levels, including trimethylamine N-oxide (TMAO) [24]. Notably, studies have demonstrated that TMAO can induce atherosclerosis and is associated with an elevated cardiovascular risk [25, 26]. Building upon these findings, our study explores the potential impact of CBLB on gut microbiota composition, specifically in the context of AMI. We do not conclude whether these changes are beneficial or detrimental, but we provide a novel perspective on the study of gut microbiota in cardiovascular disease. Research into the relationship between gut flora, such as Eubacteriaceae and Eubacterium, and cardiovascular disease, as well as their specific mechanisms and impact, is still nascent and warrants further exploration. Additionally, Ruminococcus torques, an anaerobic bacterium, is prevalent in the human gut and falls under the genus Ruminococcus [27]. The Ruminococcus torques group can produce short-chain fatty acids, known to enhance cardiac function and suppress inflammatory responses [28]. Consequently, aberrations in the [Ruminococcus]_torques_group might be implicated in the initiation and progression of AMI. To our knowledge, this is the first study that explores the role of the [Ruminococcus]_torques_group in AMI, providing fresh insights into potential therapeutic targets. Our work further contributes to the existing literature by suggesting that specific gut microbiota could be manipulated to mitigate the progression of AMI, a novel concept that warrants further investigation.

We detected altered metabolites in mouse serum post-AMI and CBLB interventions. Through cross-comparison of the differential metabolites from B with A and B with C, we pinpointed 11 key metabolites. Threonate and L-Arginine were particularly noteworthy. Previous studies indicated threonic acid as a highly relevant cecal metabolite for AMI [29], a finding that aligns with our results. This new evidence strengthens the existing literature by providing additional support for the relevance of threonic acid in the context of AMI. Concurrently, a study by Jie Cao et al. [30] employed seven differential metabolites, inclusive of L-threonic acid, to devise a multilayer perceptron (MLP) model. This model predicts AMI type II in autopsy cases of sudden cardiac deaths with 88.23% accuracy and an ROC value of 0.89. Our findings build upon this work by suggesting a potential expansion of the metabolite set used in this predictive model, thereby potentially improving its predictive accuracy. L-Arginine, an alkaline endogenous amino acid, exhibits cardioprotective properties and is pivotal for endothelial function, driving vasodilation, reducing inflammation, and enhancing physical functionality [31–33]. The inclusion of L-Arginine in our list of key metabolites underscores its importance in AMI and provides a potential avenue for therapeutic intervention. We also detected the presence of two drugs, Gatifloxacin [34] and Zanamivir [35], in the metabolites. These drugs, commonly used for treating bacterial and influenza virus infections respectively, might indicate potential infectious complications post-AMI. This observation extends existing knowledge by suggesting a potential link between AMI and infectious disease, which merits further investigation. Our metabolite list also includes Diniconazole, a fungicide [36], and Clomiphene, a gonadotropin [37]. Nevertheless, their roles in heart disease remain unclear and necessitate further investigation. The identification of these compounds in our study adds a novel dimension to the metabolomic profile of AMI and could potentially open up new research directions. Finally, the functions and roles of Thiadiazolidinone, Ambelline, O-beta-D-Glucosylzeatin, (+/-)-6-Acetonyldihydrochelerythrine, and Abrusoside_A remain unclear, necessitating further exploration in AMI and heart disease context. The inclusion of these metabolites in our study expands the current understanding of the metabolic changes associated with AMI, and further investigation could potentially reveal novel biomarkers or therapeutic targets.

We have identified nine pivotal genes that potentially contribute to CBLB regulation in AMI. The identified genes are: cell death inducing DFFA like effector c (Cidec), Haptoglobin (Hp), Carbonic Anhydrase 3 (Car3), Resistin (Retn), Phosphoenolpyruvate Carboxykinase 1 (Pck1), Complement Factor D (Cfd), Adiponectin, C1Q And Collagen Domain Containing (Adipoq), Cysteine Dioxygenase Type 1 (Cdo1), and Cytochrome P450 Family 2 Subfamily E Member 1 (Cyp2e1). The elucidation of these genes strengthens the etiological basis of AMI and offers fresh perspectives on AMI pathogenesis. The novelty of our findings lies in the identification of these specific genes and their potential contributions to AMI. Although some of these identified genes have been previously linked to various diseases and metabolic pathways, their direct involvement in AMI pathogenesis has not been fully elucidated, thus expanding the existing literature and providing new insights. Cidec encodes a protein that plays a role in lipid metabolism, promoting lipid droplet formation in adipocytes and possibly mediating adipocyte apoptosis [38]. The associated metabolic pathways involve plasma lipoprotein assembly, remodeling, clearance, and metabolism [39]. Proteins encoded by Hp are instrumental in acute phase and inflammatory responses, with specific phenotypes linked to numerous common diseases, including cardiovascular and autoimmune diseases, and malignant tumors [40, 41]. Car3 belongs to a multigene family that encodes carbonic anhydrase isozymes. Research indicates that Car2 and Car3 might be risk factors and potential biomarkers for diagnosing dilated cardiomyopathy in patients with atrial fibrillation [42]. Retn, a member of the resistin family of hormones, is linked with insulin resistance [43] and is expressed in cardiac tissues. It plays a part in the development of right ventricular dysfunction and adverse remodeling via its immunomodulatory activity [44]. Pck1, a significant glycoheterotrophic enzyme found in the liver and kidney, has been linked to liver and kidney diseases [45]. Studies suggest that Pck1 is a key gene involved in the mechanisms of curcumin’s effects on atrial fibrillation [46]. Cfd, a component of the complement system, is associated with immune responses [47]. The Adipoq gene, exclusively expressed in adipose tissue, encodes proteins that circulate in the plasma and participate in metabolic and hormonal secretory processes [48, 49]. Research has shown that the Adipoq gene might serve as a risk factor for cardiovascular complications in type 1 diabetes mellitus [50]. Cdo1, a protein-coding gene, is associated with anti-inflammatory characteristics that impact systemic metabolic traits [51, 52]. Its role in cardiac diseases remains unclear. Cyp2e1, an enzyme participating in the metabolism of various drugs and toxins, also plays a role in fatty acid metabolism [53, 54]. Present evidence suggests an increase in CYP2E1 levels in human ischemic and dilated hearts [55] and in left ventricular tissue from spontaneously hypertensive rats [56], consistent with our findings. Hence, our study builds upon the existing body of literature by providing a comprehensive analysis of these genes in relation to AMI, paving the way for future research and potential therapeutic interventions.

Despite not yet completely unraveling the specific mechanism of CBLB in AMI regulation, it remains a limitation of our current study. However, Gene Ontology (GO) enrichment analysis of the 143 DEGs between group B and group C **(Supplementary Table 7)** has paved the way for fresh insights and future mechanistic studies. Analysis of GO-BP items reveals a close relationship between these DEGs and factors such as ventricular myocardial tissue, ventricle, cardiac morphogenesis, ventricular muscle tissue development, and adult heart development. Concurrently, significant enrichment was also observed in the ‘negative regulation of vascular endothelial growth factor receptor signaling pathway’ and ‘coronary artery morphogenesis’. Importantly, the involvement of immunology-related biological processes, including the ‘immunological memory process’, ‘natural killer cell proliferation’, and ‘humoral immune response’, is also observed. Consequently, we hypothesize that CBLB intervention might effectively attenuate the augmentation of collagen fibers in group C mice by modulating the signaling pathways associated with these biological processes. In subsequent research, we aim to delve deeper into the exact mechanism of CBLB and explore its potential therapeutic application in treating AMI.

In our study, we discovered intriguing correlations involving gut flora, metabolites, and genes. We noted that the expression trends of certain metabolites (e.g., Thiadiazolidinone, Zanamivir, Abrusoside_A) were inversely proportional to the expression trends of key genes. These observations intimate that metabolites might influence AMI pathogenesis by modulating the expression of pivotal genes. This discovery expands upon existing literature, as it provides new insights into the interconnectedness of gut flora, metabolites, and genes in the context of AMI. The identification of this inverse relationship introduces potential new mechanisms in AMI pathogenesis. As such, our findings not only deepen our understanding of AMI, but also lay the groundwork for potential advancements in its treatment strategies.

Our findings suggest potential clinical implications. If the molecular changes associated with CBLB’s regulation of AMI are validated in further studies, they could provide novel biomarkers for AMI diagnosis and prognosis. Moreover, understanding these mechanisms could lead to new therapeutic strategies, such as targeting CBLB or its associated pathways to modulate AMI progression.

However, we acknowledge that our study possesses certain potential limitations. Firstly, our selection of samples may have introduced some bias into our research. To mitigate this, we employed a rigorous selection process, ensuring our samples were representative of the population in terms of age, sex, and other pertinent variables. Moreover, the bioinformatics tools and databases used in our study might have inherent biases.

Although our experimental design was robust and aimed at eliminating potential sources of bias, we acknowledge that it may not be completely devoid of bias. We carried out multiple experimental replicates and employed appropriate statistical tests to mitigate the impact of any potential bias. Despite our concerted efforts, there might have been some biases in data collection.

While our statistical models provide valuable insights, it’s important to acknowledge potential limitations. The used parametric tests and Spearman correlation analysis assume certain characteristics of the data which may not always hold true in biological studies. Also, despite multiple testing corrections, the risk of false positives remains due to the high-dimensional nature of the data. Additionally, in silico methods for data interpretation rely on existing databases, potentially missing novel interactions or pathways. We recommend cautious interpretation of the results and further validation through additional statistical methods or experimental confirmation. This study primarily investigates the regulatory mechanisms of CBLB in AMI using a mouse model. However, this model does not accurately represent the human physiological environment. Thus, it is necessary to validate our findings in larger samples and diverse models. In terms of the specificity of the identified metabolites and genes, it should be noted that while they exhibited significant statistical correlation with AMI in our study, they may not be exclusive to AMI. Other diseases or physiological conditions may also influence their levels, which could introduce confounding factors. Furthermore, the role of these metabolites and genes in AMI is not completely understood. While we have presented correlative findings, causal relationships and mechanisms of action need to be confirmed through further studies. In addition to the systems biology strategies highlighted, we will also explore the effects of CBLB in different contexts of cardiovascular disease. This would not only validate our current findings but also open new avenues for understanding the role of CBLB in the broader spectrum of cardiovascular diseases. Furthermore, we also plan to investigate the role of CBLB in human patients to gain a real-world perspective of its implications. Lastly, we recognize the potential of integrating emerging technologies or computational approaches in our research, which we believe could provide exciting perspectives on future research avenues, and thus, we will consider incorporating these methods in our subsequent studies.

## Conclusions

In conclusion, this study elucidates potential regulatory mechanisms of CBLB in AMI, identifying key microorganisms, metabolites, and genes. These findings significantly enrich our understanding of AMI’s etiology and provide fresh insights into the underlying molecular processes of AMI. Furthermore, our study lays a crucial theoretical foundation for the potential therapeutic application of CBLB in AMI treatment.

The implications of our findings are multifold. From a basic research perspective, they open up new avenues for understanding the pathophysiology of AMI at a molecular level. From a translational perspective, the identified molecules may serve as potential therapeutic targets, setting the stage for the development of novel AMI treatment strategies.

However, the transition from these findings to clinical application presents significant challenges that we must address. One potential barrier is the translation from animal models to human trials. To overcome this, comprehensive pre-clinical testing on multiple animal models followed by carefully designed phase I trials in humans is necessary. This will not only establish the safety and efficacy of therapeutic agents targeting CBLB but also help identify potential side effects.

Another challenge lies in the variability of patient responses due to genetic and environmental factors. Personalized medicine approaches, involving genomic profiling of patients, may be necessary to ensure the optimal efficacy of therapies targeting CBLB. Additionally, the cost-effectiveness of these novel treatments should also be evaluated to ensure their accessibility to all patients.

While our findings significantly advance the current state of knowledge and bridge the gap between basic research and clinical application, it is crucial to note that further validation and exploration are required. Future studies should focus on addressing these challenges to facilitate the transition of our research findings into therapeutic application. Such studies will undoubtedly bring us closer to our ultimate goal of improving AMI management and patient outcomes.

### Electronic supplementary material

Below is the link to the electronic supplementary material.

Supplementary Material 1

Supplementary Material 2

Supplementary Material 3

Supplementary Material 4

Supplementary Material 5

Supplementary Material 6

Supplementary Material 7

Supplementary Material 8

Supplementary Material 9

Supplementary Material 10

Supplementary Material 11

Supplementary Material 12

Supplementary Material 13

Supplementary Material 14

Supplementary Material 15

Supplementary Material 16

Supplementary Material 17

## Data Availability

All raw data supporting the conclusions of our study, as well as any relevant code, will be made available to any researcher for the purpose of reproducing the results or replicating the procedures, upon reasonable request to the corresponding author.

## Abbreviations

AMI: acute myocardial infarction
CBLB: Casitas B lymphoma-b
DEGs: differentially expressed genes
PPI: protein-protein interaction
293T cells: Human embryonic kidney cells
TTC: 2, 3, 5-triphenyltetrazolium chloride
HE: Hematoxylin and Eosin
ASVs: Amplicon Sequence Variants
PCoA: principal coordinates analysis
NMDS: Non-metric multidimensional scaling
ANOSIM: analysis of similarity
LEfSe: linear discriminant analysis effect size
LDA: linear discriminant analysis
ROC: receiver operating characteristic
OPLS-DA: orthogonal partial least discriminant analysis
LC-MS: liquid chromatography mass spectrometry
GO: Gene ontology
KEGG: Kyoto Encyclopedia of Genes and Genomes
STRING: Search Tool for the Retrieval of Interacting Genes
MCODE: Minimal Common Oncology Data Elements
