# Supplementary figures and images for: Exploring the role of CBLB in acute myocardial infarction: transcriptomic, microbiomic, and metabolomic analyses
Source: J Transl Med. 2024 Jul 14;22:654. doi: 10.1186/s12967-024-05425-y (PMC11247792; doi:10.1186/s12967-024-05425-y)

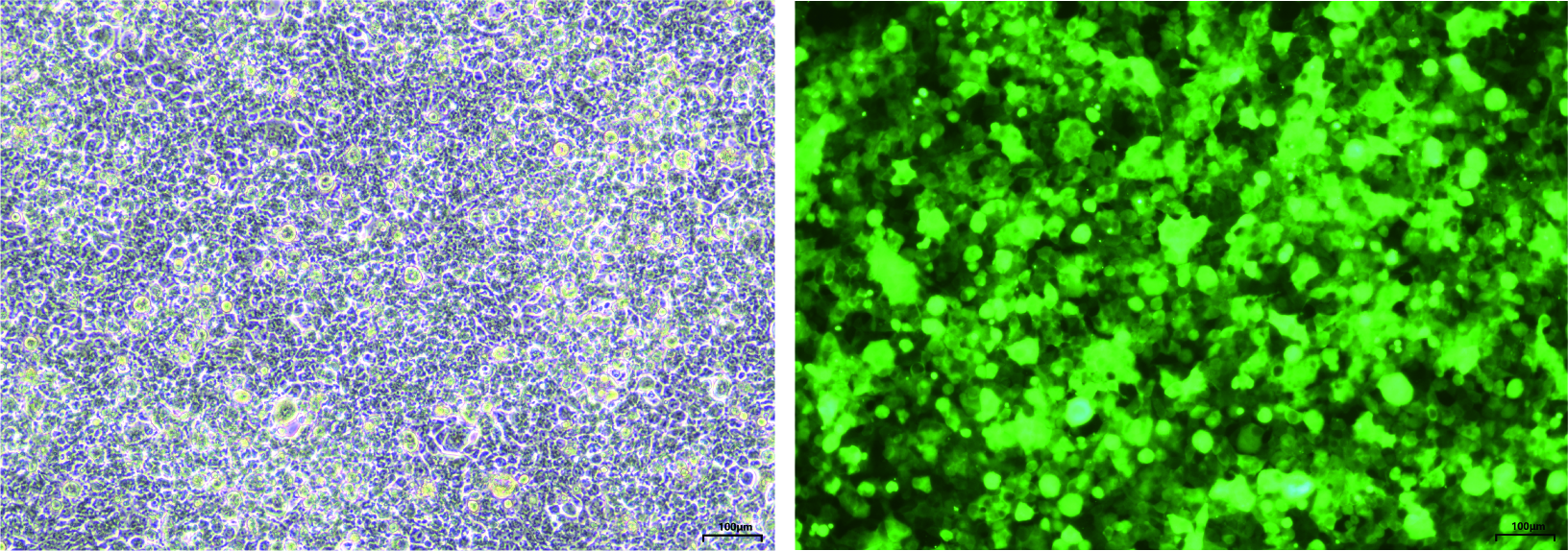

Supplement: Supplementary file 1 — Supplementary Material 1 [file 12967_2024_5425_MOESM1_ESM.tif]

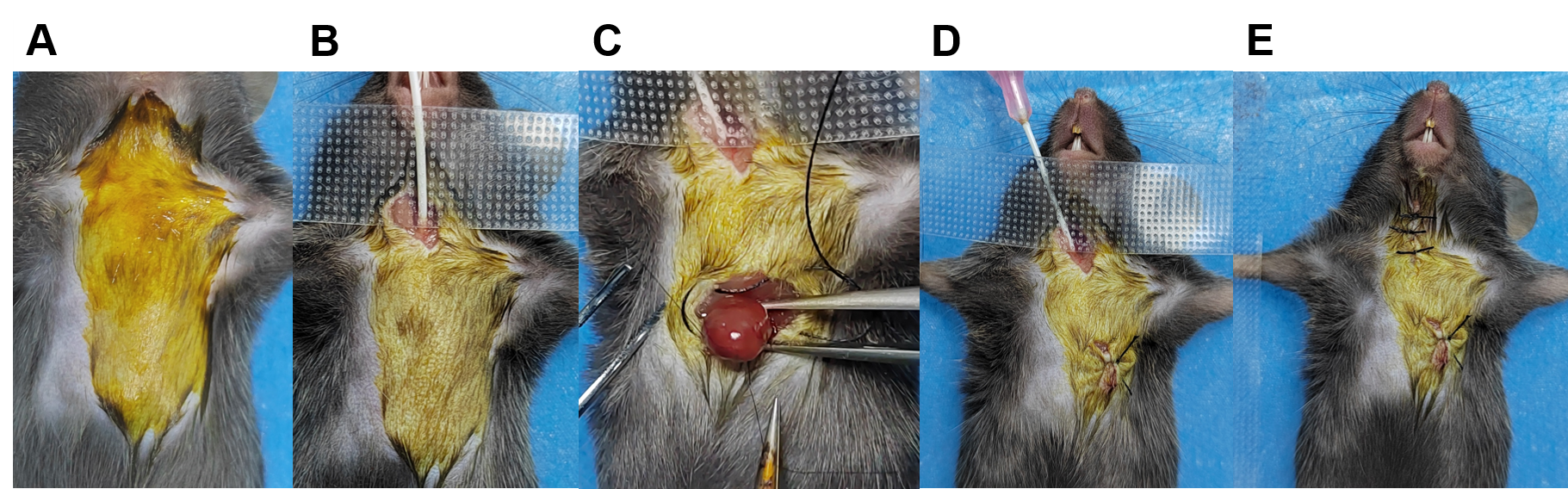

Supplement: Supplementary file 2 — Supplementary Material 2 [file 12967_2024_5425_MOESM2_ESM.tif]

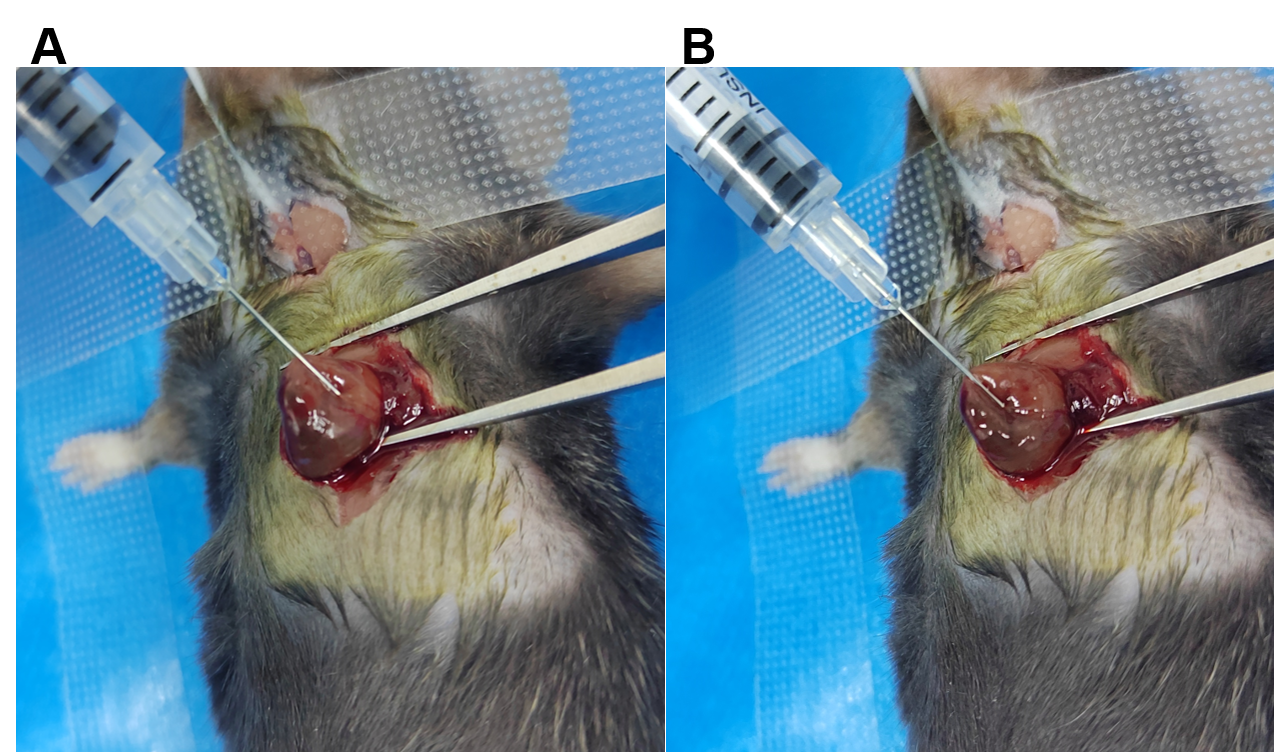

Supplement: Supplementary file 3 — Supplementary Material 3 [file 12967_2024_5425_MOESM3_ESM.tif]

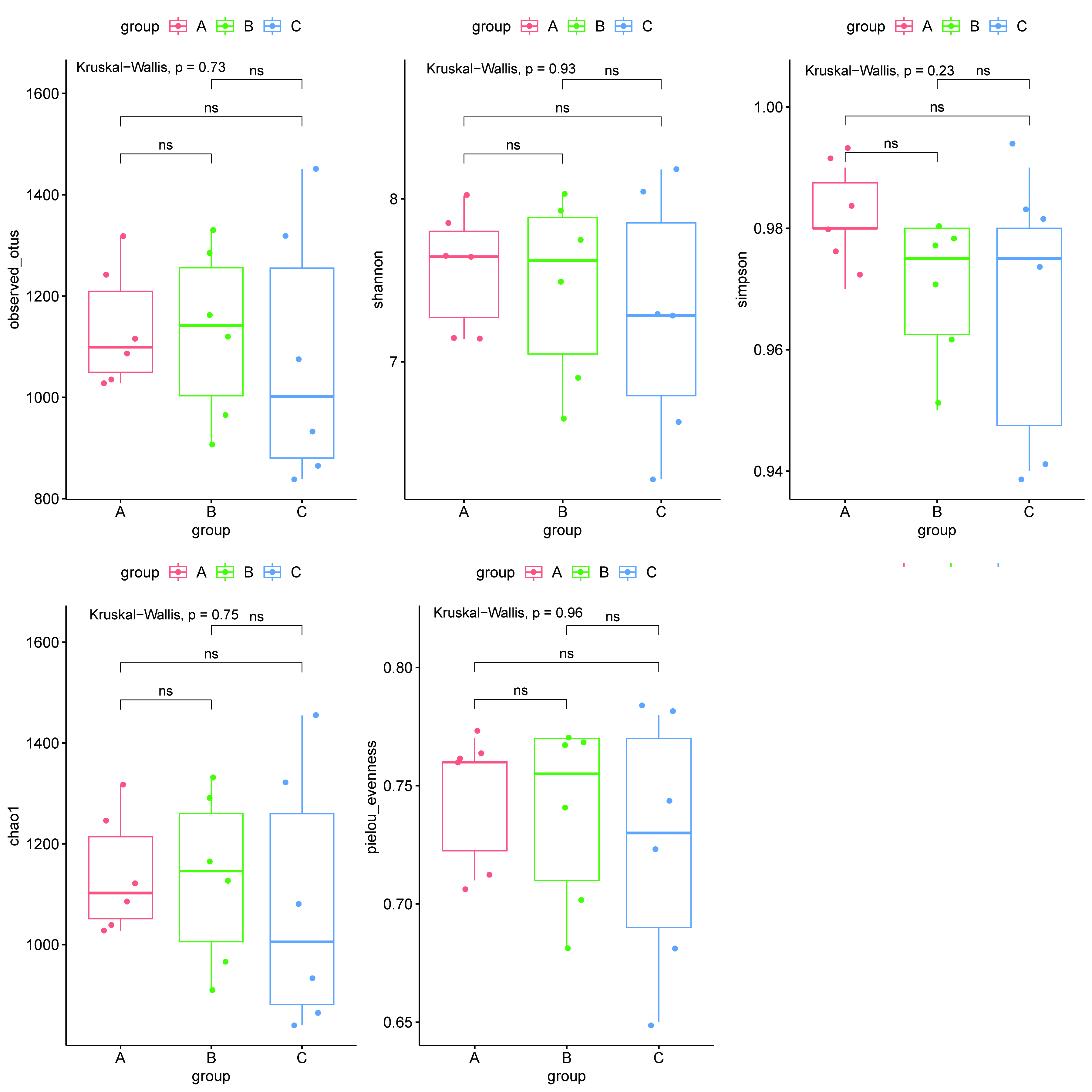

Supplement: Supplementary file 4 — Supplementary Material 4 [file 12967_2024_5425_MOESM4_ESM.tif]

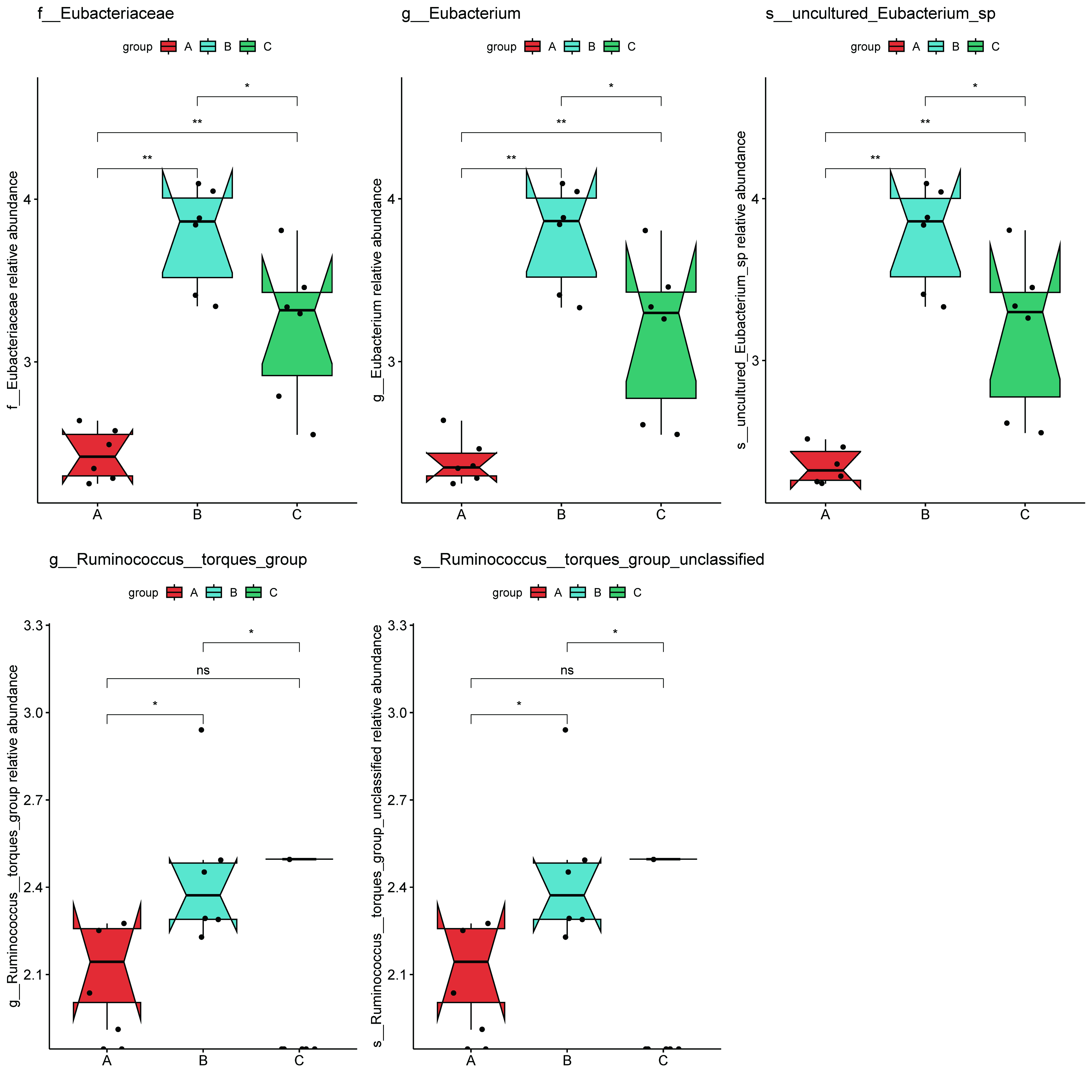

Supplement: Supplementary file 6 — Supplementary Material 6 [file 12967_2024_5425_MOESM6_ESM.tif]

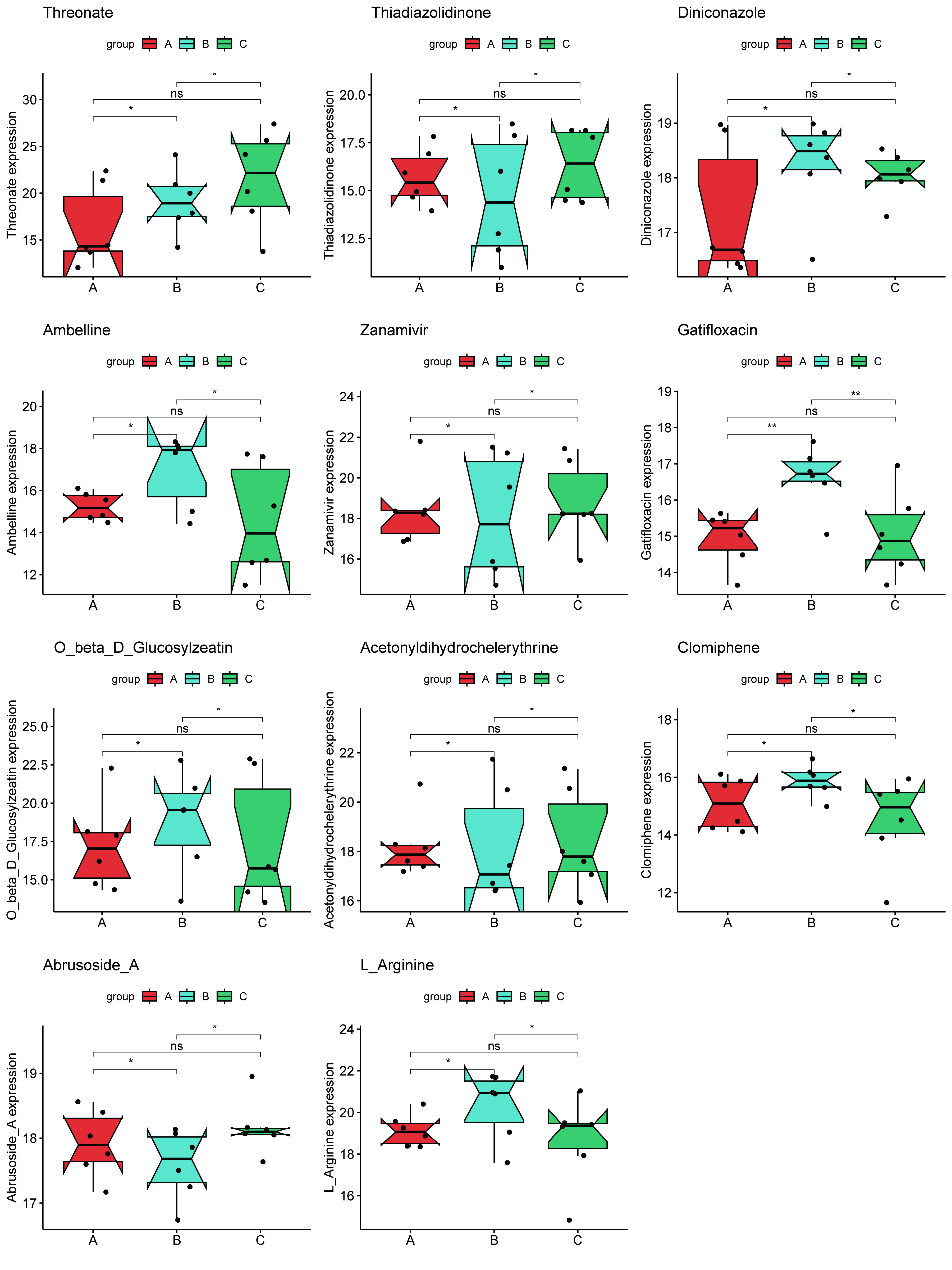

Supplement: Supplementary file 11 — Supplementary Material 11 [file 12967_2024_5425_MOESM11_ESM.tif]

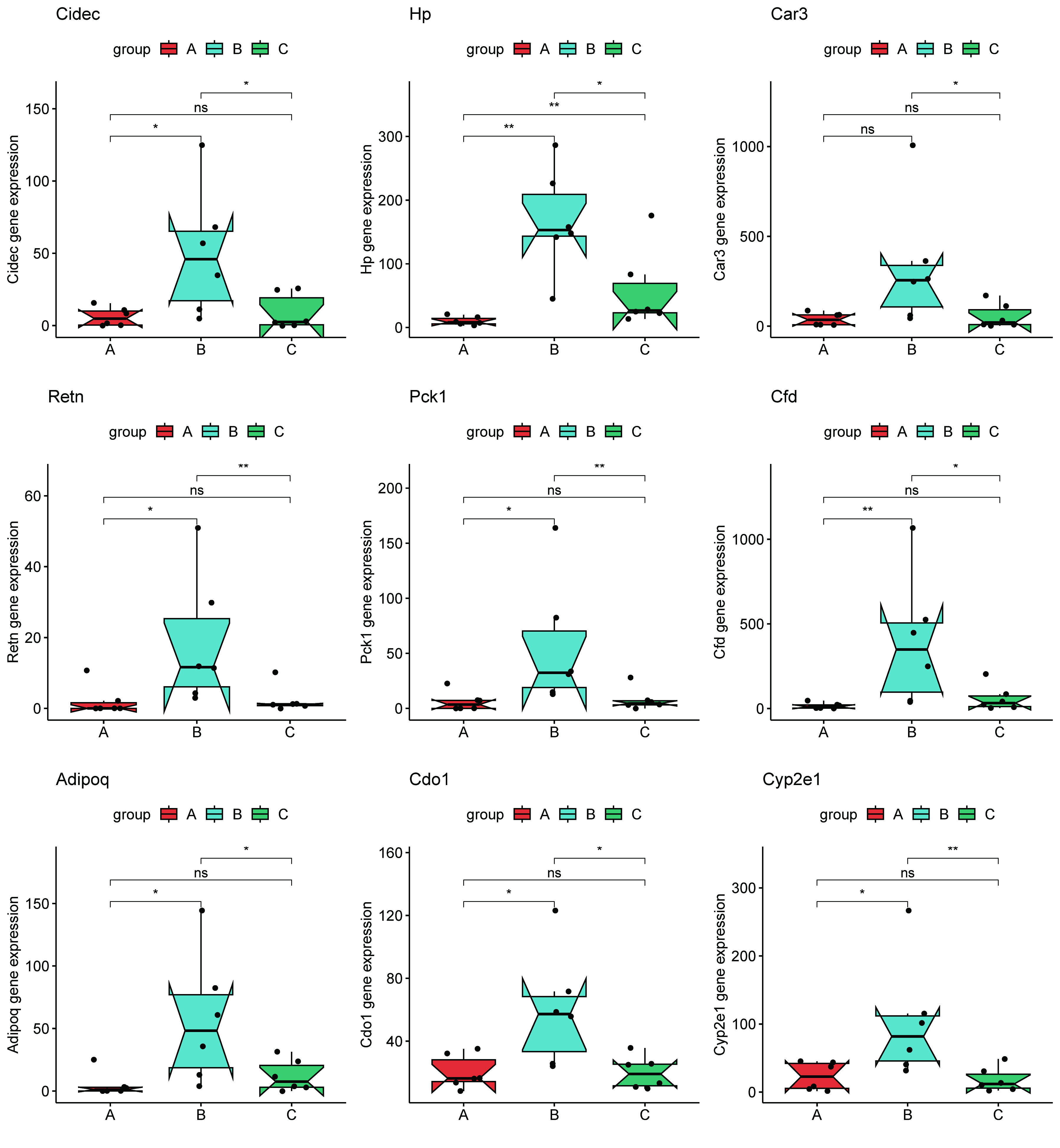

Supplement: Supplementary file 12 — Supplementary Material 12 [file 12967_2024_5425_MOESM12_ESM.tif]
