# Supplementary material for: Exploring the role of CBLB in acute myocardial infarction: transcriptomic, microbiomic, and metabolomic analyses
Source: J Transl Med. 2024 Jul 14;22:654. doi: 10.1186/s12967-024-05425-y (PMC11247792; doi:10.1186/s12967-024-05425-y)
Supplement: Supplementary file 10 — Supplementary Material 10 [file 12967_2024_5425_MOESM10_ESM.docx]

Dear Professor Francesco M. Marincola,

We sincerely appreciate your response and the valuable insights provided in your decision letter regarding our manuscript (Manuscript # JTRM-D-24-00756) entitled:

“*Exploring the Role of CBLB in Acute Myocardial Infarction: A Comprehensive Study on Transcriptome, Gut Microbiota, and Metabolomics*”

We extend our gratitude to the reviewers for their thoughtful comments and suggestions, which have greatly contributed to the improvement and clarity of our manuscript.

We have diligently incorporated all the suggested revisions and addressed each of the reviewers' comments in our revised manuscript.

We trust that the revised manuscript now meets the standards for publication in your esteemed journal and eagerly anticipate your feedback.

Thank you once again for your time and consideration.

Warm regards,

Wenqi Han

**We deeply appreciate the reviewers for their invaluable feedback and constructive suggestions. Enclosed below are our detailed responses addressing each concern raised in their comments.**

**Reviewer #1:** Reviewer's Comments

Manuscript Number: JTRM-D-24-00756

Full Title: Exploring the Role of CBLB in Acute Myocardial Infarction: A Comprehensive Study on Transcriptome, Gut Microbiota, and Metabolomics

Given the extensive and detailed nature of the manuscript "Exploring the Role of CBLB in Acute Myocardial Infarction: A Comprehensive Study on Transcriptome Gut Microbiota and Metabolomics," a thorough review requires a deep dive into its methodology, results, and conclusions. The study ambitiously integrates multiple omics approaches to elucidate the role of CBLB in the context of acute myocardial infarction (AMI), employing techniques such as transcriptomics, 16S rDNA sequencing for gut microbiota analysis, and non-targeted metabolomics sequencing.

**RE:** First and foremost, we would like to express our profound gratitude for the insightful comments and time you have dedicated to reviewing our manuscript, "Exploring the Role of CBLB in Acute Myocardial Infarction: A Comprehensive Study on Transcriptome Gut Microbiota and Metabolomics". We appreciate your recognition of the extensive and detailed nature of our work and the ambition of integrating multiple omics approaches to elucidate the role of CBLB in the context of acute myocardial infarction (AMI).

We understand that a thorough review of our manuscript requires a deep dive into its methodology, results, and conclusions. We have strived to ensure that our work is presented in a comprehensive and understandable manner, employing techniques such as transcriptomics, 16S rDNA sequencing for gut microbiota analysis, and non-targeted metabolomics sequencing.

In response to your comment, we are more than willing to provide additional details or clarification for any aspect of our study. We believe that your constructive feedback will significantly aid in enhancing the depth and quality of our research.

We hope that our manuscript will provide valuable insights into the role of CBLB in AMI, and we look forward to your further comments and suggestions, which we are confident will enhance the scientific integrity and overall quality of our study.

Thank you once again for your time and consideration.

General Comments:

1. Novelty and Impact: The study's interdisciplinary approach, combining transcriptomics, microbiota analysis, and metabolomics, provides a novel insight into the multifaceted role of CBLB in AMI. The potential identification of new biomarkers and therapeutic targets could significantly impact AMI management and treatment strategies.

**RE:** We greatly appreciate your time and careful consideration in reviewing our manuscript. We are encouraged by your recognition of the novelty and impact of our interdisciplinary approach.

We agree with your assessment that the combination of transcriptomics, microbiota analysis, and metabolomics provides a novel insight into the multifaceted role of CBLB in AMI. We have endeavored to ensure our research is rigorous and innovative, and we are delighted that you acknowledge this effort.

It is indeed our aspiration that the potential identification of new biomarkers and therapeutic targets could significantly impact AMI management and treatment strategies. Your recognition of this potential impact is extremely encouraging and validates our research direction.

We look forward to the opportunity to further refine our manuscript based on your valuable feedback. If there are any specific areas where you believe we could provide additional detail or clarity, we would be more than happy to do so.

Thank you once again for your constructive comments and for recognizing the potential impact of our study.

2. Methodological Rigor: The methods section is comprehensive, detailing the procedures for lentiviral vector construction, AMI model establishment in mice, and subsequent omics analyses. However, the manuscript would benefit from further clarification on the choice of analytical methods and the validation of findings in human samples or additional animal models.

**RE:** We sincerely appreciate the time and effort you have put into reviewing our manuscript. We are thankful for your constructive feedback and would like to respond to your concerns as follows:

Regarding your comment on the need for further clarification on the choice of analytical methods, we would like to clarify that the methods we utilized are conventional and widely accepted in this field. However, we understand the need for clarity and are willing to provide further elaboration on the specific analytical techniques employed. If you could kindly provide us with more details regarding which methods you believe require further explanation, we would be more than happy to address them in our revised manuscript.

As for your concern about the validation of findings in human samples or additional animal models, we indeed acknowledge this in the limitations section of our manuscript. As stated, "This study primarily investigates the regulatory mechanisms of CBLB in AMI using a mouse model. However, this model does not accurately represent the human physiological environment. Thus, it is necessary to validate our findings in larger samples and diverse models." Your comment reinforces the importance of this point and we appreciate your understanding of the constraints in resources and time that we faced in this current study.

We hope these clarifications address your concerns and we look forward to your further suggestions to improve our manuscript.

3. Statistical Analysis and Data Interpretation: The manuscript demonstrates a robust statistical analysis framework. Still, it would be advantageous to discuss the potential limitations of the statistical models used and the biological relevance of the statistical thresholds set for differential expression and metabolite identification.

**RE:** We sincerely appreciate your insightful comments and the recognition of the robustness in our statistical analysis framework in our manuscript.

Regarding the potential limitations of the statistical models used, we concur with your point. In our revised manuscript, we will include the following text in the limitations section: " While our statistical models provide valuable insights, it's important to acknowledge potential limitations. The used parametric tests and Spearman correlation analysis assume certain characteristics of the data which may not always hold true in biological studies. Also, despite multiple testing corrections, the risk of false positives remains due to the high-dimensional nature of the data. Additionally, in silico methods for data interpretation rely on existing databases, potentially missing novel interactions or pathways. We recommend cautious interpretation of the results and further validation through additional statistical methods or experimental confirmation.”

As for your comment on "the biological relevance of the statistical thresholds set for differential expression and metabolite identification", we would like to clarify that the statistical thresholds used in our study adhere to the widely accepted standards in the field. The thresholds we employed for determining differential expression and metabolite identification have been widely recognized in transcriptome and metabolomics studies. We believe that the established guidelines provide a balance between statistical rigor and biological relevance. Therefore, while we appreciate your suggestion, we respectfully consider that further discussion on this topic may not add significant value to the manuscript, given its established acceptance in the field.

We are grateful for your constructive feedback and look forward to improving the manuscript based on your suggestions.

4. Integration of Multi-omics Data: The study exemplifies the integration of multi-omics data to understand complex biological phenomena. Future directions could include the use of systems biology approaches to further explore the interactions between differentially expressed genes, metabolites, and microbiota changes, providing a more holistic view of the disease mechanism.

**RE:** We appreciate your constructive comments and suggestions on our manuscript entitled "Exploring the Role of CBLB in Acute Myocardial Infarction: A Comprehensive Study on Transcriptome Gut Microbiota and Metabolomics". We are grateful for your recognition of the integration of multi-omics data in understanding complex biological phenomena in our study.

Regarding the suggestion on the “Future directions could include the use of systems biology approaches to further explore the interactions between differentially expressed genes, metabolites, and microbiota changes, providing a more holistic view of the disease mechanism”, we completely concur.

In our original manuscript, we indeed acknowledged that our study suggests the correlation between CBLB's regulation of AMI and changes in gut microbes, metabolites, and genes. However, we also highlighted that these are only correlative observations and the specific molecular mechanisms remain elusive, necessitating further research.

In light of your insightful suggestion, we agree that a systems biology approach could potentially provide a more comprehensive and holistic view of the disease mechanism. This approach could reveal not just single correlations, but the intricate network of interactions among genes, metabolites, and microbiota, thereby giving us a more profound understanding of the regulation of CBLB in AMI.

We have revised the “Future Directions” section of our manuscript to reflect this perspective. We now state, " Hence, our future investigations will aim to utilize systems biology strategies to elucidate the intricate relationships among differentially expressed genes, metabolites, and microbiota alterations. This comprehensive approach could deepen our understanding of the disease mechanism."

We sincerely thank you for your astute suggestion and believe that this revision will strengthen our manuscript.

5. Clinical Relevance: While the study advances our understanding of CBLB's role in AMI, translating these findings into clinical practice requires a discussion on how these molecular insights could influence current AMI diagnosis, prognosis, and treatment paradigms.

**RE:** We sincerely appreciate your valuable feedback on our manuscript, "Exploring the Role of CBLB in Acute Myocardial Infarction: A Comprehensive Study on Transcriptome Gut Microbiota and Metabolomics."

In regard to your comment about the "Clinical Relevance", we concur that a discussion on how our molecular insights could influence current AMI diagnosis, prognosis, and treatment paradigms would indeed enhance the practical impact of our findings.

The molecular changes we observed in the regulation of CBLB in AMI could provide potential novel biomarkers for AMI diagnosis and prognosis. Additionally, understanding these mechanisms could open new avenues for therapeutic intervention. By targeting CBLB or associated pathways, it might be possible to modulate AMI progression.

We have expanded our manuscript to include a discussion on these potential clinical applications. Specifically, we have added the following text in the Discussion section:

"Our findings suggest potential clinical implications. If the molecular changes associated with CBLB's regulation of AMI are validated in further studies, they could provide novel biomarkers for AMI diagnosis and prognosis. Moreover, understanding these mechanisms could lead to new therapeutic strategies, such as targeting CBLB or its associated pathways to modulate AMI progression."

We are grateful for this insightful suggestion and believe that the revised manuscript now better highlights the potential clinical relevance of our findings.

Specific Comments:

*Experimental Design: The choice of controls and experimental groups is appropriate. However, details on the randomization process and blinding during the experiment and analysis phases would strengthen the study's validity.

**RE:** We are grateful for the reviewer's positive feedback on the appropriateness of our controls and experimental group selections. We understand and appreciate the reviewer's concern about the need for additional details on the randomization process and blinding during the experiment and analysis phases, as these details would indeed enhance the robustness and validity of our study.

To address this, we have now included detailed information on the randomization process in the revised manuscript. In brief, the randomization process was performed by an independent researcher who had no involvement in the data collection or analysis. This was done to ensure unbiased assignment of subjects to the experimental or control groups.

Regarding blinding, we have now clarified in the manuscript that all data analyses were performed in a blinded manner. Specifically, the research team who conducted the data analysis was not aware of which group the data belonged to until the statistical analysis was complete.

We believe these additional details not only address the reviewer's concerns but also substantially strengthen the reliability and validity of our study. We are grateful for the reviewer's constructive feedback and for the opportunity to clarify these important aspects of our study.

We hope these responses sufficiently address the reviewer's comments. We are open to any further suggestions and are committed to making any necessary changes to improve our manuscript.

Thank you for considering our responses and for the opportunity to improve our work.

*Reproducibility and Data Availability: The manuscript should state whether the data and code supporting the findings will be available to other researchers for reproducibility and further analysis.

**RE:** We are thankful to the reviewer for bringing up the important aspect of reproducibility and data availability. We understand the significance of transparency in research and the requirement of data and code availability for the reproducibility of the study and further analysis.

In the initial manuscript, we have addressed this point in the "Availability of data and materials" section, where we have mentioned, "The datasets used and/or analyzed during the current study are available from the corresponding author on reasonable request."

However, recognizing the reviewer's concern for more explicitness, we have now revised this section to state, "All raw data supporting the conclusions of our study, as well as any relevant code, will be made available to any researcher for the purpose of reproducing the results or replicating the procedures, upon reasonable request to the corresponding author."

We believe this revision will address the reviewer's concern and further ensure the reproducibility and transparency of our research.

We appreciate the opportunity to clarify this aspect of our study and are open to any additional comments or suggestions.

Thank you for your time and consideration.

*Figures and Tables: While the figures and tables provided are informative, additional graphical representations of the multi-omics data integration and the pathways implicated by the study's findings would enhance the reader's understanding.

**RE:** We are grateful to the reviewer for the constructive suggestion to improve the clarity of our manuscript through additional graphical representations. We completely agree with the reviewer's point that such visual aids can significantly enhance the reader's comprehension of complex multi-omics data integration and the implicated pathways.

In response to this, we have added a comprehensive flow chart that graphically represents the research process, from data collection to analysis and interpretation. This figure provides a clear depiction of the multi-omics data integration process.

Regarding the suggestion to include a schematic diagram illustrating the pathways implicated by our study's findings, we understand and appreciate its importance. However, due to the complexity and heterogeneity of the data, creating such a diagram that accurately represents the implicated pathways is a challenging task. We are striving to overcome this challenge and plan to include such a diagram in our future work.

We believe that the added flow chart will significantly improve the clarity and comprehensibility of our manuscript, addressing the reviewer's concern to a large extent.

We look forward to any further comments or suggestions.

*Literature Context: Placing the findings within the broader context of existing literature on AMI, gut microbiota, and metabolomics research would help highlight the study's contribution to the field. Comparing and contrasting the results with those of similar studies could provide insights into the novelty and significance of the findings.

**RE:** We sincerely thank the reviewer for the insightful suggestion to position our findings within the broader context of the existing literature on AMI, gut microbiota, and metabolomics research, thereby underscoring the study's contribution to the field.

In our study, we delve into the functional role of CBLB in the AMI mouse model and its potential regulatory mechanisms. Our approach involved gathering data from transcriptome, 16S microbial sequencing, and untargeted metabolomics sequencing. To our understanding, our study stands unique in its approach and findings, hence, direct comparisons with existing studies pose a challenge.

In light of this, our discussion primarily focuses on the key genes, metabolites, and microbial groups identified in our study that find mention in the existing literature. However, recognizing the reviewer's comment, we have revised our discussion, " In this study, our focus was to investigate the functional role and potential regulatory mechanisms of CBLB in the AMI mouse model. To achieve this, we employed a distinctive combination of transcriptome, 16S microbial sequencing, and untargeted metabolomics sequencing data. To the best of our knowledge, no previous studies have explored the role of CBLB in AMI using this unique multi-omics approach. Our findings suggest that overexpression of CBLB significantly lessens myocardial infarction in AMI mice, modifies gut microbial abundance, and influences the expression of certain metabolites and genes.". We believe this explanation provides a clearer context for our study's novelty and significance.

We appreciate the reviewer's constructive comments and welcome any further suggestions that could enhance our manuscript.

Conclusion:

This manuscript provides valuable insights into the role of CBLB in AMI, employing a comprehensive multi-omics approach. While the study is methodologically sound, further clarification on experimental details, data integration, and the translational pathway to clinical application would enhance its impact. The research represents a significant step forward in understanding the complex biological mechanisms underlying AMI and opens avenues for future studies focusing on therapeutic interventions targeting the CBLB pathway.

**RE:** We are sincerely grateful for the valuable feedback and constructive comments on our manuscript. We have taken them into careful consideration and made corresponding revisions. Below is our point-by-point response:

1. In response to the request for more detailed experimental information, we have elaborated on the randomization and blinding methods used in our study, and have updated the data availability statement. This information can now be found in the revised manuscript.

2. To address the suggestion on data integration, we have included a research flow chart in the manuscript, which we believe will facilitate a quicker and more comprehensive understanding of the study's process. Regarding the comparison with similar studies, we fully agree with the importance of this perspective. However, given the novelty of our approach in investigating the role of CBLB in AMI using a comprehensive multi-omics approach, we have yet to find comparable studies for a direct comparison. This further underscores the uniqueness of our research.

3. In response to the suggestion for the "translational pathway to clinical application", we appreciate the importance of including a schematic diagram to illustrate the pathways investigated in our study. We agree that such a diagram would be beneficial for the understanding and propagation of our research. However, we would like to point out that due to the complexity of the relationships between CBLB, key genes, metabolites, and microbes, accurately representing these implicated pathways in a diagram is a challenging task. While we are striving to overcome this challenge, it is not feasible to include such a diagram in the present version of the manuscript. We aim to include this in our future work after further research and analysis. We believe this approach will ensure that the diagram is both accurate and informative.

4. Detailed responses to the suggestions and/or revisions can be found in the "Specific Comments" section mentioned above.

We trust that these revisions and responses address the reviewers' concerns adequately, and enhance the clarity and impact of our manuscript. We thank the reviewers once again for their insightful suggestions, which have undoubtedly improved the quality of our work.

More Comments

Based on the detailed content of the manuscript "Exploring the Role of CBLB in Acute Myocardial Infarction: A Comprehensive Study on Transcriptome Gut Microbiota and Metabolomics," here are several comprehensive, detailed, and critical comments addressing the requested aspects:

1. Data Validation

The study employs rigorous methods for data validation, including differential expression analysis and protein-protein interaction networks. However, the validation of key findings in human subjects or additional animal models could enhance the robustness of the conclusions.

**RE:** Thank you for your constructive feedback regarding the validation of our key findings in human subjects or additional animal models. We appreciate the value of such validation processes and fully agree that they have the potential to enhance the robustness of our conclusions.

However, we would like to bring to your attention that there are currently several constraints that make the execution of such validation studies challenging. First, the financial resources available for this research are quite limited, which has restricted our ability to conduct additional experiments.

Furthermore, our findings represent a novel and complex interplay between CBLB, key genes, metabolites, and microbes. The validation of these interactions in human subjects or additional animal models would require a well-designed and meticulous approach to ensure the accuracy of the results. Given the complexity and novelty of this interplay, the development of such an approach is time-consuming and is currently in progress.

While we recognize the importance of such validation studies and are committed to pursuing them in future research, we believe that our current findings are significant and novel, and contribute to the understanding of the role of CBLB in AMI. We trust that the rigorous methods we employed for data validation in our study, including differential expression analysis and protein-protein interaction networks, are sufficient to support the conclusions we draw in our manuscript.

We hope that the reviewers will consider these factors and accept our current work with the understanding that validation studies are planned for the future when resources allow.

Thank you for your understanding and consideration.

2. Data Integration

The integration of transcriptomic, microbiota, and metabolomic data is commendable, offering a holistic view of AMI's pathophysiology. Future studies could benefit from advanced computational models to further explore the complex interactions between these datasets.

**RE:** We express our gratitude for your positive feedback on our integrated approach to studying the pathophysiology of AMI and for your insightful suggestion to employ advanced computational models in future studies.

With regard to your suggestion of using advanced computational models to further explore the complex interactions between these datasets in future studies, we wholeheartedly agree. We acknowledge that such models can offer a more detailed and nuanced understanding of the data, potentially uncovering novel insights into the pathophysiology of AMI.

In fact, we are in the process of familiarizing ourselves with advanced computational tools and techniques that could help us better analyze our data. We also plan to collaborate with researchers specialized in computational biology to ensure that these analyses are conducted accurately and rigorously.

While we strive to incorporate these sophisticated analyses in our future work, we believe that the present study, with the integration of transcriptomic, microbiota, and metabolomic data, has been conducted rigorously and provides valuable insights. We acknowledge that the application of advanced computational models could further strengthen our research, but we hope that the current findings are seen as a step towards that direction and contribute to the existing body of knowledge.

We appreciate your understanding and look forward to further enhancing our research methodologies in future studies.

3. Strengths and Significance

A major strength is the comprehensive multi-omics approach, which provides novel insights into the role of CBLB in AMI. This study could pave the way for new diagnostic and therapeutic strategies, highlighting its significant potential impact on cardiovascular disease research.

**RE:** We are profoundly grateful for your positive feedback on our research. We are pleased to see that you acknowledge the strengths of our multi-omics approach and its potential to pave the way for new diagnostic and therapeutic strategies for AMI.

We fully agree with your assessment of the significance of our study in the context of cardiovascular disease research. Indeed, our aim was to provide novel insights into the role of CBLB in AMI, and we believe that our comprehensive multi-omics approach has allowed us to achieve this.

In the future, we intend to continue to employ and refine this approach to further explore the complex mechanisms underlying AMI. We are confident that this will lead to the identification of additional potential therapeutic targets and diagnostic markers, thereby further contributing to the advancement of cardiovascular disease research.

Once again, we deeply appreciate your acknowledgment of the strengths and significance of our work. We trust that our study provides a meaningful contribution to the field and paves the way for future research in this area.

4. Novelty

The manuscript presents novel findings on the role of CBLB in AMI, particularly its influence on gut microbiota and metabolomic profiles. This contributes new knowledge to the field of cardiovascular disease and immune regulation.

**RE:** We would like to extend our sincere thanks for your positive assessment of the novelty of our research and its contribution to the field of cardiovascular disease and immune regulation.

We are indeed pleased that you acknowledge the novel findings our study presents, particularly the role of CBLB in AMI and its influence on gut microbiota and metabolomic profiles. It was our aim from the outset to shed light on these complex interactions, and we are gratified that our work has been recognized as contributing new knowledge to the field.

In response to your comments, we would like to assure you that we will continue to explore these novel interactions in our future research. We believe that further elucidation of these mechanisms will not only deepen the understanding of the pathophysiology of AMI but also provide a foundation for the development of innovative therapeutic strategies.

Once again, we deeply appreciate your positive acknowledgment of the novelty and significance of our work. We believe that our study provides a fresh perspective on the role of CBLB in AMI and hope that it will inspire further research in this area.

5. Significance

The study's findings on CBLB's regulatory mechanisms in AMI have significant implications for understanding cardiovascular disease pathogenesis and identifying potential therapeutic targets.

**RE:** We wish to express our sincere gratitude for your positive assessment of the significance of our study, particularly in relation to understanding the pathogenesis of cardiovascular disease and identifying potential therapeutic targets.

We concur with your evaluation that our findings on the regulatory mechanisms of CBLB in AMI have important implications. It has always been our goal to provide novel insights into the pathophysiology of cardiovascular diseases, and we are pleased to have achieved this in our current study.

In response to your comments, we would like to assure you that we will continue to build on these findings in our future research. We aim to further elucidate the complex role of CBLB in the pathogenesis of AMI and other cardiovascular diseases, with the ultimate goal of identifying novel therapeutic targets. We believe that these efforts will significantly contribute to the ongoing fight against cardiovascular diseases.

Once again, we deeply appreciate your recognition of the significance of our work. We are committed to conducting research that not only adds to the existing body of knowledge but also has practical implications for the diagnosis and treatment of cardiovascular diseases.

6. Drawbacks

While comprehensive, the study's reliance on an animal model may limit the direct translatability of findings to human AMI pathology. The complexity of human disease necessitates cautious interpretation of results.

**RE:** We appreciate your insightful comments regarding the limitations associated with the use of an animal model in our study. We fully understand the inherent differences between human and animal disease pathology and agree that the complexity of human disease requires a cautious interpretation of our results.

In our study, we utilized an animal model as a necessary preliminary step to investigate the role of CBLB in AMI. We believe that despite the limitations, our animal model provided valuable information that contributes to our understanding of CBLB's regulatory mechanisms in AMI.

However, we acknowledge that translatability of findings from animal models to human disease is a complex and challenging process. We agree that future studies are needed to validate and extend our findings in human subjects to ensure their clinical relevance.

We assure you that we are committed to advancing our research in this direction. While we view our current study as a critical stepping stone, we plan to incorporate additional models, including human studies, in our future research to further validate our findings.

Your point about the necessity of cautious interpretation of results is well taken. In our manuscript, we have tried to present our findings with this caution in mind, emphasizing that while our results are promising, they represent a step in the ongoing exploration of the role of CBLB in AMI.

We thank you for your understanding and constructive feedback, which will undoubtedly guide our future research endeavors.

7. Best Solution

Further research should include validation studies in human subjects and exploration of CBLB's role across different stages of AMI. Additionally, investigating the therapeutic potential of modulating CBLB activity in cardiovascular disease could be highly valuable.

**RE:** We sincerely appreciate your insightful suggestions for further research on the role of CBLB in AMI. We fully agree that validation studies in human subjects and exploration of CBLB's role across different stages of AMI would add significant value to our research.

Indeed, we are already in the planning stages for further research in this direction. We aim to validate our current findings in human subjects and to explore the temporal role of CBLB in the progression of AMI. We believe that these studies will not only validate our current findings but also provide a more comprehensive understanding of the role of CBLB in AMI.

Furthermore, your suggestion to investigate the therapeutic potential of modulating CBLB activity in cardiovascular disease is highly valuable. We fully agree that this could open up new avenues for the development of innovative therapeutic strategies for AMI and other cardiovascular diseases. This is a direction that we are excited to explore in our future research.

Once again, we express our gratitude for your constructive feedback and valuable suggestions. We are committed to advancing our research in the directions you have suggested and believe that this will significantly enhance our understanding of the role of CBLB in AMI.

8. Reproducibility

The detailed methodology ensures reproducibility, yet making raw data and code publicly available would further strengthen this aspect, enabling other researchers to validate and build upon the findings.

**RE:** We are sincerely appreciative of your positive recognition of the reproducibility of our work due to our detailed methodology. We are also thankful for your suggestion to make raw data and code publicly available, a point which we fully embrace as we acknowledge the importance of transparency and accessibility in advancing science.

In our original manuscript, as per the journal's requirements, we clarified in the "Availability of data and materials" section that "The datasets used and/or analyzed during the current study are available from the corresponding author on reasonable request." However, in light of your comment and our commitment to promote open science, we have revised this section to further clarify the availability of our data and code.

The revised text now reads: "All raw data supporting the conclusions of our study, as well as any relevant code, will be made available to any researcher for the purpose of reproducing the results or replicating the procedures, upon reasonable request to the corresponding author."

We trust that this revision will more clearly communicate our commitment to data and code sharing, and we hope that this will enable other researchers to validate and build upon our findings.

Once again, we thank you for your constructive feedback and valuable suggestion, which will undoubtedly strengthen the reproducibility and credibility of our work.

9. Potentially Further and Futuristic Researches

Future research could explore the mechanistic pathways linking CBLB with gut microbiota alterations and metabolomic changes, potentially uncovering new therapeutic targets. Longitudinal studies assessing the impact of CBLB modulation pre- and post-AMI could offer insights into its therapeutic potential.

**RE:** We are sincerely grateful for your thoughtful comments regarding potential future research directions. Your suggestion to further explore the mechanistic pathways linking CBLB with gut microbiota alterations and metabolomic changes is indeed a compelling avenue of investigation, and we fully agree that this could potentially uncover new therapeutic targets.

In our future work, we plan to delve deeper into these mechanisms, which we anticipate will not only enrich our understanding of the complex interface between CBLB and gut microbiota in AMI, but also present new potential therapeutic strategies for this prevalent condition.

Additionally, your point regarding longitudinal studies assessing the impact of CBLB modulation pre- and post-AMI is well taken. Such studies would indeed offer valuable insights into the therapeutic potential of CBLB modulation. We are excited about the possibility of conducting such longitudinal studies and appreciate your suggestion to incorporate this approach into our future research plans.

We are committed to progressing our research along these lines and believe that these future studies will significantly enhance our understanding of the role of CBLB in AMI and its potential as a therapeutic target.

Once again, we express our gratitude for your insightful comments and constructive suggestions, which will undoubtedly guide our future research endeavors.

10. Conclusions and Recommendations

The manuscript convincingly demonstrates the multifaceted role of CBLB in AMI, offering new directions for research into cardiovascular disease mechanisms and treatment. Recommendations for future work include focusing on the translational potential of these findings, exploring therapeutic interventions targeting CBLB, and validating key results in human studies.

**RE:** We sincerely appreciate your positive evaluation of our manuscript and the recognition of the multifaceted role of CBLB in AMI that we have demonstrated.

We acknowledge your recommendations for future work and fully concur with the importance of focusing on the translational potential of our findings. As we strive to contribute meaningfully to the body of knowledge on cardiovascular disease mechanisms, we understand that translating our findings into clinical practice is of paramount importance. We assure you that we are committed to exploring therapeutic interventions targeting CBLB in our future research, with the aim of translating our findings into potential novel treatments for AMI.

Moreover, your suggestion to validate key results in human studies is well received. We are in agreement that such validation is crucial to ensure the clinical relevance of our findings and to bridge the gap between preclinical findings and clinical application. We are planning future studies with this intent.

Once again, we express our gratitude for your constructive feedback and insightful suggestions. We believe that your recommendations will guide our future research and help us to maximize the impact of our work in the field of cardiovascular disease.

Review and Critical Analysis of Title, Abstract, and Keywords

Title:

"Exploring the Role of CBLB in Acute Myocardial Infarction: A Comprehensive Study on Transcriptome, Gut Microbiota, and Metabolomics"

*	Evaluation: The title effectively encapsulates the study's broad objectives and the multi-omics approach employed. It clearly communicates the focus on CBLB and its implications in acute myocardial infarction (AMI), guiding the reader on the study's scope. However, the term "comprehensive" could be subjective; detailing the specific omics techniques (e.g., "Transcriptomic, Microbiomic, and Metabolomic Analyses") might offer more precise insight into the study's methodologies.

**RE:** We are grateful for your acknowledgment of the effectiveness of our title in encapsulating the study's broad objectives and the multi-omics approach employed. We appreciate your suggestion to further refine the title to offer more precise insight into the study's methodologies.

We understand the potential subjectivity of the term "comprehensive" and agree that replacing it with the specific omics techniques used in our study would provide more clarity. Hence, we have revised the title as follows:

"Exploring the Role of CBLB in Acute Myocardial Infarction: Transcriptomic, Microbiomic, and Metabolomic Analyses"

We believe this revised title concisely communicates the focus of our study and the methodologies employed while maintaining the original emphasis on the role of CBLB in AMI.

Once again, we thank you for your constructive feedback and insightful suggestions, which have helped to improve the clarity and precision of our manuscript.

Abstract:

* Background: The background succinctly outlines the rationale for the study, emphasizing the relevance of gut microbiota and metabolites in AMI and the potential role of CBLB. This section effectively sets the stage for the study, though it could benefit from a brief mention of existing gaps in the literature to strengthen the justification for the research.

**RE:** We appreciate the reviewer's positive feedback on our background, and we certainly agree with the suggestion to strengthen our justification by mentioning the existing gaps in the literature. Therefore, we have revised our abstract as follows:

"Specific alterations in gut microbiota and metabolites have been linked to AMI, with CBLB potentially playing an essential role. However, the precise interactions remain understudied, creating a significant gap in our understanding. This study aims to address this by exploring these interactions in CBLB-intervened AMI mice using transcriptome sequencing, 16S rDNA, and non-targeted metabolite analysis."

We believe this revision will provide a clearer understanding of the impetus behind our research and the significance of our findings in filling the existing research gaps. We greatly value your feedback and hope that our revisions meet your approval.

Thank you again for your time and consideration.

* Methods: The methods summary provides a clear overview of the experimental design, including the intervention and analysis strategies. However, it might be enhanced by briefly stating the rationale behind choosing these specific methods and any novel aspects of the experimental approach.

**RE:** We fully understand the reviewer's comment and agree that the rationale behind the methodological choices and the novelty of the experimental approach should be elaborated more clearly in the abstract. Accordingly, we have revised the Methods section of our abstract as follows:

"Methods: To probe the therapeutic potential and mechanistic underpinnings of CBLB overexpression in AMI, we utilized an integrative multi-omics strategy encompassing transcriptomics, metabolomics, and 16s rDNA sequencing. We selected these particular methods as they facilitate a holistic comprehension of the intricate interplay between the host and its microbiota, and the potential effects on the host's metabolic and gene expression profiles. The uniqueness of our investigation stems from utilizing a multi-omics approach to illuminate the role of CBLB in AMI, an approach yet unreported to the best of our knowledge. Our experimental protocol encompassed transfection of CBLB lentivirus-packaged vectors into 293T cells, followed by subsequent intervention in AMI mice. Subsequently, we conducted pathological staining, fecal 16s rDNA sequencing, and serum non-targeted metabolome sequencing. We applied differential expression analysis to discern differentially expressed genes (DEGs), differential metabolites, and differential microbiota. We performed protein-protein interaction analysis to identify core genes, and conducted correlation studies to clarify the relationships amongst these core genes, paramount metabolites, and key microbiota."

We believe this revised description is more transparent and informative, providing a clear rationale for our methodological choices and highlighting the novelty of our experimental approach. We hope this revision is in line with the reviewer's suggestion and enhances the overall quality of our abstract.

Once again, we express our gratitude for your valuable comments and look forward to your further feedback.

* Results: This section concisely summarizes the key findings, including the effects of CBLB intervention on the infarcted region, key microbiota, metabolites, and DEGs. While informative, the presentation of numerical outcomes (e.g., "650 DEGs1 and 143 DEGs2") without context may be overwhelming for readers not yet familiar with the study's details. Clarifying the significance of these results in lay terms could improve accessibility.

**RE:** We understand the reviewer’s concern that numerical data can be overwhelming and may not provide an accessible understanding of the results for those unfamiliar with the subject. To address this, we have revised the abstract to give a clearer, less data-intensive overview of the results. The revised abstract now reads as follows:

"Following the intervention of CBLB in AMI, we observed a significant decrease in inflammatory cell infiltration and collagen fiber formation in the infarcted region of mice hearts. We identified key changes in microbiota, metabolites, and differentially expressed genes (DEGs) that were associated with this intervention. The findings revealed that CBLB has a significant correlation with DEGs, differential metabolites and microbiota, respectively. This suggests it could play a pivotal role in the regulation of AMI."

We believe this revised abstract provides a more concise and accessible summary of our results while still conveying the key findings of our research.

We sincerely thank you and the reviewer for the constructive suggestions. We believe these changes have improved the clarity and accessibility of our paper.

* Conclusion: The conclusion highlights the study's contributions to understanding AMI regulation via CBLB intervention. It could be strengthened by explicitly stating the potential implications for AMI treatment or future research directions, providing a more compelling closure to the abstract.

**RE:** Thank you for your constructive feedback and suggestions on our manuscript. We appreciate the time and effort you have put into reviewing our work. We have carefully considered your comments on the conclusion section of our abstract and agree that it would be strengthened by including more explicit implications for AMI treatment and future research directions.

The original text was:

"Conclusion: This study, we validated the potential efficacy of multiple differential genes, metabolites, and microbiota in regulation of AMI after CBLB intervention, which provided a foundation to explore the regulatory mechanism of CBLB in AMI."

In response to your comments, we propose to revise the conclusion as follows:

"Conclusion: This study confirmed the potential of differentially expressed genes, metabolites, and microbiota in AMI regulation post-CBLB intervention. Our findings lay groundwork for future exploration of CBLB's role in AMI, suggesting potential therapeutic applications and novel research directions in AMI treatment strategies."

We believe this revised conclusion provides a more comprehensive summary of our study's implications and potential impact on the field.

We appreciate your insightful feedback, which has undoubtedly improved the quality of our manuscript. We hope that the revised conclusion now meets the standards of the Journal of Translational Medicine and look forward to further comments.

Keywords:

"Acute myocardial infarction; RNA-seq; 16S rDNA sequencing; Non-targeted metabolites sequencing; Biomarkers"

* Evaluation: The keywords are well-chosen, covering the main study topics and methodologies. They are likely to facilitate the study's discovery via relevant search queries. Adding "CBLB" as a keyword could further enhance the article's visibility to researchers specifically interested in this gene's role in AMI.

**RE:** We are grateful for your insightful comments and the time you've invested in reviewing our manuscript. We appreciate your positive feedback on the choice of keywords and your suggestion to include "CBLB" to further enhance our article's visibility.

In agreement with your suggestion, we recognize that including "CBLB" as a keyword would indeed make our study more accessible to researchers specifically interested in the role of this gene in AMI. Consequently, we have added "CBLB" to our list of keywords. The revised keyword list is as follows: "Acute myocardial infarction; RNA-seq; 16S rDNA sequencing; Non-targeted metabolites sequencing; Biomarkers; CBLB".

We believe this addition will further enhance the discoverability of our study among researchers in this specific area of interest and are confident that the revised list of keywords accurately reflects the central topics and methodologies of our study.

We hope that these revisions meet with your approval and look forward to any further suggestions you may have.

Recommendations for Revision:

1. Title Adjustment: Consider specifying the omics techniques in the title for greater precision, e.g., "Investigating CBLB's Role in Acute Myocardial Infarction Through Transcriptomic, Microbiomic, and Metabolomic Profiling".

**RE:** We sincerely appreciate your time and effort in providing valuable feedback and suggestions on our manuscript. We are grateful for your acknowledgment of the effectiveness of our title in encapsulating the study's broad objectives and the multi-omics approach employed. We have carefully considered your comments and agree with your perspective. As such, we have made necessary amendments that we believe have improved the precision and clarity of our manuscript.

Specifically, you suggested that the term "comprehensive" could be subjective and recommended that we replace it with a more precise description of the omics techniques employed in our study. We concur with your viewpoint, recognizing that the explicit mention of the specific omics techniques used would enhance the clarity of our study's methodologies.

As such, we have revised the title to: "Exploring the Role of CBLB in Acute Myocardial Infarction: Transcriptomic, Microbiomic, and Metabolomic Analyses". We believe this revised title succinctly communicates the focus of our study and the methodologies employed, while maintaining the original emphasis on the role of CBLB in AMI.

We would like to express our gratitude once again for your constructive feedback and insightful suggestions. We believe that these changes have significantly improved our manuscript and made it more comprehensible for the readers. We look forward to further comments and suggestions that will help us to refine our work.

2. Abstract Enhancement:

* Background: Include a brief mention of the gap in current knowledge or the novel aspect of your study.

**RE:** We appreciate the reviewer's positive feedback on our background, and we certainly agree with the suggestion to strengthen our justification by mentioning the existing gaps in the literature. Therefore, we have revised our abstract as follows:

"Specific alterations in gut microbiota and metabolites have been linked to AMI, with CBLB potentially playing an essential role. However, the precise interactions remain understudied, creating a significant gap in our understanding. This study aims to address this by exploring these interactions in CBLB-intervened AMI mice using transcriptome sequencing, 16S rDNA, and non-targeted metabolite analysis."

We believe this revision will provide a clearer understanding of the impetus behind our research and the significance of our findings in filling the existing research gaps. We greatly value your feedback and hope that our revisions meet your approval.

Thank you again for your time and consideration.

* Methods: Briefly explain the choice of methods or any novel aspects of your approach.

**RE:** We fully understand the reviewer's comment and agree that the rationale behind the methodological choices and the novelty of the experimental approach should be elaborated more clearly in the abstract. Accordingly, we have revised the Methods section of our abstract as follows:

"Methods: To probe the therapeutic potential and mechanistic underpinnings of CBLB overexpression in AMI, we utilized an integrative multi-omics strategy encompassing transcriptomics, metabolomics, and 16s rDNA sequencing. We selected these particular methods as they facilitate a holistic comprehension of the intricate interplay between the host and its microbiota, and the potential effects on the host's metabolic and gene expression profiles. The uniqueness of our investigation stems from utilizing a multi-omics approach to illuminate the role of CBLB in AMI, an approach yet unreported to the best of our knowledge. Our experimental protocol encompassed transfection of CBLB lentivirus-packaged vectors into 293T cells, followed by subsequent intervention in AMI mice. Subsequently, we conducted pathological staining, fecal 16s rDNA sequencing, and serum non-targeted metabolome sequencing. We applied differential expression analysis to discern differentially expressed genes (DEGs), differential metabolites, and differential microbiota. We performed protein-protein interaction analysis to identify core genes, and conducted correlation studies to clarify the relationships amongst these core genes, paramount metabolites, and key microbiota."

We believe this revised description is more transparent and informative, providing a clear rationale for our methodological choices and highlighting the novelty of our experimental approach. We hope this revision is in line with the reviewer's suggestion and enhances the overall quality of our abstract.

Once again, we express our gratitude for your valuable comments and look forward to your further feedback.

* Results: Provide context for numerical results to make them more accessible and indicate their significance.

**RE:** We understand the reviewer’s concern that numerical data can be overwhelming and may not provide an accessible understanding of the results for those unfamiliar with the subject. To address this, we have revised the abstract to give a clearer, less data-intensive overview of the results. The revised abstract now reads as follows:

"Following the intervention of CBLB in AMI, we observed a significant decrease in inflammatory cell infiltration and collagen fiber formation in the infarcted region of mice hearts. We identified key changes in microbiota, metabolites, and differentially expressed genes (DEGs) that were associated with this intervention. The findings revealed that CBLB has a significant correlation with DEGs, differential metabolites and microbiota, respectively. This suggests it could play a pivotal role in the regulation of AMI."

We believe this revised abstract provides a more concise and accessible summary of our results while still conveying the key findings of our research.

We sincerely thank you and the reviewer for the constructive suggestions. We believe these changes have improved the clarity and accessibility of our paper.

* Conclusion: Explicitly mention the implications of your findings for AMI treatment or future research avenues.

**RE:** Thank you for your constructive feedback and suggestions on our manuscript. We appreciate the time and effort you have put into reviewing our work. We have carefully considered your comments on the conclusion section of our abstract and agree that it would be strengthened by including more explicit implications for AMI treatment and future research directions.

The original text was:

"Conclusion: This study, we validated the potential efficacy of multiple differential genes, metabolites, and microbiota in regulation of AMI after CBLB intervention, which provided a foundation to explore the regulatory mechanism of CBLB in AMI."

In response to your comments, we propose to revise the conclusion as follows:

"Conclusion: This study confirmed the potential of differentially expressed genes, metabolites, and microbiota in AMI regulation post-CBLB intervention. Our findings lay groundwork for future exploration of CBLB's role in AMI, suggesting potential therapeutic applications and novel research directions in AMI treatment strategies."

We believe this revised conclusion provides a more comprehensive summary of our study's implications and potential impact on the field.

We appreciate your insightful feedback, which has undoubtedly improved the quality of our manuscript. We hope that the revised conclusion now meets the standards of the Journal of Translational Medicine and look forward to further comments.

3. Keywords Expansion: Add "CBLB" to the keywords to target researchers interested in this specific gene.

**RE:** We are grateful for your insightful comments and the time you've invested in reviewing our manuscript. We appreciate your positive feedback on the choice of keywords and your suggestion to include "CBLB" to further enhance our article's visibility.

In agreement with your suggestion, we recognize that including "CBLB" as a keyword would indeed make our study more accessible to researchers specifically interested in the role of this gene in AMI. Consequently, we have added "CBLB" to our list of keywords. The revised keyword list is as follows: "Acute myocardial infarction; RNA-seq; 16S rDNA sequencing; Non-targeted metabolites sequencing; Biomarkers; CBLB".

We believe this addition will further enhance the discoverability of our study among researchers in this specific area of interest and are confident that the revised list of keywords accurately reflects the central topics and methodologies of our study.

We hope that these revisions meet with your approval and look forward to any further suggestions you may have.

By addressing these recommendations, the title, abstract, and keywords can be optimized to more effectively communicate the study's significance, methodology, and findings, enhancing its impact and accessibility within the scientific community.

**RE:** We are sincerely grateful for your thoughtful recommendations and the time you have invested in reviewing our manuscript. We agree that by taking into account your suggestions, the title, abstract, and keywords will more effectively communicate our study's significance, methodology, and findings, thus enhancing its impact and accessibility within the scientific community.

In response to your feedback, we have made the following revisions:

1. Title: We have revised the title to include the specific omics techniques used in our study for greater precision and clarity.

2. Abstract: We have refined the abstract to more clearly and succinctly communicate our study's significance, the methods employed, and the key findings. We have also made sure to highlight the potential implications of our study for AMI treatment and future research directions.

3. Keywords: As per your suggestion, we have included "CBLB" as one of our keywords to enhance the visibility of our study to researchers specifically interested in this gene's role in AMI.

We believe these revisions have significantly improved the clarity and impact of our manuscript. We greatly appreciate your constructive feedback, which has undoubtedly helped in enhancing the quality of our work. We look forward to any further comments or suggestions you may have.

Review and Critical Analysis of the Introduction Section

Evaluation of the Introduction

1. Context and Relevance: The introduction effectively sets the stage by highlighting acute myocardial infarction (AMI) as a severe cardiovascular condition, emphasizing the need for novel diagnostic and therapeutic approaches. The mention of the American Heart Association's statistics on AMI incidence provides a quantitative grasp of the condition's prevalence, underscoring the clinical significance of the research.

**RE:** We appreciate your valuable comments and the time you have invested in reviewing our manuscript. We are grateful for your positive feedback on the introduction of our study, specifically noting its effectiveness in setting the context and relevance of our research in relation to acute myocardial infarction (AMI).

It is indeed our intention to highlight the severity of AMI as a cardiovascular condition and emphasize the necessity for novel diagnostic and therapeutic approaches. We are pleased that our inclusion of the American Heart Association's statistics on AMI incidence effectively underscored the clinical significance of our research and provided a quantitative grasp of the condition's prevalence.

Your positive feedback encourages us, and we believe it is a testament to the potential impact and relevance of our study. We look forward to further suggestions and comments to help us improve our manuscript.

2. Introduction of Cbl-b: The transition to discussing Casitas B lymphoma-b (Cbl-b) is smooth, providing a clear rationale for focusing on this particular E3 ubiquitin ligase. The explanation of Cbl-b's role in immune cell regulation and its potential as an AMI biomarker is concise yet informative, offering a strong scientific premise for the study. However, the introduction could be enhanced by elaborating on the direct link between Cbl-b's immunomodulatory functions and its relevance to AMI pathophysiology, thereby strengthening the justification for its selection as a study focus.

**RE:** We appreciate your positive comments on the introduction of Casitas B lymphoma-b (Cbl-b) and its role in immune cell regulation. We acknowledge your valuable suggestion to enhance the introduction by elaborating on the direct link between Cbl-b's immunomodulatory functions and its relevance to Acute Myocardial Infarction (AMI) pathophysiology.

In response to your comment, we have revised the introduction as follows:

"Casitas B lymphoma-b (Cbl-b), an E3 ubiquitin ligase, is vital in the ubiquitination of proteins downstream of immune receptors, thereby inhibiting the positive signaling cascade [5]. This mechanism plays a central role in promoting peripheral T cell tolerance and suppressing autoimmunity [6], both of which are crucial in immune cell regulation. Cbl-b's immunomodulatory functions, therefore, bear a direct relevance to AMI pathophysiology.

AMI is a condition characterized by an inflammatory response, in which immune cells play a pivotal role. The activation of T cells, in particular, has been recognized as a critical step in the inflammatory response following myocardial infarction. Given Cbl-b's role in inhibiting the transcriptional activity of T cells [7] and promoting immune tolerance [8], its dysregulation may contribute to the exacerbation of AMI, thus providing a compelling rationale for focusing on this E3 ubiquitin ligase in our study.

Furthermore, research has suggested that Cbl-b may serve as a potential biomarker for AMI [9]. This is due to its high expression in immune cells and its ability to ubiquitinate and degrade receptor-activated signaling proteins [6]. These features of Cbl-b, coupled with its immunomodulatory functions, make it a promising target of study in the context of AMI pathophysiology."

We believe that this revision has strengthened the justification for the selection of Cbl-b as our study focus, as suggested by the reviewer.

Finally, we are grateful for your insightful comments and hope that our revised manuscript will meet your expectations.

3. Gap in Literature: The introduction identifies a research gap by mentioning recent findings regarding alterations in gut microbiota and metabolites in AMI patients. This section effectively hints at a novel area of exploration but would benefit from a more explicit statement of how this study intends to fill that gap. Specifically, articulating the current limitations in understanding the relationship between Cbl-b, gut microbiota, metabolites, and AMI could further clarify the study's purpose and novelty.

**RE:** We are very grateful for the opportunity to revise our manuscript and thankful for your insightful comments and suggestions that have helped us improve the quality of our work.

We agree with the reviewer that a more explicit statement regarding how our study intends to fill the research gap would add clarity to our manuscript. In this revised manuscript, we have expanded our introduction section to further highlight the current limitations in understanding the relationship between Cbl-b, gut microbiota, metabolites, and AMI. We have specifically emphasized that while recent research has revealed alterations in gut microbiota and metabolites in AMI patients, the role of Cbl-b in these modifications remains unclear. Our study, therefore, seeks to fill this gap by investigating the potential regulatory mechanisms of Cbl-b in AMI using transcriptome, 16S bacterial flora sequencing, and non-targeted metabolomics sequencing data.

We have revised the introduction as follows:

"Recent research has revealed specific changes in the gut microbiota and in vivo metabolites in AMI patients [9], providing new perspectives on AMI diagnosis and treatment. However, the role of Cbl-b in these alterations remains largely unexplored. This limitation presents a significant research gap, which this study aims to address. Based on preliminary data and literature reviews, we hypothesize that Cbl-b plays a critical role in modulating these changes. To test this hypothesis and potentially fill this research gap, we are using bioinformatic tools such as differential expression analysis and enrichment analysis to investigate transcriptome, 16S bacterial flora sequencing, and non-targeted metabolomics sequencing data from three distinct mouse treatment groups. We aim to thoroughly investigate the expression, differential changes, and functional regulation of key genes, bacterial flora, and metabolites in AMI mice following Cbl-b intervention. Through this study, we strive to reveal the potential regulatory mechanisms of Cbl-b in AMI, thereby contributing to the novelty of our study and the broader understanding of AMI. We anticipate that our findings could provide new insights into AMI pathophysiology and lead to potential new therapeutic targets. By linking our methodological approach to these potential clinical and translational impacts, we underscore the relevance and potential significance of our study in the field of AMI research. This study, therefore, not only aims to elucidate the relationship between Cbl-b, gut microbiota, metabolites, and AMI but also to potentially provide novel insights into AMI diagnosis and treatment."

We hope that these modifications adequately address the reviewer's concerns and add clarity to our study's purpose and novelty. We appreciate the opportunity to clarify these points and believe that our manuscript has significantly improved as a result.

Thank you once again for your valuable feedback.

4. Research Aims and Methodology: The final part of the introduction outlines the study's aims and the bioinformatic tools employed, which is crucial for framing the research objectives. The mention of investigating transcriptomic, microbiomic, and metabolomic changes introduces the multi-omics approach, highlighting the study's comprehensive nature. However, this section could be improved by briefly discussing the expected impact of these investigations on AMI research and potential clinical applications, thereby linking the study's objectives with its broader implications.

**RE:** Thank you for your thoughtful suggestions and constructive comments on our manuscript. We appreciate your input and have taken the time to thoroughly address each point.

In response to your comments on the Introduction section, we agree that our initial discussion could have benefited from a more explicit statement of how our study intends to fill the current research gap. We have now revised this section to highlight more clearly the limitations of current understanding in the relationship between Cbl-b, gut microbiota, metabolites, and AMI. This includes a deeper explanation of the novelty of our research in exploring these relationships.

Moreover, we agree with your suggestion to discuss the expected impact of our investigations on AMI research and potential clinical applications in the introduction. We believe that our findings could significantly contribute to the understanding of AMI and its underlying mechanisms. Therefore, we have added a paragraph in the introduction that outlines the potential implications of our study, including how our research objectives align with broader implications in the field of AMI research.

The revised text now reads:

"Recent research has revealed specific changes in the gut microbiota and in vivo metabolites in AMI patients [9], providing new perspectives on AMI diagnosis and treatment. However, the role of Cbl-b in these alterations remains largely unexplored. This limitation presents a significant research gap, which this study aims to address. Based on preliminary data and literature reviews, we hypothesize that Cbl-b plays a critical role in modulating these changes. To test this hypothesis and potentially fill this research gap, we are using bioinformatic tools such as differential expression analysis and enrichment analysis to investigate transcriptome, 16S bacterial flora sequencing, and non-targeted metabolomics sequencing data from three distinct mouse treatment groups. We aim to thoroughly investigate the expression, differential changes, and functional regulation of key genes, bacterial flora, and metabolites in AMI mice following Cbl-b intervention. Through this study, we strive to reveal the potential regulatory mechanisms of Cbl-b in AMI, thereby contributing to the novelty of our study and the broader understanding of AMI. We anticipate that our findings could provide new insights into AMI pathophysiology and lead to potential new therapeutic targets. By linking our methodological approach to these potential clinical and translational impacts, we underscore the relevance and potential significance of our study in the field of AMI research. This study, therefore, not only aims to elucidate the relationship between Cbl-b, gut microbiota, metabolites, and AMI but also to potentially provide novel insights into AMI diagnosis and treatment."

We hope that these revisions adequately address your concerns and enhance the clarity and impact of our study. We appreciate your guidance in improving our manuscript and look forward to your positive response.

Recommendations for Revision

1. Elaborate on Cbl-b and AMI Linkage: Expand on how Cbl-b's immunomodulatory roles might directly influence AMI pathogenesis or recovery, providing a more robust rationale for its investigation.

**RE:** We appreciate your positive comments on the introduction of Casitas B lymphoma-b (Cbl-b) and its role in immune cell regulation. We acknowledge your valuable suggestion to enhance the introduction by elaborating on the direct link between Cbl-b's immunomodulatory functions and its relevance to Acute Myocardial Infarction (AMI) pathophysiology.

In response to your comment, we have revised the introduction as follows:

" Casitas B lymphoma-b (Cbl-b), an E3 ubiquitin ligase, is vital in the ubiquitination of proteins downstream of immune receptors, thereby inhibiting the positive signaling cascade [5]. This mechanism plays a central role in promoting peripheral T cell tolerance and suppressing autoimmunity [6], both of which are crucial in immune cell regulation. Cbl-b's immunomodulatory functions, therefore, bear a direct relevance to AMI pathophysiology.

AMI is a condition characterized by an inflammatory response, in which immune cells play a pivotal role. The activation of T cells, in particular, has been recognized as a critical step in the inflammatory response following myocardial infarction. Given Cbl-b's role in inhibiting the transcriptional activity of T cells [7] and promoting immune tolerance [8], its dysregulation may contribute to the exacerbation of AMI, thus providing a compelling rationale for focusing on this E3 ubiquitin ligase in our study.

Furthermore, research has suggested that Cbl-b may serve as a potential biomarker for AMI [9]. This is due to its high expression in immune cells and its ability to ubiquitinate and degrade receptor-activated signaling proteins [6]. These features of Cbl-b, coupled with its immunomodulatory functions, make it a promising target of study in the context of AMI pathophysiology."

We believe that this revision has strengthened the justification for the selection of Cbl-b as our study focus, as suggested by the reviewer.

Finally, we are grateful for your insightful comments and hope that our revised manuscript will meet your expectations.

2. Clarify the Literature Gap: Make the research gap more explicit by detailing the current understanding's limitations regarding Cbl-b, gut microbiota, and metabolomics in AMI. This could involve discussing why these specific areas require further exploration and how they could impact AMI treatment strategies.

**RE:** We are very grateful for the opportunity to revise our manuscript and thankful for your insightful comments and suggestions that have helped us improve the quality of our work.

We agree with the reviewer that a more explicit statement regarding how our study intends to fill the research gap would add clarity to our manuscript. In this revised manuscript, we have expanded our introduction section to further highlight the current limitations in understanding the relationship between Cbl-b, gut microbiota, metabolites, and AMI. We have specifically emphasized that while recent research has revealed alterations in gut microbiota and metabolites in AMI patients, the role of Cbl-b in these modifications remains unclear. Our study, therefore, seeks to fill this gap by investigating the potential regulatory mechanisms of Cbl-b in AMI using transcriptome, 16S bacterial flora sequencing, and non-targeted metabolomics sequencing data.

We have revised the introduction as follows:

"Recent research has revealed specific changes in the gut microbiota and in vivo metabolites in AMI patients [9], providing new perspectives on AMI diagnosis and treatment. However, the role of Cbl-b in these alterations remains largely unexplored. This limitation presents a significant research gap, which this study aims to address. Based on preliminary data and literature reviews, we hypothesize that Cbl-b plays a critical role in modulating these changes. To test this hypothesis and potentially fill this research gap, we are using bioinformatic tools such as differential expression analysis and enrichment analysis to investigate transcriptome, 16S bacterial flora sequencing, and non-targeted metabolomics sequencing data from three distinct mouse treatment groups. We aim to thoroughly investigate the expression, differential changes, and functional regulation of key genes, bacterial flora, and metabolites in AMI mice following Cbl-b intervention. Through this study, we strive to reveal the potential regulatory mechanisms of Cbl-b in AMI, thereby contributing to the novelty of our study and the broader understanding of AMI. We anticipate that our findings could provide new insights into AMI pathophysiology and lead to potential new therapeutic targets. By linking our methodological approach to these potential clinical and translational impacts, we underscore the relevance and potential significance of our study in the field of AMI research. This study, therefore, not only aims to elucidate the relationship between Cbl-b, gut microbiota, metabolites, and AMI but also to potentially provide novel insights into AMI diagnosis and treatment."

We hope that these modifications adequately address the reviewer's concerns and add clarity to our study's purpose and novelty. We appreciate the opportunity to clarify these points and believe that our manuscript has significantly improved as a result.

Thank you once again for your valuable feedback.

3. Connect Aims to Broader Implications: Enhance the discussion of the study's aims by explicitly stating the anticipated contributions of the findings to AMI diagnostics, therapeutics, or understanding of disease mechanisms. Linking the methodological approach to potential clinical or translational impacts can underscore the study's relevance.

**RE:** Thank you once again for your valuable feedback and recommendations for improving our manuscript. We have considered your comments thoroughly and have made revisions accordingly.

To address your point about connecting our study's aims to its broader implications, we have expanded on the potential contributions of our findings to AMI diagnostics, therapeutics, and understanding of disease mechanisms in the introduction. We believe that our research could pave the way for novel diagnostic and therapeutic strategies for AMI, and we have articulated this in the revised manuscript. We have emphasized on the clinical and translational impacts of our methodological approach to underscore the relevance of our study.

As per your suggestion, we have also introduced our preliminary hypotheses based on preliminary data and literature reviews. This has provided our readers with a clearer sense of the direction of our study and its foundational assumptions. We have highlighted these hypotheses in the Introduction section and linked them back to the specific research questions we aim to answer in our study.

We believe that these revisions have strengthened the overall impact and clarity of our manuscript and we are hopeful that they meet your approval. We appreciate your guidance in enhancing our manuscript and look forward to your positive response.

Please find the revised text below:

"Recent research has revealed specific changes in the gut microbiota and in vivo metabolites in AMI patients [9], providing new perspectives on AMI diagnosis and treatment. However, the role of Cbl-b in these alterations remains largely unexplored. This limitation presents a significant research gap, which this study aims to address. Based on preliminary data and literature reviews, we hypothesize that Cbl-b plays a critical role in modulating these changes. To test this hypothesis and potentially fill this research gap, we are using bioinformatic tools such as differential expression analysis and enrichment analysis to investigate transcriptome, 16S bacterial flora sequencing, and non-targeted metabolomics sequencing data from three distinct mouse treatment groups. We aim to thoroughly investigate the expression, differential changes, and functional regulation of key genes, bacterial flora, and metabolites in AMI mice following Cbl-b intervention. Through this study, we strive to reveal the potential regulatory mechanisms of Cbl-b in AMI, thereby contributing to the novelty of our study and the broader understanding of AMI. We anticipate that our findings could provide new insights into AMI pathophysiology and lead to potential new therapeutic targets. By linking our methodological approach to these potential clinical and translational impacts, we underscore the relevance and potential significance of our study in the field of AMI research. This study, therefore, not only aims to elucidate the relationship between Cbl-b, gut microbiota, metabolites, and AMI but also to potentially provide novel insights into AMI diagnosis and treatment."

We hope that these revisions adequately address your concerns and enhance the clarity and impact of our study. We appreciate your guidance in improving our manuscript and look forward to your positive response.

4. Introduce Preliminary Hypotheses or Predictions: If applicable, briefly introduce any hypotheses or predictions based on preliminary data or literature reviews. This could provide readers with a clearer sense of direction and the study's foundational assumptions.

**RE:** Thank you once again for your valuable feedback and recommendations for improving our manuscript. We have considered your comments thoroughly and have made revisions accordingly.

To address your point about connecting our study's aims to its broader implications, we have expanded on the potential contributions of our findings to AMI diagnostics, therapeutics, and understanding of disease mechanisms in the introduction. We believe that our research could pave the way for novel diagnostic and therapeutic strategies for AMI, and we have articulated this in the revised manuscript. We have emphasized on the clinical and translational impacts of our methodological approach to underscore the relevance of our study.

As per your suggestion, we have also introduced our preliminary hypotheses based on preliminary data and literature reviews. This has provided our readers with a clearer sense of the direction of our study and its foundational assumptions. We have highlighted these hypotheses in the Introduction section and linked them back to the specific research questions we aim to answer in our study.

We believe that these revisions have strengthened the overall impact and clarity of our manuscript and we are hopeful that they meet your approval. We appreciate your guidance in enhancing our manuscript and look forward to your positive response.

Please find the revised text below:

"Recent research has revealed specific changes in the gut microbiota and in vivo metabolites in AMI patients [9], providing new perspectives on AMI diagnosis and treatment. However, the role of Cbl-b in these alterations remains largely unexplored. This limitation presents a significant research gap, which this study aims to address. Based on preliminary data and literature reviews, we hypothesize that Cbl-b plays a critical role in modulating these changes. To test this hypothesis and potentially fill this research gap, we are using bioinformatic tools such as differential expression analysis and enrichment analysis to investigate transcriptome, 16S bacterial flora sequencing, and non-targeted metabolomics sequencing data from three distinct mouse treatment groups. We aim to thoroughly investigate the expression, differential changes, and functional regulation of key genes, bacterial flora, and metabolites in AMI mice following Cbl-b intervention. Through this study, we strive to reveal the potential regulatory mechanisms of Cbl-b in AMI, thereby contributing to the novelty of our study and the broader understanding of AMI. We anticipate that our findings could provide new insights into AMI pathophysiology and lead to potential new therapeutic targets. By linking our methodological approach to these potential clinical and translational impacts, we underscore the relevance and potential significance of our study in the field of AMI research. This study, therefore, not only aims to elucidate the relationship between Cbl-b, gut microbiota, metabolites, and AMI but also to potentially provide novel insights into AMI diagnosis and treatment."

We sincerely thank you for your time and effort in reviewing our manuscript. We are confident that our revisions have adequately addressed your concerns and enhanced the overall impact of our study. We look forward to your positive response and the opportunity to contribute to the Journal of Translational Medicine.

By addressing these recommendations, the introduction can offer a more compelling and comprehensive rationale for the study, firmly positioning it within the current research landscape and clearly articulating its potential contributions to the field of cardiovascular disease.

**RE:** We sincerely appreciate the reviewers’ constructive comments and suggestions. We have made substantial revisions to our manuscript in response to these comments, which we believe have significantly improved the quality of our work.

We sincerely believe that the revised introduction now offers a more compelling and comprehensive rationale for our study. We hope that these modifications address the reviewer's concerns and make our manuscript more suitable for publication in the Journal of Translational Medicine.

Thank you once again for your time and consideration.

Review and Critical Analysis of the Materials and Methods Section

Comprehensive Evaluation:

1. Clarity and Detail: The Materials and Methods section provides a thorough overview of the experimental procedures, from the packaging of CBLB overexpression lentiviral vectors to statistical analyses. The detail provided for each step, including cell culture conditions, construction of the AMI model, staining procedures, sequencing, and bioinformatic analyses, is commendable. However, the presentation could benefit from more structured formatting, such as clearer subsection headings and bullet points for step-by-step procedures, to enhance readability.

**RE:** Thank you for providing a thorough review and constructive suggestions for our manuscript. The comments from the reviewers are valuable in improving the quality of our work. We appreciate the reviewer's recognition of the detail and depth in our Materials and Methods section. However, we would like to provide an explanation for why we choose not to follow the recommendation of restructuring our Materials and Methods section.

Regarding the reviewer's comment on enhancing the structure and formatting of the Materials and Methods section, we have considered this suggestion carefully. While we understand that clearer subheadings and bullet points or numbered lists for complex procedures could enhance readability, we believe that our current structure already provides a clear and sequential presentation of the experimental procedures.

We considered that adding bullet points or numbered lists might inadvertently obscure some details and disrupt the narrative flow of the section. The current structure is consistent with standard writing practices in scientific research and provides a balance between detail and readability, which we believe is crucial for the understanding and reproducibility of our work.

In light of these considerations, we have decided to retain the current structure of the Materials and Methods section. We appreciate the reviewer's insight and the time and effort spent reviewing our manuscript. We believe that the current format of our Materials and Methods section will effectively communicate our methods to the readers and facilitate the understanding and replication of our research.

Once again, we thank the reviewer for their constructive comments and valuable time spent on our manuscript. We look forward to hearing from you regarding the final decision.

2. Experimental Design: The experimental design, including the division of mice into control and experimental groups and the subsequent treatments, is logically laid out. The description of constructing the AMI model and the intervention strategy is detailed, providing a solid foundation for reproducibility. However, a rationale for the selection of specific methodological choices (e.g., choice of mouse model, age range, and why CBLB dosage was selected) would offer readers deeper insight into the experimental design's basis.

**RE:** We are grateful to the reviewer for their detailed feedback and constructive suggestions. In response to the reviewer's request for the rationale behind our methodological choices, we would like to clarify the following:

1. Concerning the rationale for our choice of the specific animal model, we used the widely accepted Acute Myocardial Infarction (AMI) model in C57BL/6 mice. This model is frequently employed in cardiovascular research, given the physiological, metabolic, and genetic characteristics of this mouse strain that closely mimic those of humans, thus providing a reliable simulation of human disease processes and mechanisms. Additionally, the prevalent use of the C57BL/6 strain in cardiovascular research enhances the reproducibility and comparability of our results with other studies. While this is a commonly acknowledged fact within the research community, we understand the reviewer's suggestion to elucidate this point in the manuscript. However, we believe that, given the widespread use of this model, further elaboration in the manuscript may be redundant.

2. Regarding the age range of the mice, we used 6-8 weeks old mice, which is a standard in experimental research. This age represents the peak of health in mice, with all their organs fully developed, and they remain in a healthy state for the subsequent 2-3 months of the experiment. This is a well-known fact among researchers, and we believe its inclusion in the manuscript might not be necessary.

3. As for the dosage of the CBLB viral solution (50u of 1.00E+12V.G.), we based this on a combination of our preliminary studies and previous literature. Specifically, we found this dosage to be optimal for achieving the desired therapeutic effect without causing significant adverse effects. We will provide a more detailed explanation of the rationale for this dosage selection in the revised manuscript. We'd like to elaborate further. Myocardial injections are performed by directly injecting the drug into the heart tissue using a syringe or similar device. This is typically done using an in-situ multi-point injection method, with an injection volume of 50uL per site and 3-5 injection points. The total amount of virus usually injected exceeds 10E11VG. The choice of dosage was guided by the study "Efficient and stable transduction of cardiomyocytes after intramyocardial injection or intracoronary perfusion with recombinant adeno-associated virus vectors" (PMID: 9892583).

We highly appreciate the reviewer's keen insights and believe that our responses will address the concerns raised. We trust that our clarifications will enhance the manuscript's quality and contribute to its eventual acceptance.

3. Bioinformatic and Statistical Analysis: The section detailing bioinformatic pipelines for RNA sequencing, 16S rDNA sequencing, and non-targeted metabolomics is well-articulated, showcasing the comprehensive analysis undertaken. The inclusion of software versions and parameters used is excellent for reproducibility. However, for an even more robust understanding, elaborating on the choice of specific bioinformatic tools and statistical tests in certain contexts would be beneficial. Additionally, explaining the rationale behind the selection of specific thresholds for significance in differential expression and metabolite identification would provide more insight into the analysis's stringency.

**RE:** We sincerely appreciate the reviewer's thoughtful comments and suggestions. In response to the concerns raised about our choice of specific bioinformatic tools, statistical tests, and significance thresholds, we would like to clarify as follows:

1. The bioinformatic tools utilized in our study, including differential gene expression analysis, gene ontology (GO) analysis, protein-protein interaction (PPI) network construction, and Spearman correlation analysis, are fundamental and widely used in the field. The choice of these tools was primarily based on their established reliability and general acceptance in bioinformatics analysis. While we understand the reviewer's request for a more detailed explanation of our tool selection, we believe that the widespread use and acceptance of these tools in the research community speak to their appropriateness and effectiveness.

2. The selection of statistical tests and the setting of significance thresholds in our study were made according to standard conventions in the field. These methods and thresholds are widely accepted and commonly used in similar studies. As they represent established practice, we didn't feel it necessary to justify these choices in the manuscript.

However, we acknowledge the reviewer's suggestion and will include brief explanations in the revised manuscript for the selection of specific bioinformatic tools, statistical tests, and significance thresholds to ensure clarity and reproducibility.

We are grateful for the reviewer's insightful comments, which we believe will significantly improve our manuscript's clarity and robustness.

4. Methodological Rigor: The rigorous approach to staining, sequencing, and data analysis demonstrates a high level of methodological detail. The use of control samples, quality control measures for sequencing data, and validation steps for bioinformatic models highlights the study's scientific rigor. Nevertheless, discussing potential limitations of the chosen methods or any steps taken to mitigate biases in data collection and analysis would further strengthen this section.

**RE:** We appreciate the insightful comments and constructive suggestions provided to improve our manuscript. We are grateful for the acknowledgement of the methodological rigor and the detailed approach employed in our study.

Regarding the reviewer's comment on the need for a discussion on potential limitations and biases in our experimental design, data collection, and analysis, we recognize the importance of such a discussion to the scientific rigor and validity of our study. We have incorporated a comprehensive discussion in the "Limitations" section of our manuscript, where we have acknowledged potential limitations and biases and described the steps taken to mitigate them.

In addition to the points already discussed in the manuscript, we would like to add the following points:

1. Sample Selection Bias: We acknowledge that our selection of samples may have introduced some bias into our study. To mitigate this, we employed a rigorous selection process and ensured that our samples were representative of the population in terms of age, sex, and other relevant variables.

2. Bioinformatic Analysis: We also recognize that the bioinformatics tools and databases used in our study might have some inherent biases. To minimize this, we used multiple databases and bioinformatics tools and cross-validated our results.

3. Experimental Design: While our experimental design was robust and aimed at eliminating potential sources of bias, we acknowledge that it may not be completely free of bias. We performed multiple experimental replicates and employed appropriate statistical tests to reduce the impact of any potential bias.

4. Data Collection: Despite our best efforts, there might have been some biases in data collection. We used standardized protocols and calibrated instruments to reduce such biases.

We hope that these additional discussions better address the reviewer’s concern. We appreciate the opportunity to clarify these points and believe that these changes have strengthened our manuscript.

Thank you for considering our revision.

5. Reproducibility and Transparency: The detailed description of methods and the provision of supplementary figures for critical procedures are strong points for reproducibility. However, the inclusion of accession numbers for deposited sequence data or any publicly available datasets used would enhance transparency and allow for independent validation of the findings.

**RE:** We are grateful for the insightful comments and constructive suggestions given to improve our manuscript. We appreciate the recognition of the methodological detail and reproducibility of our work.

In response to the reviewer's comment regarding transparency and data availability, we fully understand and appreciate the importance of data transparency in research reproducibility. We agree that making our datasets publicly available would allow independent validation of our findings, providing greater transparency.

However, due to certain reasons involving data sensitivity and agreements with data sources which restrict us from making all the raw data publicly available at this moment, we are unable to deposit the datasets in the public domain. We understand that this is not ideal and we apologize for any inconvenience this may cause.

Nevertheless, we are more than willing to share the relevant data and code with researchers who have a legitimate need for them. Consequently, in response to the reviewer's concern, we have revised our "Availability of data and materials" section to reflect this commitment. It now reads, “All raw data supporting the conclusions of our study, as well as any relevant code, will be made available to any researcher for the purpose of reproducing the results or replicating the procedures, upon reasonable request to the corresponding author.”

We trust that this arrangement will satisfy the requirements of the journal and its readership, while also respecting the data sharing agreements that are currently in place.

Thank you for considering our revision.

Recommendations for Revision:

1. Enhance Structure and Formatting: Improve the readability by structuring the section with clearer subheadings and possibly bullet points or numbered lists for complex procedures.

**RE:** Thank you for providing a thorough review and constructive suggestions for our manuscript. The comments from the reviewers are valuable in improving the quality of our work. We appreciate the reviewer's recognition of the detail and depth in our Materials and Methods section. However, we would like to provide an explanation for why we choose not to follow the recommendation of restructuring our Materials and Methods section.

Regarding the reviewer's comment on enhancing the structure and formatting of the Materials and Methods section, we have considered this suggestion carefully. While we understand that clearer subheadings and bullet points or numbered lists for complex procedures could enhance readability, we believe that our current structure already provides a clear and sequential presentation of the experimental procedures.

We considered that adding bullet points or numbered lists might inadvertently obscure some details and disrupt the narrative flow of the section. The current structure is consistent with standard writing practices in scientific research and provides a balance between detail and readability, which we believe is crucial for the understanding and reproducibility of our work.

In light of these considerations, we have decided to retain the current structure of the Materials and Methods section. We appreciate the reviewer's insight and the time and effort spent reviewing our manuscript. We believe that the current format of our Materials and Methods section will effectively communicate our methods to the readers and facilitate the understanding and replication of our research.

Once again, we thank the reviewer for their constructive comments and valuable time spent on our manuscript. We look forward to hearing from you regarding the final decision.

2. Provide Rationale for Methodological Choices: Include explanations for the selection of specific animal models, age ranges, dosages, and methodological approaches to offer readers a deeper understanding of the experimental design.

**RE:** We are grateful to the reviewer for their detailed feedback and constructive suggestions. In response to the reviewer's request for the rationale behind our methodological choices, we would like to clarify the following:

1. Concerning the rationale for our choice of the specific animal model, we used the widely accepted Acute Myocardial Infarction (AMI) model in C57BL/6 mice. This model is frequently employed in cardiovascular research, given the physiological, metabolic, and genetic characteristics of this mouse strain that closely mimic those of humans, thus providing a reliable simulation of human disease processes and mechanisms. Additionally, the prevalent use of the C57BL/6 strain in cardiovascular research enhances the reproducibility and comparability of our results with other studies. While this is a commonly acknowledged fact within the research community, we understand the reviewer's suggestion to elucidate this point in the manuscript. However, we believe that, given the widespread use of this model, further elaboration in the manuscript may be redundant.

2. Regarding the age range of the mice, we used 6-8 weeks old mice, which is a standard in experimental research. This age represents the peak of health in mice, with all their organs fully developed, and they remain in a healthy state for the subsequent 2-3 months of the experiment. This is a well-known fact among researchers, and we believe its inclusion in the manuscript might not be necessary.

3. As for the dosage of the CBLB viral solution (50u of 1.00E+12V.G.), we based this on a combination of our preliminary studies and previous literature. Specifically, we found this dosage to be optimal for achieving the desired therapeutic effect without causing significant adverse effects. We will provide a more detailed explanation of the rationale for this dosage selection in the revised manuscript. We'd like to elaborate further. Myocardial injections are performed by directly injecting the drug into the heart tissue using a syringe or similar device. This is typically done using an in-situ multi-point injection method, with an injection volume of 50uL per site and 3-5 injection points. The total amount of virus usually injected exceeds 10E11VG. The choice of dosage was guided by the study "Efficient and stable transduction of cardiomyocytes after intramyocardial injection or intracoronary perfusion with recombinant adeno-associated virus vectors" (PMID: 9892583).

We highly appreciate the reviewer's keen insights and believe that our responses will address the concerns raised. We trust that our clarifications will enhance the manuscript's quality and contribute to its eventual acceptance.

3. Elaborate on Bioinformatic and Statistical Rationales: Detail the reasons behind the choice of specific bioinformatic tools, statistical tests, and significance thresholds to clarify the analysis's stringency and objectives.

**RE:** We sincerely appreciate the reviewer's thoughtful comments and suggestions. In response to the concerns raised about our choice of specific bioinformatic tools, statistical tests, and significance thresholds, we would like to clarify as follows:

1. The bioinformatic tools utilized in our study, including differential gene expression analysis, gene ontology (GO) analysis, protein-protein interaction (PPI) network construction, and Spearman correlation analysis, are fundamental and widely used in the field. The choice of these tools was primarily based on their established reliability and general acceptance in bioinformatics analysis. While we understand the reviewer's request for a more detailed explanation of our tool selection, we believe that the widespread use and acceptance of these tools in the research community speak to their appropriateness and effectiveness.

2. The selection of statistical tests and the setting of significance thresholds in our study were made according to standard conventions in the field. These methods and thresholds are widely accepted and commonly used in similar studies. As they represent established practice, we didn't feel it necessary to justify these choices in the manuscript.

However, we acknowledge the reviewer's suggestion and will include brief explanations in the revised manuscript for the selection of specific bioinformatic tools, statistical tests, and significance thresholds to ensure clarity and reproducibility.

We are grateful for the reviewer's insightful comments, which we believe will significantly improve our manuscript's clarity and robustness.

4. Discuss Methodological Limitations and Biases: Address potential limitations and biases in the experimental design, data collection, and analysis, along with any measures taken to mitigate these issues.

**RE:** We appreciate the insightful comments and constructive suggestions provided to improve our manuscript. We are grateful for the acknowledgement of the methodological rigor and the detailed approach employed in our study.

Regarding the reviewer's comment on the need for a discussion on potential limitations and biases in our experimental design, data collection, and analysis, we recognize the importance of such a discussion to the scientific rigor and validity of our study. We have incorporated a comprehensive discussion in the "Limitations" section of our manuscript, where we have acknowledged potential limitations and biases and described the steps taken to mitigate them.

In addition to the points already discussed in the manuscript, we would like to add the following points:

1. Sample Selection Bias: We acknowledge that our selection of samples may have introduced some bias into our study. To mitigate this, we employed a rigorous selection process and ensured that our samples were representative of the population in terms of age, sex, and other relevant variables.

2. Bioinformatic Analysis: We also recognize that the bioinformatics tools and databases used in our study might have some inherent biases. To minimize this, we used multiple databases and bioinformatics tools and cross-validated our results.

3. Experimental Design: While our experimental design was robust and aimed at eliminating potential sources of bias, we acknowledge that it may not be completely free of bias. We performed multiple experimental replicates and employed appropriate statistical tests to reduce the impact of any potential bias.

4. Data Collection: Despite our best efforts, there might have been some biases in data collection. We used standardized protocols and calibrated instruments to reduce such biases.

We hope that these additional discussions better address the reviewer’s concern. We appreciate the opportunity to clarify these points and believe that these changes have strengthened our manuscript.

Thank you for considering our revision.

5. Enhance Transparency with Data Availability: Ensure that sequence data and any other relevant datasets are deposited in publicly accessible databases, and include accession numbers or links within the manuscript.

**RE:** We are grateful for the insightful comments and constructive suggestions given to improve our manuscript. We appreciate the recognition of the methodological detail and reproducibility of our work.

In response to the reviewer's comment regarding transparency and data availability, we fully understand and appreciate the importance of data transparency in research reproducibility. We agree that making our datasets publicly available would allow independent validation of our findings, providing greater transparency.

However, due to certain reasons involving data sensitivity and agreements with data sources which restrict us from making all the raw data publicly available at this moment, we are unable to deposit the datasets in the public domain. We understand that this is not ideal and we apologize for any inconvenience this may cause.

Nevertheless, we are more than willing to share the relevant data and code with researchers who have a legitimate need for them. Consequently, in response to the reviewer's concern, we have revised our "Availability of data and materials" section to reflect this commitment. It now reads, “All raw data supporting the conclusions of our study, as well as any relevant code, will be made available to any researcher for the purpose of reproducing the results or replicating the procedures, upon reasonable request to the corresponding author.”

We trust that this arrangement will satisfy the requirements of the journal and its readership, while also respecting the data sharing agreements that are currently in place.

Thank you for considering our revision.

By addressing these recommendations, the Materials and Methods section could provide a clearer, more comprehensive foundation for understanding the experimental design, execution, and analysis, ultimately enhancing the study's reproducibility and transparency.

Comments about the Results

The "Results" section presents comprehensive findings on the effects of CBLB intervention in acute myocardial infarction (AMI) mice, spanning myocardial infarction reduction, microbial community richness alterations, key microbiota identification, metabolite changes, differential gene expression analysis, hub genes selection, and multi-omics correlation analysis. The detailed multi-omics approach reveals significant insights into the biological mechanisms of AMI and the potential therapeutic role of CBLB, showcasing a meticulous integration of data across transcriptomics, microbiomics, and metabolomics. The findings underscore the complexity of AMI pathology and suggest that CBLB intervention could modulate cardiac health, gut microbiota composition, and metabolic profiles in beneficial ways.

**RE:** We would like to express our sincere gratitude for the time and effort you have dedicated to reviewing our manuscript. We appreciate your positive feedback on the comprehensive nature of our findings and the meticulous integration of data across transcriptomics, microbiomics, and metabolomics presented in the "Results" section.

Your recognition of our detailed multi-omics approach and its contribution to revealing significant insights into the biological mechanisms of AMI and the potential therapeutic role of CBLB is encouraging. We are pleased that our efforts to underscore the complexity of AMI pathology and suggest that CBLB intervention could modulate cardiac health, gut microbiota composition, and metabolic profiles in beneficial ways have been well-received.

We agree with your comment and believe that our comprehensive findings provide a robust exploration of the effects of CBLB intervention in acute myocardial infarction (AMI) mice. It further motivates us to continue our research in this direction.

Once again, we thank you for your positive remarks and constructive suggestions. They indeed enhance the quality of our research work.

Comprehensive Review and Critical Analysis of the Results Part

1. Data Validation

The manuscript outlines rigorous methodologies for data acquisition, notably in the application of myocardial infarction models, microbial community analysis, and multi-omics approaches. However, the validation of the experimental data appears to be reliant predominantly on statistical significance without comprehensive methodological cross-validation or external dataset validation. It is recommended that the authors incorporate additional layers of validation, such as technical replicates for omics data and validation cohorts for biological findings, to enhance the robustness and reliability of the conclusions drawn.

**RE:** We are grateful for your thoughtful comments and constructive suggestions on our manuscript. We agree that incorporating additional layers of validation for our data, such as methodological cross-validation, technical replicates for omics data, and validation cohorts for biological findings, would enhance the robustness and reliability of our conclusions.

Your suggestion underscores the importance of data validation in scientific research, a point we fully acknowledge. We understand that robust data validation is key to ensuring the reliability of our findings and conclusions, and we appreciate your guidance on this matter.

However, as you have pointed out, the implementation of cross-validation and external dataset validation requires the collection of appropriate datasets. At present, we have not been able to locate relevant sequencing data (gene sequencing, microbiota, and metabolites) based on CBLB overexpression in mice, humans, or other species with AMI in any publicly accessible databases. This makes the process of acquiring suitable datasets for validation a considerable challenge at this point.

Nevertheless, we acknowledge the importance of this validation step and will continue to monitor available databases for suitable datasets. As we acquire these datasets, we will incorporate them into our validation process to enhance the robustness and reliability of our conclusions.

Once again, we thank you for your valuable feedback and look forward to further improving our manuscript with your guidance.

2. Data Integration

The integration of multi-omics data (genomics, microbiomics, and metabolomics) is commendable, offering a holistic view of the impacts of CBLB intervention on myocardial infarction. Yet, the integration strategy seems to be primarily descriptive. For a more profound understanding, advanced computational models that can infer causal relationships or network-based approaches to elucidate the interplay between different biological layers would enrich the analysis. The authors might explore machine learning or systems biology methodologies to integrate these data layers more effectively.

**RE:** We appreciate your insightful comments and recommendations on our manuscript. We acknowledge the importance of employing advanced computational models and network-based approaches for a more profound understanding of our multi-omics data integration.

Your suggestion to use machine learning or systems biology methodologies to more effectively integrate these data layers is indeed a valuable one. These advanced techniques would allow us to infer causal relationships and elucidate the interplay between different biological layers, enriching our analysis and providing a more comprehensive view of the impacts of CBLB intervention on myocardial infarction.

However, as you correctly pointed out, our current integration strategy is primarily descriptive. The implementation of machine learning or systems biology methodologies is a complex task that requires a deep understanding and expertise in these fields, which we are currently in the process of acquiring. Thus, at this stage, we may not be able to incorporate these advanced methodologies into our analysis.

We also want to mention that this study is a preliminary investigation in our series of research. We plan to further delve into these data once we have fully mastered the advanced techniques you recommended, and we are currently in the research design phase for these future studies.

We highly value your suggestions and will continue to strive towards enhancing our research methodology and data analysis. We are committed to improving the quality of our work and look forward to your continued guidance.

3. Strengths and Significance

This study's strengths lie in its comprehensive multi-omics approach, revealing the multifaceted effects of CBLB intervention on AMI mice models. The identification of key microbiota, metabolites, and genes associated with CBLB intervention offers valuable insights into the molecular mechanisms underlying myocardial infarction recovery. These findings underscore the potential of targeting the gut microbiota and metabolic pathways as therapeutic strategies for myocardial infarction, marking a significant advancement in the field.

**RE:** We sincerely appreciate your kind words and positive feedback on our study. We are glad to know that our manuscript's comprehensive multi-omics approach, which reveals the multifaceted effects of CBLB intervention on AMI mice models, has been well-received.

Your recognition of the strengths of our study and the significance of our findings is indeed encouraging. We are pleased that our efforts to identify key microbiota, metabolites, and genes associated with CBLB intervention have been acknowledged. We agree with your observation that these findings offer valuable insights into the molecular mechanisms underlying myocardial infarction recovery.

We are particularly gratified by your comment that our findings underscore the potential of targeting the gut microbiota and metabolic pathways as therapeutic strategies for myocardial infarction, marking a significant advancement in the field. Your recognition further motivates us to continue our research in this critical area.

Once again, we thank you for your valuable feedback, which greatly enhances the quality of our work. We look forward to your continued guidance.

4. Novelty

The investigation into the effects of CBLB on myocardial infarction through multi-omics analysis represents a novel contribution to the understanding of heart disease mechanisms and recovery. Particularly, the focus on gut microbiota as a mediator of therapeutic effects in AMI treatment opens new avenues for research and therapy development. However, the novelty would be further enhanced by elucidating the mechanistic pathways through which CBLB affects myocardial infarction outcomes, which remains to be fully addressed in this study.

**RE:** We appreciate your constructive comments and your recognition of the novelty of our study. We agree that elucidating the mechanistic pathways through which CBLB affects myocardial infarction outcomes would greatly enhance the novelty and impact of our work.

Your feedback underscores the necessity and importance of understanding the precise mechanisms that underpin the observed effects. We understand that this is a complex task and acknowledge that our current work does not fully address these mechanistic pathways. We agree that this is a key area that needs to be more thoroughly explored in our study.

In response to your feedback, we have supplemented our discussion section with functional enrichment analysis results for differentially expressed genes (DEGs) between the CBLB overexpression+AMI group and the AMI group. We hope that this addition will provide direction and inspiration for our future work and for other researchers interested in this study. The specific descriptions are as follows: Despite not yet completely unraveling the specific mechanism of CBLB in AMI regulation, it remains a limitation of our current study. However, Gene Ontology (GO) enrichment analysis of the 143 DEGs between group B and group C (Supplementary Table 7) has paved the way for fresh insights and future mechanistic studies. Analysis of GO-BP items reveals a close relationship between these DEGs and factors such as ventricular myocardial tissue, ventricle, cardiac morphogenesis, ventricular muscle tissue development, and adult heart development. Concurrently, significant enrichment was also observed in the ‘negative regulation of vascular endothelial growth factor receptor signaling pathway’ and ‘coronary artery morphogenesis’. Importantly, the involvement of immunology-related biological processes, including the ‘immunological memory process’, ‘natural killer cell proliferation’, and ‘humoral immune response’, is also observed. Consequently, we hypothesize that CBLB intervention might effectively attenuate the augmentation of collagen fibers in group C mice by modulating the signaling pathways associated with these biological processes. In subsequent research, we aim to delve deeper into the exact mechanism of CBLB and explore its potential therapeutic application in treating AMI.

We sincerely thank you for your valuable suggestions. Your insights will greatly inform our ongoing research efforts and help us improve the quality of our work.

5. Significance

The clinical significance of this research lies in its potential to pave the way for novel therapeutic interventions targeting the microbiome and metabolic pathways in myocardial infarction treatment. By demonstrating the efficacy of CBLB intervention in modulating key microbiota and metabolites, the study offers promising avenues for developing targeted therapies that could improve patient outcomes in myocardial infarction.

**RE:** We sincerely appreciate your positive feedback and recognition of the clinical significance of our study. We are encouraged by your acknowledgment of the potential of our research to guide the development of novel therapeutic interventions for myocardial infarction treatment.

Your comments highlight the promise of our findings in demonstrating the efficacy of CBLB intervention in modulating key microbiota and metabolites. We are pleased that our research has been recognized as providing promising avenues for improving patient outcomes in myocardial infarction through the development of targeted therapies.

We wholeheartedly agree with your perspective on the significance of our study. It affirms our belief in the potential of microbiome and metabolic pathway targeting as a novel approach to myocardial infarction treatment. This feedback further motivates us to continue our research in this critical area.

Once again, we thank you for your valuable feedback, which provides us with the impetus to further our research and strive for excellence.

6. Drawbacks

A notable drawback is the study's limited exploration of the direct mechanistic pathways connecting CBLB intervention with observed outcomes in myocardial infarction. Additionally, the study relies heavily on animal models, raising questions about the translatability of findings to human patients. Further investigation into the human applicability of these results is necessary.

**RE:** We are grateful for your insightful comments and for pointing out the drawbacks of our study. We acknowledge the importance and necessity of your suggestions, particularly in regard to exploring the direct mechanistic pathways and extending our research to human patients.

We understand the limitation of our study in terms of the limited investigation into the direct mechanistic pathways connecting CBLB intervention with observed outcomes in myocardial infarction. We concur that further investigation in this area would undoubtedly strengthen our study and appreciate your suggestion in this regard.

Regarding the translation of findings from animal models to human patients, we agree that this is a crucial step in confirming the applicability of our results. However, due to financial constraints, difficulties in human sample collection, and ethical considerations, we are currently unable to carry out studies in human or other animal models.

While we are aware of these limitations, we believe that our study still provides valuable insights into the therapeutic potential of CBLB intervention in myocardial infarction. We are actively seeking additional funding and exploring collaborations to overcome these challenges, and we hope to address these issues in our future work.

Once again, we appreciate your valuable feedback, which will greatly help us improve our research. We look forward to your continued guidance and support.

7. Best Solution

To address these drawbacks, the authors could consider extending their study to include translational research components, such as investigating the effects of CBLB on human-derived cardiac cells or exploring the feasibility of clinical trials. Incorporating mechanistic studies that elucidate the direct pathways through which CBLB exerts its effects would also strengthen the study's impact.

**RE:** We greatly appreciate your constructive suggestions on how to address the drawbacks in our study. Your recommendations to extend our study to include translational research components and to incorporate mechanistic studies are indeed valuable and necessary, and we acknowledge the importance of these points.

Given the financial constraints and the difficulties in human sample collection, we are currently unable to extend our study to human-derived cardiac cells or explore the feasibility of clinical trials. We hope that you understand these limitations are temporary and we are actively seeking solutions to overcome these challenges.

However, we have taken your comments seriously and have begun to focus our future research on investigating the impact of CBLB on human-derived cardiac cells. We are also planning to incorporate mechanistic studies that elucidate the direct pathways through which CBLB exerts its effects, as we agree that this would significantly strengthen the impact of our study.

We would like to express our gratitude for your insightful comments, which will greatly help us improve our research. We look forward to your continued guidance and support.

8. Reproducibility

The detailed description of experimental procedures and statistical analyses enhances the reproducibility of the study. Nevertheless, making raw data and code available in public repositories would further solidify the study's reproducibility, allowing other researchers to validate and build upon the work presented.

**RE:** We are grateful for the insightful comments and constructive suggestions given to improve our manuscript. We appreciate the recognition of the methodological detail and reproducibility of our work.

In response to the reviewer's comment regarding transparency and data availability, we fully understand and appreciate the importance of data transparency in research reproducibility. We agree that making our datasets publicly available would allow independent validation of our findings, providing greater transparency.

However, due to certain reasons involving data sensitivity and agreements with data sources which restrict us from making all the raw data publicly available at this moment, we are unable to deposit the datasets in the public domain. We understand that this is not ideal and we apologize for any inconvenience this may cause.

Nevertheless, we are more than willing to share the relevant data and code with researchers who have a legitimate need for them. Consequently, in response to the reviewer's concern, we have revised our "Availability of data and materials" section to reflect this commitment. It now reads, “All raw data supporting the conclusions of our study, as well as any relevant code, will be made available to any researcher for the purpose of reproducing the results or replicating the procedures, upon reasonable request to the corresponding author.”

We trust that this arrangement will satisfy the requirements of the journal and its readership, while also respecting the data sharing agreements that are currently in place.

Thank you for considering our revision.

9. Potentially Further and Futuristic Researches

Future research could explore the therapeutic potential of CBLB in a clinical setting, assessing its safety and efficacy in human patients. Additionally, investigations into the specific molecular mechanisms by which CBLB influences the gut microbiome and metabolic pathways would provide deeper insights into its therapeutic potential. Expanding the scope to include other cardiovascular diseases could also reveal broader applications for CBLB intervention.

**RE:** We sincerely appreciate your insightful comments and suggestions for future directions of our research. We completely agree with your view that exploring the therapeutic potential of CBLB in a clinical setting and investigating the specific molecular mechanisms of CBLB's influence on the gut microbiome and metabolic pathways are important for gaining deeper insights into the therapeutic potential of CBLB.

Indeed, assessing the safety and efficacy of CBLB in human patients is the ultimate goal of our research, and we are actively seeking ways to translate our findings into clinical practice. However, as previously mentioned, due to current financial constraints and difficulties in human sample collection, we are unable to immediately carry out these clinical studies.

Your suggestion to expand the scope to include other cardiovascular diseases is also very insightful. We will certainly consider this in our future research planning. We believe that this approach could reveal broader applications for CBLB intervention, thus increasing the impact and relevance of our work.

We are grateful for your thoughtful recommendations, which provide valuable guidance for our future research. We look forward to continuing to improve our work with your expert advice.

10. Conclusions and Recommendations

This study presents a significant advance in our understanding of the therapeutic potential of CBLB in myocardial infarction, particularly through the modulation of the gut microbiota and metabolic pathways. While the findings offer promising avenues for novel treatments, further research is needed to translate these findings into clinical practice. The authors are encouraged to pursue translational studies and mechanistic investigations to fully leverage the therapeutic potential of CBLB intervention in myocardial infarction and beyond.

**RE:** We sincerely appreciate your thoughtful feedback and recognition of the significance of our study. We are encouraged by your acknowledgment of our contribution to the understanding of the therapeutic potential of CBLB in myocardial infarction, particularly through the modulation of the gut microbiota and metabolic pathways.

We wholeheartedly agree with your assertion that while our findings offer promising avenues for novel treatments, further research is needed to translate these findings into clinical practice. We are fully aware of this need, and we are actively seeking resources and collaboration opportunities to enable us to pursue translational studies.

Your recommendation to conduct mechanistic investigations to fully leverage the therapeutic potential of CBLB intervention is well received. We believe this is a crucial step to strengthen the impact of our study, and we are planning to incorporate this aspect into our future research.

In conclusion, we are greatly thankful for your valuable suggestions, which provide us with clear guidance for our future research. We look forward to your continued guidance and support in our endeavors.

Critical Review of the Discussion Section

1. Data Validation

The authors conscientiously acknowledge the limitations inherent to their study, particularly concerning the translatability of findings from a mouse model to human physiology. This level of transparency is commendable. However, the discussion would benefit from a more detailed examination of the methodologies employed for data validation, especially in light of the complex interplay between gut microbiota, metabolites, and gene expression. A discussion on the use of orthogonal methods or independent datasets to validate key findings would have significantly strengthened the confidence in these results.

**RE:** We would like to express our sincere gratitude for your thoughtful feedback and recognition of our transparency in acknowledging the limitations of our study. We value your suggestion about the need for a more detailed examination of the methodologies used for data validation.

We completely agree that discussing the use of orthogonal methods or independent datasets for validation would significantly strengthen the confidence in the results. However, due to financial constraints and difficulties in human sample collection, we are currently unable to carry out these validation studies in human or other animal models. We hope that you understand these limitations are temporary and we are actively seeking solutions to overcome these challenges.

Nevertheless, we are fully aware of the importance of rigorous data validation, especially given the complex interplay between gut microbiota, metabolites, and gene expression. We appreciate your suggestion and assure you that it will be a key focus in our future research. We believe that pursuing these validation methodologies will greatly enhance the robustness and translatability of our findings.

We would like to thank you again for your valuable comments, which will greatly guide our future work. We look forward to your continued guidance and support.

2. Data Integration

The authors have adeptly integrated data from multi-omics approaches to elucidate the multifaceted role of CBLB in AMI. This comprehensive integration is a significant strength of the study. Nonetheless, the discussion could be enriched by a more in-depth analysis of how these disparate data streams converge to inform the mechanistic understanding of CBLB's effects. Specifically, a systematic approach to integrating these data types to model the biological networks involved could offer deeper insights and potentially uncover novel therapeutic targets.

**RE:** We greatly appreciate your recognition of our efforts in adeptly integrating data from multi-omics approaches to elucidate the multifaceted role of CBLB in AMI. Your feedback underscores the importance of our comprehensive integration and its role as a significant strength of our study.

We acknowledge your suggestion for a more in-depth analysis of how these disparate data streams converge to inform the mechanistic understanding of CBLB's effects. We agree that a systematic approach to integrating these data types to model the biological networks involved could offer deeper insights and potentially uncover novel therapeutic targets.

However, we would like to candidly admit that while we recognize the necessity of such an approach, we are currently in the process of mastering the technical skills required to implement it. We are actively seeking to enhance our understanding and application of these techniques to improve our analysis plan.

In the meantime, we assure you that your suggestion has been taken into serious consideration and will be a key focus of our subsequent research. We are committed to continuously improving our research and believe that this approach will significantly enrich our future studies.

Once again, we are grateful for your valuable feedback, which provides us with clear guidance for our future research. We look forward to your continued guidance and support.

3. Strengths and Significance

The study's significant strengths include its innovative multi-omics approach and the novel insights it provides into the role of CBLB in AMI. The identification of changes in gut microbiota composition, key metabolites, and gene expression patterns associated with CBLB overexpression in AMI mice models is particularly noteworthy. These findings underscore the potential of targeting the gut microbiome and metabolic pathways in myocardial infarction treatment, offering new avenues for research and therapy development.

**RE:** We sincerely appreciate your recognition of the strengths and significance of our study, specifically our innovative multi-omics approach and the novel insights provided into the role of CBLB in AMI. We are also grateful for your acknowledgment of the noteworthy findings related to changes in gut microbiota composition, key metabolites, and gene expression patterns associated with CBLB overexpression in AMI mouse models.

We agree wholeheartedly with your assessment that our findings underscore the potential of targeting the gut microbiome and metabolic pathways in myocardial infarction treatment, and offer new avenues for research and therapy development. Your comments affirm the value and impact of our research, and serve as motivation for us to continue our work in this critical area.

Your feedback provides us with the impetus to further our research efforts and strive for excellence. We are committed to continuing our efforts to explore the therapeutic potential of CBLB and its impact on AMI treatment.

Once again, we sincerely thank you for your constructive and encouraging comments. We look forward to your continued guidance and support.

4. Novelty

The novelty of the study lies in its comprehensive examination of the effects of CBLB on myocardial infarction through a multi-omics lens. The discussion adeptly highlights how these findings contribute new knowledge to the field, particularly regarding the systemic effects of myocardial infarction on body weight, the potential of CBLB in preventing myocardial fibrosis, and the modulation of gut microbiota. Further elaboration on how these findings diverge from or build upon existing literature would enhance the discussion of novelty.

**RE:** We sincerely appreciate the insightful comments on our manuscript and the recognition of the novelty of our study. We fully agree with the reviewer's suggestion that further elaboration on how our findings diverge from or build upon existing literature would enhance the discussion of novelty.

We would like to clarify that we have indeed tried to emphasize the novelty in our manuscript. For instance, we discussed that our study provides "an innovative observation that offers fresh insights into the systemic effects of myocardial infarction. The precise causes of weight loss, as well as the correlation between weight fluctuations and myocardial infarction severity, warrant further investigation." We also mentioned that "this novel insight broadens our comprehension of myocardial infarction treatment. Nonetheless, a more in-depth investigation into the specific mechanisms driving these outcomes is necessary."

We acknowledge, however, that the discussion on the five key microbial groups, 11 key metabolites, and 9 key genes could be further enriched. In this context, we failed to adequately present how our findings diverge from or build upon the existing literature. We appreciate the reviewer pointing out this oversight and assure that we will rectify this in the revised manuscript.

Thank you again for your constructive feedback, which will undoubtedly improve the quality of our manuscript. We look forward to the opportunity to further refine our work.

5. Significance

The significance of the study is well articulated, especially in highlighting the potential therapeutic implications of CBLB intervention. The discussion effectively communicates the relevance of the findings for future therapeutic strategies, albeit with a cautionary note regarding the translational gap between mouse models and human disease. A more explicit discussion on the clinical translation pathway, including potential challenges and strategies to overcome them, would be valuable.

**RE:** We sincerely appreciate your constructive comments and suggestions on our manuscript. We value your feedback highlighting the significance of our study and the potential therapeutic implications of CBLB intervention. We are particularly grateful for your suggestion to provide a more explicit discussion on the clinical translation pathway, including potential challenges and strategies to overcome them.

In response to this suggestion, we have revised the Discussion section to elaborate on the potential clinical translation of our findings. We have included a section discussing the challenges that might be encountered in translating these findings from mouse models to human disease, such as the differences in physiology and disease progression between mice and humans.

We believe that this addition has greatly enhanced the overall quality of our manuscript by providing a more comprehensive discussion on the potential clinical implications of our research. We hope that this revised version of our manuscript will meet your approval.

Thank you for your time and consideration.

6. Drawbacks

The discussion candidly addresses several key limitations, including the use of a mouse model and the correlative nature of the findings. While these acknowledgments are important, a deeper exploration of the potential biases and confounding factors inherent in the study design would provide a more comprehensive understanding of the limitations. Additionally, a discussion on the limitations of the metabolites and genes identified, in terms of their specificity and role in AMI, would be insightful.

**RE:** We would like to express our gratitude to the reviewer for their valuable suggestions on our manuscript. We agree with the reviewer that a more comprehensive understanding of the potential biases, confounding factors, and limitations of our study is necessary, and a deeper exploration of the metabolites and genes identified in terms of their specificity and role in AMI would be insightful.

Addressing the first point raised by the reviewer, we have revised our manuscript to offer a more in-depth discussion of the biases and confounding factors inherent in our study design. We have acknowledged that our selection of samples may have introduced some bias into our research. To mitigate this, we employed a rigorous selection process, ensuring our samples were representative of the population in terms of age, sex, and other pertinent variables. Moreover, we have recognized that the bioinformatics tools and databases used might have inherent biases. Despite the robustness of our experimental design and our efforts to eliminate potential sources of bias, we acknowledge that some bias may still exist. As such, we performed multiple experimental replicates and used appropriate statistical tests to minimize the impact of any potential bias.

Concerning the second point, we have expanded our discussion on the limitations of the metabolites and genes identified in terms of their specificity and role in AMI. We have highlighted that these identified metabolites and genes, while statistically correlated with AMI in our study, may not be exclusive to AMI. We have also noted that other diseases or physiological conditions may influence their levels, thereby introducing confounding factors. Furthermore, we have emphasized that the role of these metabolites and genes in AMI is not fully understood and that further studies are required to confirm causal relationships and mechanisms of action.

We hope that our revisions have adequately addressed the reviewer's concerns, and we believe that these changes have significantly improved the quality and comprehensibility of our study. We thank the reviewer for their critical and insightful comments, which have helped enhance our manuscript.

7. Best Solution

The best solution to addressing the limitations mentioned would involve the incorporation of translational research elements, such as validation studies in human samples or clinical trials, as well as mechanistic studies to elucidate the direct pathways through which CBLB exerts its effects. The authors could also consider expanding their analytical frameworks to include computational modeling or network analysis to better integrate and understand the multi-omics data.

**RE:** We would like to express our sincere gratitude to the reviewer for their insightful comments and constructive suggestions on our manuscript. We appreciate the importance and necessity of their recommendations and agree that they would significantly enhance the comprehensibility and applicability of our study.

In relation to the reviewer's suggestion to incorporate translational research elements such as validation studies in human samples or clinical trials and mechanistic studies to elucidate the direct pathways through which CBLB exerts its effects, we fully agree with this perspective. Indeed, it would be ideal to conduct such studies to corroborate our findings and potentially translate them into clinical applications. However, we regret to inform that due to limited funding and insufficient sample volume, we are currently unable to undertake these types of studies. Nevertheless, we acknowledge the importance and impact these studies could have on our research and will certainly consider incorporating them in our future research plans whenever possible.

Regarding the recommendation to expand our analytical frameworks to include computational modeling or network analysis for better integration and understanding of the multi-omics data, we concur that this approach would indeed provide a more comprehensive view of our data. However, at present, our team does not yet have adequate proficiency in computational modeling or network analysis techniques. We acknowledge the importance of these techniques in modern biological research and they are indeed a primary focus in our ongoing skill development plan. We hope to incorporate these techniques in our future studies to provide a more holistic understanding of our research.

We sincerely appreciate the reviewer's critical and insightful comments, which have certainly provided us with valuable guidance for improving our work. We believe that our study, even with its current limitations, contributes valuable knowledge to the field and we will strive to incorporate these suggestions in our future research.

Thank you once again for your time and expertise.

8. Reproducibility

The discussion could benefit from a more detailed commentary on the steps taken to ensure the reproducibility of the study, including data availability, code sharing, and the use of standardized protocols. This would not only bolster the study's credibility but also facilitate future work by other researchers in the field.

**RE:** We are grateful for the insightful comments and constructive suggestions given to improve our manuscript. We appreciate the recognition of the methodological detail and reproducibility of our work.

In response to the reviewer's comment regarding transparency and data availability, we fully understand and appreciate the importance of data transparency in research reproducibility. We agree that making our datasets publicly available would allow independent validation of our findings, providing greater transparency.

However, due to certain reasons involving data sensitivity and agreements with data sources which restrict us from making all the raw data publicly available at this moment, we are unable to deposit the datasets in the public domain. We understand that this is not ideal and we apologize for any inconvenience this may cause.

Nevertheless, we are more than willing to share the relevant data and code with researchers who have a legitimate need for them. Consequently, in response to the reviewer's concern, we have revised our "Availability of data and materials" section to reflect this commitment. It now reads, “All raw data supporting the conclusions of our study, as well as any relevant code, will be made available to any researcher for the purpose of reproducing the results or replicating the procedures, upon reasonable request to the corresponding author.”

We trust that this arrangement will satisfy the requirements of the journal and its readership, while also respecting the data sharing agreements that are currently in place.

Thank you for considering our revision.

9. Potentially Further and Futuristic Researches

The authors touch upon the need for further research to validate their findings and explore the specific molecular mechanisms at play. Expanding on potential future research directions, such as the exploration of CBLB's effects in different contexts of cardiovascular disease or the examination of its role in human patients, would provide valuable guidance for the field. Additionally, discussing the potential for integrating emerging technologies or computational approaches could offer exciting perspectives on future research avenues.

**RE:** We deeply appreciate your valuable comments and suggestions on our manuscript. We acknowledge the significance of your suggestions as they indeed enrich our research and provide substantial guidance for future studies in the field.

In reference to your suggestion on "Expanding on potential future research directions, such as the exploration of CBLB's effects in different contexts of cardiovascular disease or the examination of its role in human patients, would provide valuable guidance for the field. Additionally, discussing the potential for integrating emerging technologies or computational approaches could offer exciting perspectives on future research avenues."

We would like to express our agreement with your insightful advice. In the initial version of our manuscript, we supplemented our work with "Hence, our future investigations will aim to utilize systems biology strategies to elucidate the intricate relationships among differentially expressed genes, metabolites, and microbiota alterations. This comprehensive approach could deepen our understanding of the disease mechanism." Now, in light of your recommendations, we would like to further revise our manuscript as follows:

"In addition to the systems biology strategies highlighted, we will also explore the effects of CBLB in different contexts of cardiovascular disease. This would not only validate our current findings but also open new avenues for understanding the role of CBLB in the broader spectrum of cardiovascular diseases. Furthermore, we also plan to investigate the role of CBLB in human patients to gain a real-world perspective of its implications. Lastly, we recognize the potential of integrating emerging technologies or computational approaches in our research, which we believe could provide exciting perspectives on future research avenues, and thus, we will consider incorporating these methods in our subsequent studies."

We hope these revisions adequately address your comments and suggestions. Thank you again for your constructive feedback.

10. Conclusions and Recommendations

The conclusion and recommendations offered in the discussion are well-founded, emphasizing the potential therapeutic implications of the findings and the necessity for further research. To enhance this section, the authors could offer more specific recommendations for future research priorities or clinical trial design, considering the translational potential of their findings. A more explicit call to action for the research community, outlining key questions that remain unanswered and how best to address them, would be a fitting addition to the discussion.

**RE:** We appreciate the insightful comments provided by the reviewer regarding our manuscript. We acknowledge the importance and necessity of the reviewer's suggestions, especially in enhancing the conclusions and recommendations section of our Discussion.

The reviewer's suggestion to provide more specific recommendations for future research priorities or clinical trial designs, considering the translational potential of our findings, is indeed well-taken. We concur that a more explicit call to action for the research community, outlining key questions that remain unanswered and the best ways to address them, would significantly contribute to our discussion.

Following your valuable suggestion, we have revised and supplemented this section as follows: "While we have presented correlative findings, causal relationships and mechanisms of action need to be confirmed through further studies. In addition to the systems biology strategies highlighted, we will also explore the effects of CBLB in different contexts of cardiovascular disease. This would not only validate our current findings but also open new avenues for understanding the role of CBLB in the broader spectrum of cardiovascular diseases. Furthermore, we also plan to investigate the role of CBLB in human patients to gain a real-world perspective of its implications. Lastly, we recognize the potential of integrating emerging technologies or computational approaches in our research, which we believe could provide exciting perspectives on future research avenues, and thus, we will consider incorporating these methods in our subsequent studies.”

We believe that these changes have significantly improved our manuscript by addressing the reviewer's concerns and have made our study more comprehensive and beneficial for the broader research community. We are truly grateful for the constructive criticism and guidance that have helped us refine our work.

Thank you for considering our response and the revised version of our manuscript.

More Review and Critical Analysis of the Discussion Section

Critical Evaluation:

1. Comprehensive Elucidation of Findings: The discussion comprehensively elucidates the study's findings, particularly the role of CBLB in reducing myocardial infarction severity, modulating gut microbiota, and altering metabolite and gene expression profiles in an AMI mouse model. The linkage between these observations and existing literature, including the corroborative findings from Hongjun You et al., enriches the contextual foundation of the study. However, the discussion would benefit from a more detailed comparison with conflicting or complementary studies to contextualize these findings within the broader scientific discourse.

**RE:** Thank you very much for your constructive comments and suggestions on our manuscript. We appreciate your time and effort spent on our paper, and your insightful critique which we believe will greatly improve the quality of our work.

We agree with the reviewer that the discussions would indeed benefit from a more detailed comparison with conflicting or complementary studies. However, as we have mentioned in our discussion, many of the key genes, gut microbiota and metabolites identified in our study are still not clearly linked to Acute Myocardial Infarction (AMI). The lack of a definitive relationship between these factors and AMI has constrained our ability to delve into a more detailed comparison and discussion.

We understand that our findings must be contextualized within the broader field, and we have tried our best to do so within the confines of the current knowledge. We have discussed the areas of agreement with existing literature, particularly the corroborative findings from Hongjun You et al., which enriches the contextual foundation of our study.

We hope that our revisions and clarifications meet your approval. We sincerely appreciate your constructive criticism, which has greatly improved our manuscript.

Thank you again for your time and consideration.

2. Insightful Observations and Hypotheses: The observations regarding the systemic effects of myocardial infarction on body weight and the potential of CBLB in mitigating myocardial fibrosis are insightful. These hypotheses stimulate further inquiry but require a deeper exploration of underlying mechanisms. The discussion of the role of key microbiota and metabolites introduces intriguing hypotheses about the gut-heart axis. Nonetheless, the speculative nature of these observations underscores the need for mechanistic studies to delineate causality.

**RE:** We greatly appreciate the time and effort you have invested in reviewing and evaluating our manuscript. We acknowledge the importance and necessity of the suggestions provided and have made a concerted effort to address each of them diligently.

In reference to the reviewer's comment on the need for further inquiry and a deeper exploration of underlying mechanisms, we concur with the reviewer's perspective. We understand that illuminating the mechanism pathways of how CBLB influences the prognosis of myocardial infarction is a complex task. Our current research does not allow us to pinpoint the precise mechanisms. However, to address this comment, we have supplemented our discussion section with a functional enrichment analysis of differentially expressed genes (DEGs) between the CBLC overexpression + AMI group and the AMI group. We hope that this addition will provide direction and inspiration for our future work and for other researchers interested in this study.

The reviewer's suggestion to emphasize the need for mechanistic studies to understand the causal relationships underpinning the observed effects of CBLB on myocardial infarction, gut microbiota, and metabolic profiles is indeed a significant consideration. However, our current findings do not definitively establish the relationships between these factors. To address this, we have supplemented our discussion with a section on literature-based developments to highlight the potential correlations.

We acknowledge the limitations of our study, as well as those in the broader field of research. In the description of these limitations, we candidly admit that "In terms of the specificity of the identified metabolites and genes, it should be noted that while they exhibited significant statistical correlation with AMI in our study, they may not be exclusive to AMI. Other diseases or physiological conditions may also influence their levels, which could introduce confounding factors. Furthermore, the role of these metabolites and genes in AMI is not completely understood. While we have presented correlative findings, causal relationships and mechanisms of action need to be confirmed through further studies."

We are grateful for your constructive comments and suggestions, which significantly contribute to the improvement of our manuscript. We have endeavored to address each of them adequately, and we believe that these revisions have greatly enhanced the quality of our manuscript.

3. Novelty and Contribution to the Field: The discussion articulates the novelty of the findings, particularly the potential therapeutic implications of CBLB intervention in myocardial infarction and its systemic effects. Highlighting the novel insights into the preventive and therapeutic roles of CBLB in myocardial fibrosis and the modulation of gut microbiota and metabolites adds significant value. However, delineating how these findings advance the current understanding and identifying specific gaps that the study addresses would enhance the discussion of novelty.

**RE:** We are sincerely grateful for the constructive comments and valuable time invested in the review of our manuscript. Your feedback is greatly appreciated and has provided significant guidance for the improvement of our paper.

We are encouraged by the reviewer's recognition of the novelty of our findings, particularly the potential therapeutic implications of CBLB intervention in myocardial infarction and its systemic effects. Your positive feedback reaffirms the significance and value of our research.

The reviewer's recommendation to delineate how our findings advance the current understanding and identify specific gaps that the study addresses is well-taken. We agree that such elucidation would indeed enhance the discussion of the novelty of our study. Therefore, we have revised our discussion section to explicitly state how our findings contribute to advancing existing knowledge in the field. We have also identified and highlighted the specific gaps in the literature that our study addresses.

We appreciate the reviewer's constructive comments and suggestions, which have been instrumental in enhancing the quality of our manuscript. We believe that these revisions have significantly improved the clarity and quality of our manuscript.

Thank you once again for the valuable feedback. We look forward to hearing from you.

4. Limitations and Self-Critical Analysis: The authors acknowledge key limitations, including the use of a mouse model and the correlative nature of the data, which are commendable for their transparency. However, the discussion could be strengthened by addressing potential biases, the generalizability of the findings, and the limitations of the experimental models used. A more nuanced exploration of how these limitations affect the interpretation of the results and the potential implications for clinical translation would provide a more balanced view.

**RE:** We would like to express our sincere appreciation for the insightful and valuable comments provided on our manuscript. We have carefully considered these suggestions and have made significant revisions to the manuscript accordingly.

We have taken the reviewer's recommendations to heart and have expanded the discussion on limitations to include considerations of experimental design, potential biases, and implications for clinical translation.

In response to the reviewer's comment on "Broaden Limitation Discussion", we have incorporated further detail on the limitations of our study. We now explicitly acknowledge that our selection of samples may have introduced bias into our research and that the bioinformatics tools used might also have inherent biases. We also acknowledge that despite our best efforts, some biases in data collection may still exist.

We also now discuss the limitations of our statistical models, including the assumption of certain characteristics of the data that may not always hold true in biological studies. We also acknowledge the risk of false positives due to the high-dimensional nature of the data and the reliance on existing databases in our in silico methods for data interpretation.

In addition, we have added a more nuanced discussion on the generalizability of our findings. We now point out that the identified metabolites and genes may not be exclusive to AMI and that their role in AMI is not completely understood.

We have also outlined our future research plans, including investigating the role of CBLB in different contexts of cardiovascular disease and in human patients, as well as potentially integrating emerging technologies or computational approaches in our research.

We hope this revised discussion provides a more balanced view and believe that these revisions have improved the manuscript. We appreciate the reviewer's constructive feedback, which was instrumental in enhancing the quality of our manuscript.

Thank you for considering our manuscript for publication in the Journal of Translational Medicine.

5. Future Directions and Recommendations: While the discussion hints at future research needs, a more detailed outline of specific experimental or clinical studies required to validate and extend these findings would be valuable. Recommendations for future research, including the exploration of CBLB's effects in diverse models of cardiovascular disease and the investigation into the translatability of these findings to human patients, would provide a clearer roadmap for the field.

**RE:** We sincerely thank the reviewer for their insightful comments and constructive suggestions. We appreciate the time and effort spent on reviewing our manuscript and the thought-provoking feedback that has allowed us to improve our work.

In response to the reviewer's comment on the need for a more detailed outline of specific experimental or clinical studies required to validate and extend our findings, we have incorporated this into our revised manuscript.

Firstly, as suggested, we have elaborated on our future research plans involving the investigation of CBLB's effects in diverse models of cardiovascular disease. We believe that this approach would not only serve to validate our current findings but also pave the way for a broader understanding of the role of CBLB in various cardiovascular diseases. We have reflected this in our discussion and agree that it indeed provides a clearer direction for future research.

Secondly, we have also emphasized our intention to explore the role of CBLB in human patients. We agree with the reviewer that this will provide a more realistic perspective of the implications of our research findings and strengthen the translatability of our study.

Lastly, responding to the implicit suggestion from the reviewer, we have acknowledged the potential of integrating emerging technologies and computational approaches in our research. We believe that including these methods in our future studies will enhance the robustness of our research and open up exciting new research avenues.

We trust that these revisions have adequately addressed the reviewer's concerns and have improved the quality of our manuscript. We look forward to hearing further feedback.

6. Integration with Existing Literature: The integration of the study's findings with existing literature is adeptly handled, providing a solid rationale for the study's hypotheses and conclusions. However, a more critical analysis comparing and contrasting the study's outcomes with previous conflicting or supporting studies could offer a richer context for interpreting the results.

**RE:** Thank you very much for your constructive comments and suggestions on our manuscript. We appreciate your time and effort spent on our paper, and your insightful critique which we believe will greatly improve the quality of our work.

We agree with the reviewer that the discussions would indeed benefit from a more detailed comparison with conflicting or complementary studies. However, as we have mentioned in our discussion, many of the key genes, gut microbiota and metabolites identified in our study are still not clearly linked to Acute Myocardial Infarction (AMI). The lack of a definitive relationship between these factors and AMI has constrained our ability to delve into a more detailed comparison and discussion.

We understand that our findings must be contextualized within the broader field, and we have tried our best to do so within the confines of the current knowledge. We have discussed the areas of agreement with existing literature, particularly the corroborative findings from Hongjun You et al., which enriches the contextual foundation of our study.

We hope that our revisions and clarifications meet your approval. We sincerely appreciate your constructive criticism, which has greatly improved our manuscript.

Thank you again for your time and consideration.

7. Methodological Considerations for Reproducibility: The discussion could benefit from an explicit emphasis on the reproducibility of the findings, including the availability of data and methodologies. Addressing the steps taken to ensure the robustness and reproducibility of the results, such as standardized protocols, sample size justifications, and statistical analysis methodologies, would bolster the study's credibility.

**RE:** We are grateful for the time and effort you have dedicated to reviewing our manuscript, and for your constructive feedback. We have carefully considered your comments and suggestions and have revised our manuscript accordingly. Below, we provide a point-by-point response to your comments.

In response to the comment on the reproducibility of findings and data availability, we fully acknowledge the importance of data openness for readers and researchers interested in our work. However, due to the ongoing nature of our series of research studies, and to protect the integrity of our ongoing analyses, we are not currently planning to make the original data publicly available. This is in line with the guidelines of many research institutions which encourage researchers to protect their work until it is fully complete. However, we are more than willing to provide the relevant data and code to researchers upon reasonable request. While we had previously stated in the "Availability of data and materials" section that "The datasets used and/or analyzed during the current study are available from the corresponding author on reasonable request.", we have now revised this statement to better highlight our commitment to data transparency and reproducibility: "All raw data supporting the conclusions of our study, as well as any relevant code, will be made available to any researcher for the purpose of reproducing the results or replicating the procedures, upon reasonable request to the corresponding author."

Regarding the suggestion to address the steps taken to ensure the robustness and reproducibility of our results, we have provided detailed descriptions of the standardized protocols and statistical analysis methodologies in the Methods section of our manuscript. These are available for interested researchers to replicate. As for the justification of sample sizes, we believe that this is not applicable as our study is a preliminary investigation using animal models. We have acknowledged this in the limitations section of our discussion, noting that our results are largely dependent on animal models. Nevertheless, we understand the importance of sample size justification in research and will ensure its inclusion in future studies where applicable.

We hope that these revisions and responses adequately address the reviewers' concerns and improve the quality and clarity of our manuscript. We sincerely thank you for your constructive feedback and look forward to your further comments.

Recommendations for Revision:

* Enhance Contextualization: Provide a more detailed comparison with existing literature to contextualize the findings within the broader field, including areas of agreement and contention.

**RE:** Thank you very much for your constructive comments and suggestions on our manuscript. We appreciate your time and effort spent on our paper, and your insightful critique which we believe will greatly improve the quality of our work.

We agree with the reviewer that the discussions would indeed benefit from a more detailed comparison with conflicting or complementary studies. However, as we have mentioned in our discussion, many of the key genes, gut microbiota and metabolites identified in our study are still not clearly linked to Acute Myocardial Infarction (AMI). The lack of a definitive relationship between these factors and AMI has constrained our ability to delve into a more detailed comparison and discussion.

We understand that our findings must be contextualized within the broader field, and we have tried our best to do so within the confines of the current knowledge. We have discussed the areas of agreement with existing literature, particularly the corroborative findings from Hongjun You et al., which enriches the contextual foundation of our study.

We hope that our revisions and clarifications meet your approval. We sincerely appreciate your constructive criticism, which has greatly improved our manuscript.

Thank you again for your time and consideration.

* Mechanistic Insights: Emphasize the need for mechanistic studies to understand the causal relationships underpinning the observed effects of CBLB on myocardial infarction, gut microbiota, and metabolic profiles.

**RE:** We greatly appreciate the time and effort you have invested in reviewing and evaluating our manuscript. We acknowledge the importance and necessity of the suggestions provided and have made a concerted effort to address each of them diligently.

In reference to the reviewer's comment on the need for further inquiry and a deeper exploration of underlying mechanisms, we concur with the reviewer's perspective. We understand that illuminating the mechanism pathways of how CBLB influences the prognosis of myocardial infarction is a complex task. Our current research does not allow us to pinpoint the precise mechanisms. However, to address this comment, we have supplemented our discussion section with a functional enrichment analysis of differentially expressed genes (DEGs) between the CBLC overexpression + AMI group and the AMI group. We hope that this addition will provide direction and inspiration for our future work and for other researchers interested in this study.

The reviewer's suggestion to emphasize the need for mechanistic studies to understand the causal relationships underpinning the observed effects of CBLB on myocardial infarction, gut microbiota, and metabolic profiles is indeed a significant consideration. However, our current findings do not definitively establish the relationships between these factors. To address this, we have supplemented our discussion with a section on literature-based developments to highlight the potential correlations.

We acknowledge the limitations of our study, as well as those in the broader field of research. In the description of these limitations, we candidly admit that "In terms of the specificity of the identified metabolites and genes, it should be noted that while they exhibited significant statistical correlation with AMI in our study, they may not be exclusive to AMI. Other diseases or physiological conditions may also influence their levels, which could introduce confounding factors. Furthermore, the role of these metabolites and genes in AMI is not completely understood. While we have presented correlative findings, causal relationships and mechanisms of action need to be confirmed through further studies."

We are grateful for your constructive comments and suggestions, which significantly contribute to the improvement of our manuscript. We have endeavored to address each of them adequately, and we believe that these revisions have greatly enhanced the quality of our manuscript.

* Clarify Novelty: More explicitly state how the findings advance the current understanding and address specific gaps in the literature.

**RE:** We are sincerely grateful for the constructive comments and valuable time invested in the review of our manuscript. Your feedback is greatly appreciated and has provided significant guidance for the improvement of our paper.

We are encouraged by the reviewer's recognition of the novelty of our findings, particularly the potential therapeutic implications of CBLB intervention in myocardial infarction and its systemic effects. Your positive feedback reaffirms the significance and value of our research.

The reviewer's recommendation to delineate how our findings advance the current understanding and identify specific gaps that the study addresses is well-taken. We agree that such elucidation would indeed enhance the discussion of the novelty of our study. Therefore, we have revised our discussion section to explicitly state how our findings contribute to advancing existing knowledge in the field. We have also identified and highlighted the specific gaps in the literature that our study addresses.

We appreciate the reviewer's constructive comments and suggestions, which have been instrumental in enhancing the quality of our manuscript. We believe that these revisions have significantly improved the clarity and quality of our manuscript.

Thank you once again for the valuable feedback. We look forward to hearing from you.

* Broaden Limitation Discussion: Expand the discussion on limitations to include considerations of experimental design, potential biases, and the implications for clinical translation.

**RE:** We would like to express our sincere appreciation for the insightful and valuable comments provided on our manuscript. We have carefully considered these suggestions and have made significant revisions to the manuscript accordingly.

We have taken the reviewer's recommendations to heart and have expanded the discussion on limitations to include considerations of experimental design, potential biases, and implications for clinical translation.

In response to the reviewer's comment on "Broaden Limitation Discussion", we have incorporated further detail on the limitations of our study. We now explicitly acknowledge that our selection of samples may have introduced bias into our research and that the bioinformatics tools used might also have inherent biases. We also acknowledge that despite our best efforts, some biases in data collection may still exist.

We also now discuss the limitations of our statistical models, including the assumption of certain characteristics of the data that may not always hold true in biological studies. We also acknowledge the risk of false positives due to the high-dimensional nature of the data and the reliance on existing databases in our in silico methods for data interpretation.

In addition, we have added a more nuanced discussion on the generalizability of our findings. We now point out that the identified metabolites and genes may not be exclusive to AMI and that their role in AMI is not completely understood.

We have also outlined our future research plans, including investigating the role of CBLB in different contexts of cardiovascular disease and in human patients, as well as potentially integrating emerging technologies or computational approaches in our research.

We hope this revised discussion provides a more balanced view and believe that these revisions have improved the manuscript. We appreciate the reviewer's constructive feedback, which was instrumental in enhancing the quality of our manuscript.

Thank you for considering our manuscript for publication in the Journal of Translational Medicine.

* Specific Future Directions: Outline detailed experimental or clinical studies that could validate and extend the findings, providing a clearer direction for future research.

**RE:** We sincerely thank the reviewer for their insightful comments and constructive suggestions. We appreciate the time and effort spent on reviewing our manuscript and the thought-provoking feedback that has allowed us to improve our work.

In response to the reviewer's comment on the need for a more detailed outline of specific experimental or clinical studies required to validate and extend our findings, we have incorporated this into our revised manuscript.

Firstly, as suggested, we have elaborated on our future research plans involving the investigation of CBLB's effects in diverse models of cardiovascular disease. We believe that this approach would not only serve to validate our current findings but also pave the way for a broader understanding of the role of CBLB in various cardiovascular diseases. We have reflected this in our discussion and agree that it indeed provides a clearer direction for future research.

Secondly, we have also emphasized our intention to explore the role of CBLB in human patients. We agree with the reviewer that this will provide a more realistic perspective of the implications of our research findings and strengthen the translatability of our study.

Lastly, responding to the implicit suggestion from the reviewer, we have acknowledged the potential of integrating emerging technologies and computational approaches in our research. We believe that including these methods in our future studies will enhance the robustness of our research and open up exciting new research avenues.

We trust that these revisions have adequately addressed the reviewer's concerns and have improved the quality of our manuscript. We look forward to hearing further feedback.

* Reproducibility Focus: Highlight the measures taken to ensure the reproducibility of the findings, including data availability and methodological transparency.

**RE:** We are grateful for the time and effort you have dedicated to reviewing our manuscript, and for your constructive feedback. We have carefully considered your comments and suggestions and have revised our manuscript accordingly. Below, we provide a point-by-point response to your comments.

In response to the comment on the reproducibility of findings and data availability, we fully acknowledge the importance of data openness for readers and researchers interested in our work. However, due to the ongoing nature of our series of research studies, and to protect the integrity of our ongoing analyses, we are not currently planning to make the original data publicly available. This is in line with the guidelines of many research institutions which encourage researchers to protect their work until it is fully complete. However, we are more than willing to provide the relevant data and code to researchers upon reasonable request. While we had previously stated in the "Availability of data and materials" section that "The datasets used and/or analyzed during the current study are available from the corresponding author on reasonable request.", we have now revised this statement to better highlight our commitment to data transparency and reproducibility: "All raw data supporting the conclusions of our study, as well as any relevant code, will be made available to any researcher for the purpose of reproducing the results or replicating the procedures, upon reasonable request to the corresponding author."

Regarding the suggestion to address the steps taken to ensure the robustness and reproducibility of our results, we have provided detailed descriptions of the standardized protocols and statistical analysis methodologies in the Methods section of our manuscript. These are available for interested researchers to replicate. As for the justification of sample sizes, we believe that this is not applicable as our study is a preliminary investigation using animal models. We have acknowledged this in the limitations section of our discussion, noting that our results are largely dependent on animal models. Nevertheless, we understand the importance of sample size justification in research and will ensure its inclusion in future studies where applicable.

We hope that these revisions and responses adequately address the reviewers' concerns and improve the quality and clarity of our manuscript. We sincerely thank you for your constructive feedback and look forward to your further comments.

This study represents a significant contribution to the field, offering novel insights into the potential therapeutic role of CBLB in myocardial infarction. Addressing the recommendations above would further enhance the impact and clarity of the discussion.

Review and Critical Analysis of the Conclusions Section

Critical Evaluation:

1. Summary of Findings: The conclusions succinctly summarize the study's primary achievements in uncovering the potential regulatory mechanisms of CBLB in acute myocardial infarction (AMI), alongside identifying key microorganisms, metabolites, and genes. This concise recapitulation underscores the novel contributions of the research to understanding AMI's etiology and the therapeutic promise of CBLB. However, the conclusions could be strengthened by explicitly summarizing the significant findings' implications for AMI research and treatment strategies, emphasizing how these insights advance the current state of knowledge.

**RE:** We appreciate your positive comments regarding our manuscript, particularly your recognition of our primary achievements in uncovering the potential regulatory mechanisms of CBLB in acute myocardial infarction (AMI), and the identification of key microorganisms, metabolites, and genes. Your acknowledgment of the novelty of our research and the potential therapeutic promise of CBLB in understanding AMI's etiology greatly motivates us.

We also appreciate your constructive suggestion to further strengthen our conclusions by explicitly summarizing the significant implications of our findings for AMI research and treatment strategies, and emphasizing how these insights advance the current state of knowledge.

In response to this, we have revised our manuscript's conclusion as follows: "In conclusion, this study unveils potential regulatory mechanisms of CBLB in AMI, identifying key microorganisms, metabolites, and genes, thereby enriching our understanding of AMI's etiology. These findings not only offer a fresh perspective on the underlying molecular processes of AMI but also lay a theoretical foundation for the potential therapeutic application of CBLB in AMI treatment."

We further elaborate on the implications of our findings in the revised manuscript as follows: "The significant implications of our findings for AMI research are multifold. From a basic research perspective, our study provides new avenues for understanding the pathophysiology of AMI at a molecular level. From a translational perspective, the identified molecules may serve as potential therapeutic targets, paving the way for the development of novel treatment strategies for AMI. Furthermore, these insights significantly advance the current state of knowledge by providing a more comprehensive picture of the molecular interplay in AMI, thereby bridging the gap between basic research and clinical application. Nevertheless, it is crucial to note that future studies are required for further validation and exploration of our findings, which will undoubtedly bring us closer to the goal of improving AMI management and patient outcomes."

We believe that this revision not only addresses your suggestion but also enhances our manuscript by providing a clearer and more comprehensive summary of our study's implications. We are grateful for your insightful review and constructive suggestions, which have undoubtedly improved the quality of our manuscript.

2. Theoretical and Practical Implications: The mention of laying a theoretical foundation for CBLB's potential use in treating AMI is valuable. It highlights the translational potential of the research findings. Nonetheless, the conclusions would benefit from a more detailed discussion on the practical implications of these findings, including potential pathways for clinical application and how these insights could inform future therapeutic developments or clinical practices.

**RE:** Thank you for taking the time to provide your insightful comments and suggestions on our manuscript. We appreciate your valuable feedback and have carefully addressed each of your concerns.

Regarding your comment on the presentation of practical implications in the Conclusions section, we are deeply appreciative of your suggestion. We understand that our research would significantly benefit from a more detailed discussion on the practical implications of our findings, including potential pathways for clinical application and how these insights could inform future therapeutic developments or clinical practices.

In response to your suggestion, we have added a comprehensive discussion in the manuscript to highlight the translational potential of our research. We have included a detailed comparison of our results with existing literature, which we believe would provide a broader perspective on the context and significance of our findings.

Moreover, we have elaborated on the future research directions based on our current findings. Specifically, we have highlighted our plans to investigate the role of CBLB in various contexts of cardiovascular disease and in human patients, which would not only validate our current findings but also provide new insights into the broader spectrum of cardiovascular diseases. We have also emphasized our interest in incorporating emerging technologies or computational approaches in our research, which we believe could provide exciting new perspectives on future research avenues.

In the Conclusions section, we have added the following text: "These findings not only offer a fresh perspective on the underlying molecular processes of AMI but also lay a theoretical foundation for the potential therapeutic application of CBLB in AMI treatment. The significant implications of our findings for AMI research are multifold. From a basic research perspective, our study provides new avenues for understanding the pathophysiology of AMI at a molecular level. From a translational perspective, the identified molecules may serve as potential therapeutic targets, paving the way for the development of novel treatment strategies for AMI. Furthermore, these insights significantly advance the current state of knowledge by providing a more comprehensive picture of the molecular interplay in AMI, thereby bridging the gap between basic research and clinical application. Nevertheless, it is crucial to note that future studies are required for further validation and exploration of our findings, which will undoubtedly bring us closer to the goal of improving AMI management and patient outcomes."

We believe that these additions significantly strengthen our manuscript by providing a more detailed and practical perspective on our research findings, while also addressing your concerns. We thank you again for your valuable suggestions and hope that you find these changes satisfactory.

3. Acknowledgment of Future Research Needs: The acknowledgment of the need for further studies to validate and explore the findings is crucial, reflecting scientific rigor and understanding of the research process's iterative nature. However, specifying areas where additional research is particularly needed or suggesting specific methodologies for future investigations could provide clearer guidance for subsequent work in the field.

**RE:** We appreciate your constructive comments and suggestions on our manuscript submitted to the Journal of Translational Medicine. We are grateful for the opportunity to improve our paper based on your insightful feedback, particularly in regards to the need for specifying future research directions and methodologies to validate and explore the current study's findings.

In response to your suggestion to "Offer specific suggestions for areas of focus in future research, including methodologies, to further validate and explore the study's findings", we concur with your view and have accordingly revised the Discussion and Conclusion sections of our manuscript. We have outlined a clear path for future investigations, detailing specific areas of research and potential methodologies that may be employed.

In the Discussion section, we have supplemented the following: "In addition to the systems biology strategies highlighted, we will also explore the effects of CBLB in different contexts of cardiovascular disease. This would not only validate our current findings but also open new avenues for understanding the role of CBLB in the broader spectrum of cardiovascular diseases. Furthermore, we also plan to investigate the role of CBLB in human patients to gain a real-world perspective of its implications. Lastly, we recognize the potential of integrating emerging technologies or computational approaches in our research, which we believe could provide exciting perspectives on future research avenues, and thus, we will consider incorporating these methods in our subsequent studies."

Moreover, in the Conclusion section, we have added: "From a translational perspective, the identified molecules may serve as potential therapeutic targets, paving the way for the development of novel treatment strategies for AMI. Nevertheless, it is crucial to note that future studies are required for further validation and exploration of our findings, which will undoubtedly bring us closer to the goal of improving AMI management and patient outcomes."

We believe these additions adequately address your concerns and provide a comprehensive roadmap for future research in this field. We hope that our revised manuscript now meets the standards of the Journal of Translational Medicine and look forward to your further comments and suggestions.

Thank you once again for your time and effort in reviewing our work.

4. Critical Self-Reflection: While the conclusions briefly touch upon future research requirements, a more critical reflection on the study's limitations and their impact on the conclusions drawn would enhance the section's depth. An acknowledgment of the study's scope, potential biases, and the generalizability of the findings would offer a more balanced and nuanced view of the research's contributions and limitations.

**RE:** We appreciate your valuable feedback on our manuscript submitted to the Journal of Translational Medicine. One of the major comments was to incorporate a critical assessment of the study's limitations and their implications for the conclusions drawn, which was said to provide a comprehensive understanding of the research's scope and impact.

We agree with your observations and acknowledge that a clear statement of the study's limitations and their implications is crucial for a comprehensive understanding of our research. However, we would like to clarify that we have indeed provided a thorough discussion of our study's limitations in the discussion section of the manuscript. We believe that it is essential to recognize any potential biases, the scope of our research, and the generalizability of our findings to ensure a balanced and nuanced view of our study's contributions and limitations.

To address your concern, we have provided a summary of the limitations of our study below:

1. Potential bias in sample selection: We employed a rigorous selection process to ensure that our samples were representative of the population in terms of age, sex, and other pertinent variables. However, we recognize that our selection of samples may have introduced some bias into our research.

2. Inherent biases in the bioinformatics tools and databases used: Despite our robust experimental design aimed at eliminating potential sources of bias, there might have been some inherent biases in the tools and databases we used.

3. Limitations in the statistical models: Our statistical models assume certain characteristics of the data that may not always hold true in biological studies. Despite multiple testing corrections, the risk of false positives remains due to the high-dimensional nature of the data.

4. Limitations in the in silico methods for data interpretation: While these methods rely on existing databases, they may miss novel interactions or pathways.

5. The mouse model used may not accurately represent the human physiological environment: Therefore, it is necessary to validate our findings in larger samples and diverse models.

6. The identified metabolites and genes are not exclusive to AMI: Other diseases or physiological conditions may also influence their levels, potentially leading to confounding factors. The role of these metabolites and genes in AMI is not completely understood and needs further studies.

We understand that these limitations could affect the conclusions made in our study and we have taken steps to mitigate their impact. We believe that acknowledging these limitations not only provides a comprehensive understanding of our research but also paves the way for future studies.

We hope this response addresses your concerns. Once again, we appreciate your insightful feedback, and we believe incorporating these points into our conclusions will strengthen our paper.

5. Recommendations for Clinical Translation: The conclusions mention the potential use of CBLB in treating AMI, indicating translational aspirations. A stronger conclusion would include recommendations for bridging the gap between these findings and clinical application. Discussing potential challenges in clinical translation, necessary steps for moving from animal models to human trials, and considerations for safety and efficacy assessments would provide valuable insights for readers interested in the translational impact of this research.

**RE:** We wholeheartedly appreciate your suggestion to bolster our conclusion by discussing potential challenges and necessary steps to facilitate the transition from our research findings to therapeutic application. We agree that such a discussion would provide valuable insights for readers interested in the translational impact of our research.

In response to this comment, we have revised the conclusion of our manuscript to elaborate on the potential challenges and necessary steps involved in translating our findings into clinical application. We now discuss the transition from animal models to human trials, the variability of patient responses due to genetic and environmental factors, and the need to evaluate the cost-effectiveness of therapies targeting CBLB.

We believe that these additional discussions significantly enhance our conclusion. They not only provide valuable insights into the potential challenges and necessary steps involved in clinical translation but also highlight the value of our research findings for the field of AMI treatment.

We hope that these revisions adequately address your concerns. We thank you once again for your constructive comments, which have greatly improved the quality of our manuscript.

Recommendations for Revision:

* Enhance Summary of Implications: Elaborate on the practical and theoretical implications of the findings, emphasizing their contribution to the existing body of knowledge and potential impact on AMI treatment strategies.

**RE:** We appreciate your positive comments regarding our manuscript, particularly your recognition of our primary achievements in uncovering the potential regulatory mechanisms of CBLB in acute myocardial infarction (AMI), and the identification of key microorganisms, metabolites, and genes. Your acknowledgment of the novelty of our research and the potential therapeutic promise of CBLB in understanding AMI's etiology greatly motivates us.

We also appreciate your constructive suggestion to further strengthen our conclusions by explicitly summarizing the significant implications of our findings for AMI research and treatment strategies, and emphasizing how these insights advance the current state of knowledge.

In response to this, we have revised our manuscript's conclusion as follows: "In conclusion, this study unveils potential regulatory mechanisms of CBLB in AMI, identifying key microorganisms, metabolites, and genes, thereby enriching our understanding of AMI's etiology. These findings not only offer a fresh perspective on the underlying molecular processes of AMI but also lay a theoretical foundation for the potential therapeutic application of CBLB in AMI treatment."

We further elaborate on the implications of our findings in the revised manuscript as follows: "The significant implications of our findings for AMI research are multifold. From a basic research perspective, our study provides new avenues for understanding the pathophysiology of AMI at a molecular level. From a translational perspective, the identified molecules may serve as potential therapeutic targets, paving the way for the development of novel treatment strategies for AMI. Furthermore, these insights significantly advance the current state of knowledge by providing a more comprehensive picture of the molecular interplay in AMI, thereby bridging the gap between basic research and clinical application. Nevertheless, it is crucial to note that future studies are required for further validation and exploration of our findings, which will undoubtedly bring us closer to the goal of improving AMI management and patient outcomes."

We believe that this revision not only addresses your suggestion but also enhances our manuscript by providing a clearer and more comprehensive summary of our study's implications. We are grateful for your insightful review and constructive suggestions, which have undoubtedly improved the quality of our manuscript.

* Specify Future Research Directions: Offer specific suggestions for areas of focus in future research, including methodologies, to further validate and explore the study's findings.

**RE:** We appreciate your constructive comments and suggestions on our manuscript submitted to the Journal of Translational Medicine. We are grateful for the opportunity to improve our paper based on your insightful feedback, particularly in regards to the need for specifying future research directions and methodologies to validate and explore the current study's findings.

In response to your suggestion to "Offer specific suggestions for areas of focus in future research, including methodologies, to further validate and explore the study's findings", we concur with your view and have accordingly revised the Discussion and Conclusion sections of our manuscript. We have outlined a clear path for future investigations, detailing specific areas of research and potential methodologies that may be employed.

In the Discussion section, we have supplemented the following: "In addition to the systems biology strategies highlighted, we will also explore the effects of CBLB in different contexts of cardiovascular disease. This would not only validate our current findings but also open new avenues for understanding the role of CBLB in the broader spectrum of cardiovascular diseases. Furthermore, we also plan to investigate the role of CBLB in human patients to gain a real-world perspective of its implications. Lastly, we recognize the potential of integrating emerging technologies or computational approaches in our research, which we believe could provide exciting perspectives on future research avenues, and thus, we will consider incorporating these methods in our subsequent studies."

Moreover, in the Conclusion section, we have added: "From a translational perspective, the identified molecules may serve as potential therapeutic targets, paving the way for the development of novel treatment strategies for AMI. Nevertheless, it is crucial to note that future studies are required for further validation and exploration of our findings, which will undoubtedly bring us closer to the goal of improving AMI management and patient outcomes."

We believe these additions adequately address your concerns and provide a comprehensive roadmap for future research in this field. We hope that our revised manuscript now meets the standards of the Journal of Translational Medicine and look forward to your further comments and suggestions.

Thank you once again for your time and effort in reviewing our work.

* Incorporate Critical Reflection: Integrate a critical assessment of the study's limitations and their implications for the conclusions drawn, providing a more comprehensive understanding of the research's scope and impact.

**RE:** We appreciate your valuable feedback on our manuscript submitted to the Journal of Translational Medicine. One of the major comments was to incorporate a critical assessment of the study's limitations and their implications for the conclusions drawn, which was said to provide a comprehensive understanding of the research's scope and impact.

We agree with your observations and acknowledge that a clear statement of the study's limitations and their implications is crucial for a comprehensive understanding of our research. However, we would like to clarify that we have indeed provided a thorough discussion of our study's limitations in the discussion section of the manuscript. We believe that it is essential to recognize any potential biases, the scope of our research, and the generalizability of our findings to ensure a balanced and nuanced view of our study's contributions and limitations.

To address your concern, we have provided a summary of the limitations of our study below:

1. Potential bias in sample selection: We employed a rigorous selection process to ensure that our samples were representative of the population in terms of age, sex, and other pertinent variables. However, we recognize that our selection of samples may have introduced some bias into our research.

2. Inherent biases in the bioinformatics tools and databases used: Despite our robust experimental design aimed at eliminating potential sources of bias, there might have been some inherent biases in the tools and databases we used.

3. Limitations in the statistical models: Our statistical models assume certain characteristics of the data that may not always hold true in biological studies. Despite multiple testing corrections, the risk of false positives remains due to the high-dimensional nature of the data.

4. Limitations in the in silico methods for data interpretation: While these methods rely on existing databases, they may miss novel interactions or pathways.

5. The mouse model used may not accurately represent the human physiological environment: Therefore, it is necessary to validate our findings in larger samples and diverse models.

6. The identified metabolites and genes are not exclusive to AMI: Other diseases or physiological conditions may also influence their levels, potentially leading to confounding factors. The role of these metabolites and genes in AMI is not completely understood and needs further studies.

We understand that these limitations could affect the conclusions made in our study and we have taken steps to mitigate their impact. We believe that acknowledging these limitations not only provides a comprehensive understanding of our research but also paves the way for future studies.

We hope this response addresses your concerns. Once again, we appreciate your insightful feedback, and we believe incorporating these points into our conclusions will strengthen our paper.

* Detail Clinical Translation Pathways: Include considerations and recommendations for the clinical translation of the findings, discussing potential challenges and necessary steps to facilitate the transition from research to therapeutic application.

**RE:** We wholeheartedly appreciate your suggestion to bolster our conclusion by discussing potential challenges and necessary steps to facilitate the transition from our research findings to therapeutic application. We agree that such a discussion would provide valuable insights for readers interested in the translational impact of our research.

In response to this comment, we have revised the conclusion of our manuscript to elaborate on the potential challenges and necessary steps involved in translating our findings into clinical application. We now discuss the transition from animal models to human trials, the variability of patient responses due to genetic and environmental factors, and the need to evaluate the cost-effectiveness of therapies targeting CBLB.

We believe that these additional discussions significantly enhance our conclusion. They not only provide valuable insights into the potential challenges and necessary steps involved in clinical translation but also highlight the value of our research findings for the field of AMI treatment.

We hope that these revisions adequately address your concerns. We thank you once again for your constructive comments, which have greatly improved the quality of our manuscript.

The conclusion effectively encapsulates the study's contributions and acknowledges the need for further research. Enhancing the section with the above recommendations will strengthen the clarity, impact, and comprehensiveness of the study's closing remarks, offering valuable insights for future research and clinical application pathways.

Review and Critical Analysis of the References Section

The References section is a critical component of any scientific manuscript, providing the foundation of prior work upon which the current study builds. A thorough and critical analysis of the references listed in this manuscript yields several observations and recommendations:

1. Relevance and Currency:

The references span a range of years, demonstrating a thorough review of both foundational and cutting-edge research in the fields of acute myocardial infarction (AMI), CBLB function, microbiota, and metabolomics. This breadth ensures that readers understand the historical context and the latest developments. However, the inclusion of very recent studies (e.g., 2023 and 2024 references for AMI biomarkers and metabolic biomarkers) is commendable, reflecting the manuscript's currency and relevance.

**RE:** We are grateful for the insightful comments and suggestions provided to improve our manuscript. Particularly, we appreciate the recommendation to continuously update our literature review to include the most recent and relevant studies, thereby enhancing the manuscript's relevance and currency.

In response to this suggestion, we have made amendments to our references to ensure they represent both the historical context and current developments in our field of study, as highlighted in your comments. Notably, we have included two additional studies: "Multinomial machine learning identifies independent biomarkers by integrated metabolic analysis of acute coronary syndrome" (PMID: 37996510), and "Gut microbiota combined with metabolites reveals unique features of acute myocardial infarction patients different from stable coronary artery disease" (PMID: 35750287). These studies further substantiate the assertion in our introduction that recent research has revealed specific changes in the gut microbiota and in vivo metabolites in AMI patients.

We are confident that these additions will not only demonstrate the breadth of our literature review but also reflect the manuscript's currency and relevance to the current research landscape.

Thank you again for your constructive feedback. We believe that these revisions will significantly improve our manuscript, and we are looking forward to your continued guidance.

2. Diversity of Sources:

The list encompasses a wide array of sources, including high-impact journals, which speaks to the rigorous research and depth of investigation conducted by the authors. It covers various aspects pertinent to the study, such as AMI management, the role of CBLB in immunity, microbiota analysis, and metabolomics. Ensuring that sources from a wide range of disciplines are included is crucial for interdisciplinary studies like this one.

**RE:** We appreciate your insightful comments and suggestions on our manuscript. We are particularly grateful for your positive remarks on the diversity of sources we used in our references, which testifies to the rigorous research and depth of investigation we conducted.

We understand the importance of maintaining a diverse range of sources, especially for interdisciplinary studies like ours. As such, we have ensured that our references cover various aspects pertinent to our study, including AMI management, the role of CBLB in immunity, microbiota analysis, and metabolomics, and originate from a wide variety of disciplines.

Your recognition of the breadth of our sources is greatly encouraging. We have strived to include high-impact journals to provide our readers with a comprehensive understanding of the current state of research in these areas.

Thank you once again for your constructive feedback. We are dedicated to improving our manuscript further and look forward to any additional suggestions you may have.

3. Specificity and Direct Relevance:

References directly related to the study's core themes, such as the role of CBLB in immune regulation and its potential as a biomarker for AMI, provide a solid background and justify the research focus. The inclusion of studies on the gut microbiome and metabolomics in the context of cardiovascular diseases adds depth to the rationale for the chosen methodologies and analytical approaches. Ensuring that each reference directly supports the manuscript's assertions is vital for a coherent narrative.

**RE:** Thank you for providing us with the insightful comments and suggestions. We deeply appreciate your guidance on specifically emphasizing the direct relevance of our references to our study's core themes.

In response to your suggestion, we have carefully revised our manuscript and added two critical references: "Multinomial machine learning identifies independent biomarkers by integrated metabolic analysis of acute coronary syndrome" (PMID: 37996510) and "Gut microbiota combined with metabolites reveals unique features of acute myocardial infarction patients different from stable coronary artery disease" (PMID: 35750287). These additional references provide a more comprehensive understanding of the role of gut microbiota and in vivo metabolites in acute myocardial infarction (AMI), which directly support the assertions made in our manuscript.

We believe these additions not only strengthen our argument but also ensure the specificity and direct relevance of our references to the study's core themes, as per your excellent advice. We have strived to ensure that each reference directly supports the manuscript's assertions, thereby contributing to a coherent narrative.

We sincerely appreciate your constructive feedback, which has greatly helped improve our manuscript. We look forward to any further suggestions you may have.

4. Technical and Methodological References:

The manuscript does well to include references for specific techniques and software used in the study (e.g., HISAT2 for genome alignment, DESeq2 for differential expression analysis). This not only aids in the transparency and reproducibility of the research but also guides readers interested in the technical details of the methodologies employed. It might be beneficial to also reference methodological reviews or guidelines that support the chosen approaches or analytical thresholds, providing further validation for the study's design.

**RE:** Thank you for your insightful comments and suggestions on our manuscript. We appreciate your time and effort in reviewing our work and we believe that your feedback will greatly enhance the quality of our paper. Herein, we provide our point-by-point response to each of the reviewer's comments.

Regarding the comment on the inclusion of references for specific techniques and software used in the study, we appreciate your positive feedback. We would like to clarify that in our initial submission, we have cited the appropriate references for both "HISAT2 for genome alignment" and "DESeq2 for differential expression analysis". The former is referenced by "Graph-based genome alignment and genotyping with HISAT2 and HISAT-genotype" (PMID: 31375807) and the latter by "Moderated estimation of fold change and dispersion for RNA-seq data with DESeq2" (PMID: 25516281).

With respect to the suggestion of referencing methodological reviews or guidelines that support the chosen approaches or analytical thresholds, we understand the value of this proposition. However, it's important to note that the methods chosen for a particular study are not necessarily uniform across all studies, as they not only depend on the nature of the research but also on the expertise and preferences of the researchers. Similarly, the thresholds set for analysis are typically within a range that is common knowledge among researchers and may not have specific references. Nonetheless, we agree that wherever possible, we will make an effort to cite relevant methodological reviews or guidelines.

Concerning the recommendation for including references or DOIs for the datasets used in our study, we acknowledge the importance of this suggestion for enhancing transparency and allowing readers to further explore the data. We assure that we have provided accessible URLs or ensured that the corresponding references can be traced in PubMed at appropriate locations in the revised manuscript.

In summary, we are grateful for your constructive comments and have made every effort to address them to the best of our ability. We believe these revisions have strengthened our manuscript and look forward to your further review.

Recommendations for Improvement:

* Comprehensiveness: Ensure that all pivotal studies relevant to the manuscript's core themes are included. For interdisciplinary work, this might mean broadening the search to include key studies from adjacent fields that might not have been directly cited but influence the study's context and implications.

**RE:** We appreciate your insightful comments and suggestions on our manuscript. We are particularly grateful for your positive remarks on the diversity of sources we used in our references, which testifies to the rigorous research and depth of investigation we conducted.

We understand the importance of maintaining a diverse range of sources, especially for interdisciplinary studies like ours. As such, we have ensured that our references cover various aspects pertinent to our study, including AMI management, the role of CBLB in immunity, microbiota analysis, and metabolomics, and originate from a wide variety of disciplines.

Your recognition of the breadth of our sources is greatly encouraging. We have strived to include high-impact journals to provide our readers with a comprehensive understanding of the current state of research in these areas.

Thank you once again for your constructive feedback. We are dedicated to improving our manuscript further and look forward to any additional suggestions you may have.

* Up-to-Date References: Continuously update the literature review up to the manuscript submission to include the most recent and relevant studies, enhancing the manuscript's relevance and currency.

**RE:** We are grateful for the insightful comments and suggestions provided to improve our manuscript. Particularly, we appreciate the recommendation to continuously update our literature review to include the most recent and relevant studies, thereby enhancing the manuscript's relevance and currency.

In response to this suggestion, we have made amendments to our references to ensure they represent both the historical context and current developments in our field of study, as highlighted in your comments. Notably, we have included two additional studies: "Multinomial machine learning identifies independent biomarkers by integrated metabolic analysis of acute coronary syndrome" (PMID: 37996510), and "Gut microbiota combined with metabolites reveals unique features of acute myocardial infarction patients different from stable coronary artery disease" (PMID: 35750287). These studies further substantiate the assertion in our introduction that recent research has revealed specific changes in the gut microbiota and in vivo metabolites in AMI patients.

We are confident that these additions will not only demonstrate the breadth of our literature review but also reflect the manuscript's currency and relevance to the current research landscape.

Thank you again for your constructive feedback. We believe that these revisions will significantly improve our manuscript, and we are looking forward to your continued guidance.

* Balance Between Primary and Review Articles: While primary research articles provide the evidence base for the study's claims, review articles can offer comprehensive overviews and insights into the current state of research, potentially enriching the background and discussion sections.

**RE:** We sincerely appreciate the insightful comments and suggestions provided to improve the quality of our manuscript. We acknowledge the importance and necessity of balancing the reference types between primary research and review articles to provide a comprehensive understanding of the subject to the readers.

In our initial submission, we have included a mix of both types of references. Several review articles such as "Modern management of acute myocardial infarction" (PMID: 17097446), "Mechanical Complications of Acute Myocardial Infarction: A Scientific Statement From the American Heart Association" (PMID: 34126755) and "Immune regulation of cardiac fibrosis post myocardial infarction" (PMID: 33207261) have been utilized to provide a broad overview of the current state of research in the field. Concurrently, we have cited primary research articles such as "Intestinal microbial metabolism of phosphatidylcholine and cardiovascular risk" (PMID: 23614584) and "Cecal Gut Microbiota and Metabolites Might Contribute to the Severity of Acute Myocardial Ischemia by Impacting the Intestinal Permeability, Oxidative Stress, and Energy Metabolism" (PMID: 31428065) to substantiate our claims with robust empirical evidence.

However, we are open to revisiting the references and making necessary adjustments to further enhance the balance between primary and review articles if the reviewer feels it is required. We believe this will indeed enrich the background and discussion sections of our paper, providing a more holistic picture of our research.

Thank you again for your constructive feedback. We look forward to your further suggestions to improve our work.

* Consistency in Reference Formatting: Ensure that all references are formatted consistently according to the journal's guidelines, including the correct use of italics for journal names, proper capitalization, and punctuation.

**RE:** We are grateful for your insightful review and advice regarding the formatting of our references. We acknowledge the importance of adhering to the journal's guidelines to ensure consistency and professionalism in our manuscript.

In response to your suggestion, we have meticulously revised our paper and made sure that all references are formatted consistently according to the guidelines set by the Journal of Translational Medicine. We utilized a citation management tool to systematically format all references. This includes the correct use of italics for journal names, proper capitalization, and punctuation.

We understand the significance of maintaining uniformity in reference formatting as it not only enhances the readability, but also upholds the credibility of our research paper. We believe that these revisions will help ensure clarity and consistency throughout the manuscript.

Thank you again for your constructive feedback. We are committed to meeting the high standards of the Journal of Translational Medicine and appreciate your guidance in helping us achieve this.

* Accessibility of Referenced Data: For studies that rely on public datasets or have generated significant amounts of data, including references or DOIs for the datasets can enhance transparency and enable readers to explore the data further.

**RE:** Thank you for your insightful comments and suggestions on our manuscript. We appreciate your time and effort in reviewing our work and we believe that your feedback will greatly enhance the quality of our paper. Herein, we provide our point-by-point response to each of the reviewer's comments.

Regarding the comment on the inclusion of references for specific techniques and software used in the study, we appreciate your positive feedback. We would like to clarify that in our initial submission, we have cited the appropriate references for both "HISAT2 for genome alignment" and "DESeq2 for differential expression analysis". The former is referenced by "Graph-based genome alignment and genotyping with HISAT2 and HISAT-genotype" (PMID: 31375807) and the latter by "Moderated estimation of fold change and dispersion for RNA-seq data with DESeq2" (PMID: 25516281).

With respect to the suggestion of referencing methodological reviews or guidelines that support the chosen approaches or analytical thresholds, we understand the value of this proposition. However, it's important to note that the methods chosen for a particular study are not necessarily uniform across all studies, as they not only depend on the nature of the research but also on the expertise and preferences of the researchers. Similarly, the thresholds set for analysis are typically within a range that is common knowledge among researchers and may not have specific references. Nonetheless, we agree that wherever possible, we will make an effort to cite relevant methodological reviews or guidelines.

Concerning the recommendation for including references or DOIs for the datasets used in our study, we acknowledge the importance of this suggestion for enhancing transparency and allowing readers to further explore the data. We assure that we have provided accessible URLs or ensured that the corresponding references can be traced in PubMed at appropriate locations in the revised manuscript.

In summary, we are grateful for your constructive comments and have made every effort to address them to the best of our ability. We believe these revisions have strengthened our manuscript and look forward to your further review.

In summary, the References section of this manuscript demonstrates a well-researched foundation for the study. By addressing the recommendations provided, the authors can further enhance the section's utility and contribution to the manuscript's overall strength and coherence.

Review and Critical Analysis of Figure Legends, Supplementary Figures, and Supplementary Tables

Figure Legends:

* Clarity and Detail: The figure legends are detailed, providing a clear description of what is depicted in each figure, including experimental groups, staining methods, and the statistical significance represented. The legends help readers understand the context and outcomes of the experiments without referring back to the main text. However, for figures showing comparative analyses (e.g., Fig. 1, Fig. 2), including brief statements about the biological significance of these comparisons could enhance understanding.

**RE:** We are grateful for your insightful comments and suggestions. Your feedback is greatly appreciated as it helps us improve the quality and readability of our manuscript.

We acknowledge the importance of providing clear and detailed figure legends, including the biological significance of the findings depicted in the figures. This not only enhances understanding for the readers but also strengthens the narrative flow of our paper.

In response to your suggestion, we have revised the legends of the comparative analysis figures (e.g., Fig. 1, Fig. 2) to include brief explanations of the biological relevance or implications of our findings. These additions aim to provide readers with more context and to clarify the significance of our results without them having to refer back to the main text.

For clarity, we have incorporated a study flow chart into our research. Consequently, Figures 1 and 2 in the original manuscript are now referred to as Figures 2 and 3 in the revised version.

We believe that these revisions will markedly improve the comprehension of our figures and the overall narrative of our research. We are committed to maintaining high standards of clarity and detail in our paper.

Thank you again for your constructive feedback. We look forward to your further suggestions to improve our work.

* Visual Data Representation: The description suggests that the figures effectively visualize complex data (e.g., microbiota composition, metabolites, DEGs) in an accessible manner. The use of scale bars, color coding, and differentiation between statistical significances is commendable. Nonetheless, ensuring that these visual elements are consistent across figures for easier comparison would be beneficial.

**RE:** We are thankful for your meticulous review and valuable feedback on our manuscript, particularly with respect to maintaining consistency in the visual elements across all figures.

In response to your comment regarding the consistency of color codes, we have revisited all the figures, including supplementary figures. We have now rectified and unified the color blocks representing Groups A, B, and C in the heat maps pertaining to gene expression in Figure 5 and Figure 6 of the original manuscript.

In the process of reviewing, we realized an oversight in the labeling of groups in Figure 6F of the original manuscript, where we inadvertently marked Groups BC as Groups BA in the heat map of differentially expressed genes (DEGs) between the two groups. We acknowledge this oversight and have now corrected it. We have also synchronized this correction in the legend of Figure 6 of the original manuscript, where a similar error was found.

In response to your recommendation, the figure originally numbered as Figure 6 in the manuscript has been renumbered to Figure 7 in the revised version, to accommodate the addition of a comprehensive study flowchart.

We have also reviewed and confirmed that the scale bars and symbols indicating statistical significance are consistent across all figures, as per your recommendation.

We believe these revisions will improve the consistency in data representation across our manuscript and facilitate a better understanding for the readers. We appreciate your constructive comments and look forward to your continuous guidance to improve our work.

Supplementary Figures:

* Supplementary Fig. 1-8: These figures seem to provide essential support to the main text, offering visual evidence of experimental procedures, results, and analyses. Including these as supplementary material is appropriate, as it does not overload the main text with data but remains accessible for interested readers. A suggestion would be to ensure that each supplementary figure is directly referenced within the main text at relevant points to guide readers towards these additional resources.

**RE:** We appreciate your constructive feedback on our manuscript submitted to the Journal of Translational Medicine. We are particularly grateful for your positive appraisal of our supplementary figures, acknowledging their role in supporting the main text without overwhelming it with data.

Your suggestion regarding the clear referencing of each supplementary figure in the main text is well received. We recognize the importance of guiding readers to additional resources that provide insights into the experimental procedures, results, and analyses which substantiate the conclusions of our study.

In response to your suggestion, we have meticulously reviewed our manuscript and ensured that each supplementary figure is explicitly referenced at the relevant points in the main text. This will provide a better navigation to our readers and effectively guide them towards these additional resources.

We believe that this modification further strengthens the coherence of our manuscript and enhances the reading experience. We are grateful for your thoughtful comments and await further guidance to improve our work.

Supplementary Tables:

* Comprehensiveness: The supplementary tables appear to offer a wealth of detailed data supporting the study's findings, from plasmid configurations to the identification of key genes and metabolites. This level of detail is crucial for reproducibility and further analysis by the scientific community. It would be beneficial if the methods section includes brief descriptions of how these tables were derived, ensuring readers can follow the logic and methodology behind these compilations.

**RE:** We greatly appreciate your constructive comments and the time you have taken to review our manuscript. Your feedback regarding the comprehensiveness of our supplementary tables and their contribution to the reproducibility of our study is highly valued.

In response to your suggestion to include brief descriptions in the methods section of how the data in the supplementary tables were derived, we would like to clarify that the data presented in these tables were generated during our analysis process. The methodologies and software used for this purpose have already been described in the methods section of our paper.

We understand your perspective on enhancing transparency and reproducibility. We believe that readers can follow the logic and methodology behind the compilation of these tables by referring to the descriptions of the analysis process outlined in our methods section. We hope that this approach balances the need for methodological detail with the practical limitations of length and complexity in the manuscript.

However, in an effort to address your concern, we have revisited our methods section and made sure that each reference to the analysis process clearly links to the corresponding supplementary table. This will further guide the readers in understanding how the data in the supplementary tables were derived.

Once again, we greatly appreciate your insightful comments and look forward to your continued guidance.

General Recommendations:

1. Enhance Context in Figure Legends: Include brief explanations of the biological relevance or implications of the findings depicted in figures to provide readers with context and enhance the narrative flow.

**RE:** We are grateful for your insightful comments and suggestions. Your feedback is greatly appreciated as it helps us improve the quality and readability of our manuscript.

We acknowledge the importance of providing clear and detailed figure legends, including the biological significance of the findings depicted in the figures. This not only enhances understanding for the readers but also strengthens the narrative flow of our paper.

In response to your suggestion, we have revised the legends of the comparative analysis figures (e.g., Fig. 1, Fig. 2) to include brief explanations of the biological relevance or implications of our findings. These additions aim to provide readers with more context and to clarify the significance of our results without them having to refer back to the main text.

For clarity, we have incorporated a study flow chart into our research. Consequently, Figures 1 and 2 in the original manuscript are now referred to as Figures 2 and 3 in the revised version.

We believe that these revisions will markedly improve the comprehension of our figures and the overall narrative of our research. We are committed to maintaining high standards of clarity and detail in our paper.

Thank you again for your constructive feedback. We look forward to your further suggestions to improve our work.

2. Consistency in Visual Elements: Ensure consistency in the use of visual elements across all figures to facilitate comparison and understanding. This includes scale bars, color codes, and symbols for statistical significance.

**RE:** We are thankful for your meticulous review and valuable feedback on our manuscript, particularly with respect to maintaining consistency in the visual elements across all figures.

In response to your comment regarding the consistency of color codes, we have revisited all the figures, including supplementary figures. We have now rectified and unified the color blocks representing Groups A, B, and C in the heat maps pertaining to gene expression in Figure 5 and Figure 6 of the original manuscript.

In the process of reviewing, we realized an oversight in the labeling of groups in Figure 6F, where we inadvertently marked Groups BC as Groups BA in the heat map of differentially expressed genes (DEGs) between the two groups. We acknowledge this oversight and have now corrected it. We have also synchronized this correction in the legend of Figure 6, where a similar error was found.

We have also reviewed and confirmed that the scale bars and symbols indicating statistical significance are consistent across all figures, as per your recommendation.

In response to your recommendation, the figure originally numbered as Figure 6 in the manuscript has been renumbered to Figure 7 in the revised version, to accommodate the addition of a comprehensive study flowchart.

We believe these revisions will improve the consistency in data representation across our manuscript and facilitate a better understanding for the readers. We appreciate your constructive comments and look forward to your continuous guidance to improve our work.

3. Reference Supplementary Figures in Main Text: Clearly reference each supplementary figure within the main text to direct readers to additional data that supports the study's conclusions.

**RE:** We appreciate your constructive feedback on our manuscript submitted to the Journal of Translational Medicine. We are particularly grateful for your positive appraisal of our supplementary figures, acknowledging their role in supporting the main text without overwhelming it with data.

Your suggestion regarding the clear referencing of each supplementary figure in the main text is well received. We recognize the importance of guiding readers to additional resources that provide insights into the experimental procedures, results, and analyses which substantiate the conclusions of our study.

In response to your suggestion, we have meticulously reviewed our manuscript and ensured that each supplementary figure is explicitly referenced at the relevant points in the main text. This will provide a better navigation to our readers and effectively guide them towards these additional resources.

We believe that this modification further strengthens the coherence of our manuscript and enhances the reading experience. We are grateful for your thoughtful comments and await further guidance to improve our work.

4. Methodological Descriptions for Supplementary Tables: Include in the methods section brief descriptions of the procedures leading to the data presented in supplementary tables, enhancing transparency and reproducibility.

**RE:** We greatly appreciate your constructive comments and the time you have taken to review our manuscript. Your feedback regarding the comprehensiveness of our supplementary tables and their contribution to the reproducibility of our study is highly valued.

In response to your suggestion to include brief descriptions in the methods section of how the data in the supplementary tables were derived, we would like to clarify that the data presented in these tables were generated during our analysis process. The methodologies and software used for this purpose have already been described in the methods section of our paper.

We understand your perspective on enhancing transparency and reproducibility. We believe that readers can follow the logic and methodology behind the compilation of these tables by referring to the descriptions of the analysis process outlined in our methods section. We hope that this approach balances the need for methodological detail with the practical limitations of length and complexity in the manuscript.

However, in an effort to address your concern, we have revisited our methods section and made sure that each reference to the analysis process clearly links to the corresponding supplementary table. This will further guide the readers in understanding how the data in the supplementary tables were derived.

Once again, we greatly appreciate your insightful comments and look forward to your continued guidance.

5. Accessibility and Navigation: Consider the accessibility of supplementary materials and ease of navigation for readers, ensuring that supplementary figures and tables are easily accessible and well-integrated with the main text.

**RE:** We tremendously appreciate your insightful comments on our manuscript, particularly regarding improving the accessibility and navigation of our supplementary materials. We understand the importance of making our supplementary figures and tables easily accessible and well-integrated with the main text for our readers.

In response to your suggestion, we have taken measures to ensure the accessibility of our supplementary materials. We have clearly referenced each supplementary figure and table in the main text at the relevant points, guiding readers to these additional resources. This will provide a better navigation to our readers and effectively direct them towards the supporting evidence for our study's conclusions.

Furthermore, we have ensured that the supplementary figures and tables are formatted in a user-friendly manner and are consistent with the overall style of the manuscript. We have also made sure that their legends are clearly written and provide sufficient context and explanation to the readers.

We believe that these steps will significantly improve the readability and accessibility of our supplementary materials, providing a seamless reading experience to our readers. We are grateful for your thoughtful comments and await further guidance to improve our work.

By addressing these points, the authors can significantly enhance the clarity, impact, and accessibility of the figure legends, supplementary figures, and tables, thereby providing a comprehensive and transparent account of their research findings and methodologies.

**Reviewer #2**: The authors evaluated the intervention role of CBLB in acute myocardial infarction by evaluating the disease pathophysiology and by characterizing the levels of cardiac genes, serum metabolites, and gut microbiota using the global methods of 16S rDNA sequencing, non-targeted metabolite sequencing, and RNA-seq.

**RE:** We are grateful for your overall summarization of our study and the time you took to review our manuscript. We appreciate your acknowledgment of our evaluation of the intervention role of CBLB in acute myocardial infarction by characterizing the levels of cardiac genes, serum metabolites, and gut microbiota using global methods of 16S rDNA sequencing, non-targeted metabolite sequencing, and RNA-seq.

In response to your summary, we would like to affirm that your understanding of our work is accurate. We have indeed conducted a comprehensive investigation into the effects of CBLB on acute myocardial infarction, focusing on the pathophysiology of the disease and the characterization of cardiac genes, serum metabolites, and gut microbiota. This has been achieved through state-of-the-art techniques like 16S rDNA sequencing, non-targeted metabolite sequencing, and RNA-seq, which have enabled us to generate compelling evidence in support of our findings.

We believe that our study provides valuable insights into the pathogenesis of acute myocardial infarction and the potential therapeutic role of CBLB. It is our hope that this research will pave the way for further investigations in this field and contribute to the development of novel interventions for this debilitating condition.

Once again, we greatly appreciate your insightful comments and look forward to your continued guidance.

1) The study is designed to characterize the impact of CBLB on pathophysiology and molecular and metabolite changes in AMI animal models. To perform the study, authors overexpressed CBLB using lentiviral transduction. The immunohistochemical analysis showed that CBLB expression remains unchanged between groups A and C and barely significant between B and C, questioning the over-expression of CBLB. Also, the endogenous expression of CBLB did not alter in AMI's group compared to the sham group, unlike reported in previous studies by H You and M Dong. Since the findings of the whole manuscript relied on over-expression of CBLB, authors are advised to validate CBLB overexpression using other methods such as western blot and qRT-PCR.

**RE:** We sincerely appreciate the time and effort the reviewer has dedicated in providing insightful and constructive comments on our manuscript. We have given careful consideration to each point raised and have made appropriate revisions, as detailed below. We believe these revisions have significantly improved the quality and clarity of our manuscript.

In response to the reviewer's comment on "CBLB expression barely significant between B and C, questioning the over-expression of CBLB," we have reevaluated our results, specifically Figure 2A. Our statistical analysis indicates that CBLB expression is relatively higher in group C compared to group B, with a P-value of 0.0499, satisfying our set criteria for statistical significance (P<0.05). We concede that this difference might appear as "barely significant," yet when considered in conjunction with the HE and MASSON staining results, the cardiac condition of mice in group C indeed shows better progress than those in group B. We agree with the reviewer's suggestion to validate CBLB overexpression using other methods such as western blot and qRT-PCR. Regrettably, due to financial constraints and the scarcity of biological samples, we are currently unable to supplement our study with these validation tests. However, we acknowledge this limitation in our initial experimental design and are committed to incorporating such validation in our future investigations.

Regarding the comment "the endogenous expression of CBLB did not alter in AMI's group compared to the sham group, unlike reported in previous studies by H You and M Dong," we do not perceive any contradiction with our study. We have meticulously reviewed the study by H You and M Dong (Identification of Immuno-Inflammation-Related Biomarkers for Acute Myocardial Infarction Based on Bioinformatics; PMID:37576155). Their research indicates a significant downregulation of CBLB in AMI patients compared to the control group, evident from both public database analysis and analysis of 20 peripheral blood samples. This is inconsistent with the statement that "the endogenous expression of CBLB did not alter in AMI's group compared to the sham group." Furthermore, drawing from their findings, we hypothesized that there is a progressive downregulation of the CBLB gene during the transition from a healthy state to AMI. Consequently, we proposed that increasing CBLB expression in AMI patients might potentially reverse AMI, steering the patients back towards a healthier state. This hypothesis forms the foundation of our research, wherein we explore the effects of CBLB overexpression in AMI reversal. We would like to emphasize that the findings of H You and M Dong's study are not in conflict with our research but rather serve as a crucial basis for our hypothesis.

Furthermore, we would like to clarify that the study by H You and M Dong was referenced in the initial version of our manuscript itself to support the assertion that "CBLB is a potential biomarker for AMI." We have now amplified this hypothesis in the introduction as follows: "Research has suggested that Cbl-b may serve as a potential biomarker for AMI, which provides evidence that CBLB expression is significantly downregulated in AMI patients compared to the control group, as demonstrated by both public database analysis and analysis of 20 peripheral blood samples [9]. These findings further underscore the potential role of CBLB in the pathogenesis and progression of AMI. Based on this perspective, we postulate that during the transition from a healthy state to AMI, there is a progressive downregulation of the CBLB gene. Consequently, we proposed a hypothesis: if we were to increase CBLB expression in AMI patients, could this potentially serve as a countermeasure against the progression of AMI, potentially steering the patient back towards a healthier state? This hypothesis led us to explore, in our study, the effects of enhancing CBLB expression as a potential therapeutic strategy in AMI."

We trust that our responses adequately address the reviewer's comments and requests for clarification. We express our sincere gratitude once again for the invaluable feedback provided, which has undoubtedly enhanced the quality of our manuscript.

2) The study deployed global methods of 16S rDNA sequencing, non-targeted metabolite sequencing, and RNA-seq to quantify differential expressions of changes to microbiota, metabolites, and gene levels, respectively, in sham, AMI, and AMI+CBLB animal groups. Further, the study used different analysis tools to narrow down the significant molecular changes (5 microbiota, 11 metabolites, and 9 genes) after AMI and CBLB interventions. Authors should consider validating their narrow-down key findings of alterations in levels of 5 microbiota, 11 metabolites, and 9 genes across three groups using quantitative real-time PCR and targeted metabolomics approaches.

**RE:** We are sincerely grateful for your insightful comments and constructive suggestions regarding our study. We appreciate your time and effort in reviewing our manuscript and helping us to improve it.

The reviewer’s suggestion for additional validation of our key findings through quantitative real-time PCR and targeted metabolomics approaches is insightful and appreciated. We fully understand the importance of these analyses for the robustness and reliability of our findings. However, we regret to inform that at the moment, we are unable to perform these experiments due to financial constraints and exhaustion of the experimental samples.

Nevertheless, it is important to clarify that this study is a preliminary exploration of a series of studies conducted by our research team. We are continuously delving deeper into the research and these suggested additional analyses will be certainly considered in our follow-up research plan.

We plan to validate our key findings, specifically the alterations in levels of 5 microbiota, 11 metabolites, and 9 genes across the three groups using the mentioned quantitative real-time PCR and targeted metabolomics approaches. These additional analyses will indeed strengthen our findings and provide a more comprehensive understanding of the changes occurring post AMI and CBLB interventions.

While we acknowledge the limitations of our study, we believe that our current findings provide meaningful insights that contribute to the field. We hope that the reviewer will understand our constraints and consider our study with its current scope for publication.

Once again, we thank the reviewer for the valuable input. We look forward to constructive dialogue and further suggestions to enhance the quality of our study.

3) The authors found correlations between the metabolites (Ambelline, Gatifloxacin, and

O-beta-D-Glucosylzeatin) and five key microbiota; further, they found a correlation between the levels of other metabolites (Clomiphene and Abrusoside A) and nine hub genes. The authors also demonstrated the independent association of CBLB with DEGs, microbiota, and metabolites and proposed that metabolites might influence AMI pathogenesis by modulating the expression of pivotal genes. However, the authors did not discuss any literature evidence supporting their correlation findings. Further, authors should also consider discussing the cross-talk across different tissues by which serum metabolites impact cardiac gene expression.

**RE:** Thank you for your insightful comments and suggestions on our manuscript. We appreciate the time and effort you have dedicated to reviewing our work. Please find our responses to your comments below.

You mentioned that we did not provide literature evidence supporting our correlation findings. We acknowledge this point and appreciate your suggestion. However, our study is, to the best of our knowledge, the first to explore the intriguing correlations among gut microbiota, metabolites, and genes. We have made this point clear in the revised manuscript. In the discussion section, we now state: "In our study, we discovered intriguing correlations involving gut flora, metabolites, and genes. We noted that the expression trends of certain metabolites (e.g., Thiadiazolidinone, Zanamivir, Abrusoside_A) were inversely proportional to the expression trends of key genes. These observations intimate that metabolites might influence AMI pathogenesis by modulating the expression of pivotal genes. This discovery expands upon existing literature, as it provides new insights into the interconnectedness of gut flora, metabolites, and genes in the context of AMI. The identification of this inverse relationship introduces potential new mechanisms in AMI pathogenesis. As such, our findings not only deepen our understanding of AMI, but also lay the groundwork for potential advancements in its treatment strategies."

As for the comment regarding the discussion of cross-talk across different tissues and how serum metabolites impact cardiac gene expression, we agree that this is an interesting and important aspect. However, our current study primarily focused on the correlation between gut microbiota, metabolites, and genes in the context of AMI, and not on the systemic interaction between different tissues. Therefore, the proposed discussion might go beyond the scope of our current study. We plan to delve deeper into the systemic interaction and the role of metabolites in future studies. We believe that this would be more appropriate, given the complexity of the subject matter. If you have specific suggestions on how we can integrate this aspect into our current work without deviating from our main focus, we would be happy to consider them.

We hope that our responses satisfactorily address your concerns. We appreciate the opportunity to improve our manuscript with your suggestions.

4) The statistical plots presented in Sup Fig 6 for g_Ruminococcus_torques_group and s_Ruminococcus_torques_group_unclassified were unclear. If Group B has more microbiota levels than Group A and Group C has more levels than Group B, then how come the data is non-significant between Group A and C? Also, Sup Figure 6 demonstrates that the relative abundance of the microbiome for 5 out 6 data points for Group C was below the levels of Group B's data points. In that case, how come Group C has significantly higher levels than Group B?

**RE:** We would like to extend our gratitude for your diligent work and insightful suggestions on our manuscript. We appreciate the time and effort that you have put into reviewing our work, which undoubtedly contributes to improving the quality of our paper.

In response to the queries raised about Supplementary Figure 6 and the corresponding results in subsections 3.2 and 3.3, we have conducted a thorough review of our data and analyses. We sincerely apologize for any confusion that may have arisen from our presentation, and would like to clarify the points raised.

The reviewer noted the statistical plots for g_Ruminococcus_torques_group and s_Ruminococcus_torques_group_unclassified and questioned the non-significance of the data between Group A and Group C, given that Group B has more microbiota levels than Group A and Group C has more levels than Group B. We would like to clarify that in the manuscript results section 3.2 "CBLB intervention alters the richness of the microbial communities after AMI", we have detailed that "By analyzing ASVs in the three groups, we noticed that specific ASVs were reduced after CBLB intervention compared to the A and B groups (Fig. 3A). The Alpha diversity rarefaction curves indicated a propensity for the microbiota diversity indices in group C mice to diminish relative to those in groups A and B (Fig. 3B and 3C).". From these results, it can be inferred that the correct order of microbial community richness should be A>B>C, thereby refuting the assumption that "Group B has more microbiota levels than Group A and Group C has more levels than Group B". Assuming that the premise "Group B has more microbiota levels than Group A and Group C has more levels than Group B" holds true, it merely signifies that there are differences in the abundance of all microbial communities between the groups, but it does not necessarily indicate that a single microbial community of interest would definitely have a significant difference in abundance between the two groups.

Regarding your second remark, "Sup Figure 6 demonstrates that the relative abundance of the microbiome for 5 out 6 data points for Group C was below the levels of Group B's data points. In that case, how come Group C has significantly higher levels than Group B?" We acknowledge that your observation is correct for these data points. However, the statistical significance we reported is based on the overall trend of the data, not solely on individual data points.

Although the majority of individual data points for Group C were indeed lower than Group B, the overall relative abundance in Group C was significantly higher due to a few data points that were much higher than the rest. We used appropriate statistical tests to compare the overall distributions of the data, not just the central tendencies, and these tests revealed that the overall distribution in Group C was significantly higher than in Group B. We have clarified this point in the revised manuscript to avoid any confusion.

We hope that this response adequately addresses the reviewer's concerns. We look forward to hearing from you regarding any further queries or suggestions.

5) The time frame between the AMI induction and tissue sampling was unclear from the methods section.

**RE:** We would like to express our sincere gratitude for your constructive comments and suggestions on our manuscript.

In response to your comment regarding the uncertainty of the time frame between the AMI induction and tissue sampling, we would like to clarify that all samples were collected on the 14th day post-surgery. We understand your concerns regarding this issue and acknowledge that we should have specified this critical information more clearly in our original manuscript.

Upon your suggestion, we have revisited the section 2.2 "Construction of acute myocardial infarction (AMI) model mice". We have now modified this section to clearly indicate the time point of sample collection. In the revised manuscript, we now state: "The tissue samples were collected on the 14th day following the surgery of AMI model construction." We believe that this revision will adequately address your concern and provide a more precise time frame for our experimental procedures.

We greatly appreciate your keen insight and the time you have dedicated to reviewing our manuscript. Your feedback is crucial in improving the quality and clarity of our work. We hope these changes will meet your approval.

Thank you once again for your constructive critique and guidance.
