# Supplementary material for: Exploring the role of CBLB in acute myocardial infarction: transcriptomic, microbiomic, and metabolomic analyses
Source: J Transl Med. 2024 Jul 14;22:654. doi: 10.1186/s12967-024-05425-y (PMC11247792; doi:10.1186/s12967-024-05425-y)
Supplement: Supplementary file 17 — Supplementary Material 17 [file 12967_2024_5425_MOESM17_ESM.docx]

Dear Editor of Journal of Translational Medicine,

I am submitting the enclosed manuscript, "**Exploring the Role of CBLB in Acute Myocardial Infarction: A Comprehensive Study on Transcriptome, Gut Microbiota, and Metabolomics**", for consideration of publication in the ***Journal of Translational Medicine***.

The purpose of our research is to shed light on the significant role of CBLB in Acute Myocardial Infarction (AMI) by examining the interactions between transcriptome, gut microbiota, and metabolomics in CBLB-intervened AMI mice. Our findings provide a novel insight into the potential efficacy of multiple differential genes, metabolites, and microbiota in the regulation of AMI after CBLB intervention, which could form a substantial basis for further exploration of the regulatory mechanism of CBLB in AMI.

Our study fits perfectly within the scope of the Journal of Translational Medicine, as it is dedicated to the connection between laboratory and clinical research. We believe that our work could contribute significantly to the field as it presents a comprehensive study involving transcriptomics, gut microbiota, and metabolomics to understand the role of CBLB in AMI.

We confirm that all authors have read and approved the final manuscript and its submission to your esteemed journal. Additionally, there are no competing interests to declare. Lastly, the work described in this manuscript is original, has not been published previously, and is not currently under consideration for publication elsewhere.

We appreciate your consideration of our manuscript for publication in the Journal of Translational Medicine. We believe the readers of the journal will find it informative and valuable. We look forward to your positive response.

Sincerely,

Wenqi Han, M.D.

Department of Cardiovascular Medicine, Shaanxi Provincial People's Hospital, Xi'an 710068, Shaanxi, China

Email: hwq315@163.com
